# Directed Diastereoselective Cyclopropanation and Epoxidation of Alkenyl Cyclopropyl Carbinol Derivatives

**DOI:** 10.1021/acs.orglett.2c03305

**Published:** 2022-11-10

**Authors:** Anthony Cohen, Yogesh Siddaraju, Ilan Marek

**Affiliations:** Schulich Faculty of Chemistry, Technion − Israel Institute of Technology, Haifa 3200009, Israel

## Abstract

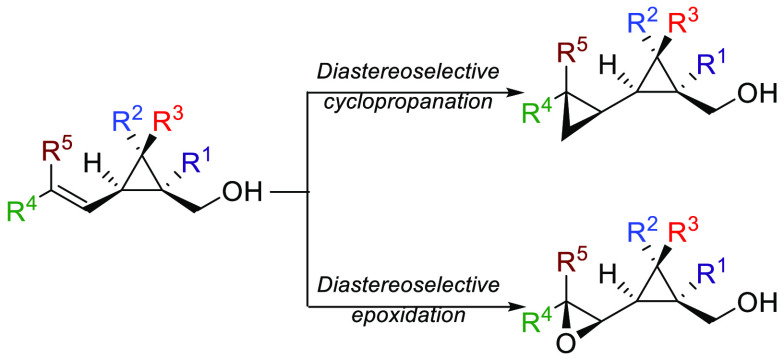

We report the directed diastereoselective Simmons–Smith cyclopropanation and vanadium-catalyzed epoxidation reactions of alkenyl cyclopropyl carbinol derivatives. The reaction furnished densely substituted stereodefined bicyclopropanes and cyclopropyl oxiranes as a single diastereomer in each case. The remarkable selectivity is obtained thanks to the rigidity of the cyclopropyl core, allowing diastereoselective reactions on the alkenyl moiety. This emphasizes the uniqueness of the cyclopropyl ring as a central platform in stereoselective synthesis.

The stereoselective synthesis of numerous contiguous stereocenters represents a constant chemical challenge: the higher the number of adjacent stereocenters, the harder is the synthesis.^1^ Although various strategies have recently appeared expanding the portfolio of tools available to practitioners,^2−6^ the synthesis of sophisticated organic structures requires the continuous development of new methodologies that control all selectivity issues of a given reaction. Among the several general methods to control the selectivity of a reaction,^7−10^ the substrate-directed approach occupies a special position.^11−14^ The key features of this success are the level of predictability, the high diastereo- and enantioselectivity, and the latent potential functionality of the products for subsequent transformations. Indeed, in this approach, a polar functional group (directing group), usually situated in close proximity of the reactive site, induces the stereoselective step and can be successively manipulated toward the formation of more complex molecular architecture.^7−14^

Two of the most classical examples employ allylic alcohol derivatives for the diastereo- and/or enantioselective epoxidation^12,13^ or cyclopropanation reactions,^15,16^ among others,^11^ underlining the critical importance of the position of the directing group at a close vicinity of the reactive part of the molecule.^14^ When the distance between the polar group and the reactive alkene increases, the face stereodifferentiation becomes more difficult and the level of selectivity decreases. To answer such limitations, several strategies have been developed for homoallylic alcohols.^13^

During our studies on the diastereoselective synthesis of polysubstituted cyclopropanes^17−21^ as a new source of stereodefined acyclic products,^22^ we have recently shown that alkenyl cyclopropyl carbinol derivatives **1** exhibit an excellent directing effect for the palladium-catalyzed tandem Heck addition and ring opening reaction^23−28^ as well as for diboration reactions^29^ (Scheme 1a). Even more distant alkenyl biscyclopropyl carbinol **2** led to a completely diastereoselective Heck addition reaction before the subsequent selective double ring-opening.

**Scheme 1:**
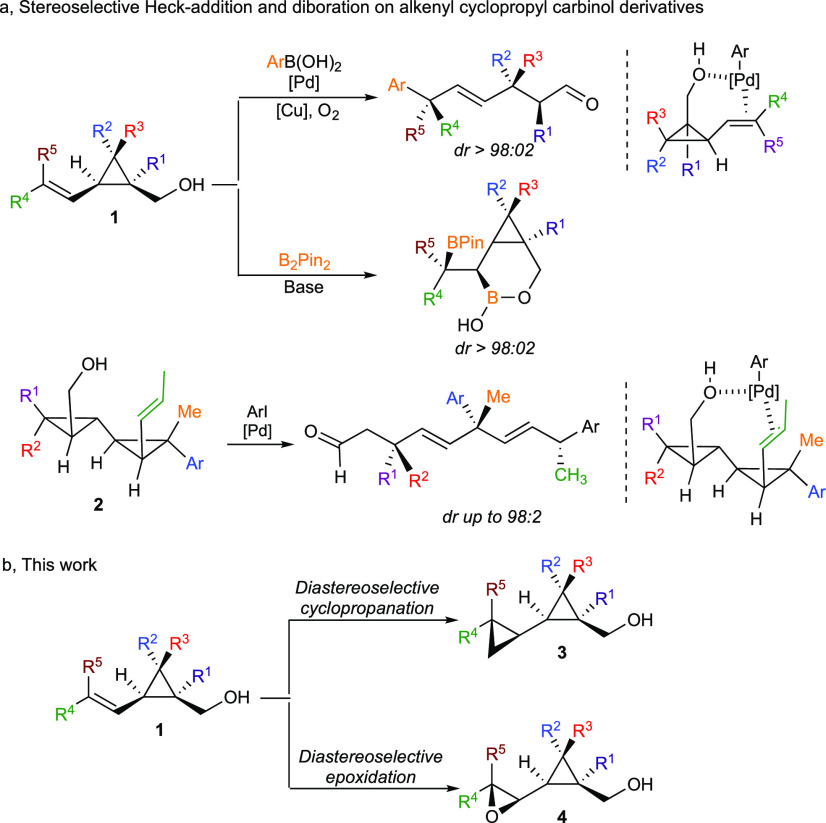
Directed Transformations of Alkenyl Cyclopropyl Carbinol Derivatives

Although the functional group is in a 3,4 position in **1** and 5,6 position in **2**, the particular molecular structure of these alkenyl cyclopropyl carbinols induces a preferred *s-trans*(23) conformation toward the *s-cis* conformation, favoring a diastereofacial choice in the addition reaction (Scheme 1a). Based on this diastereoselective Pd-catalyzed Heck addition on alkenyl cyclopropyl carbinols, we were initially interested in extending this substrate-directed transformation to the challenging synthesis of bicyclopropyl carbinols **3**. Our previous approach based on a double-diastereoselective carbometalation reaction of two differently substituted cyclopropenes had the major drawback of producing two geometrical isomers, even if each one was diastereomerically pure (**2** in Scheme 1a).^26^ To access stereodefined alkenyl cyclopropyl carbinol derivatives **1**, we used our previously established copper-catalyzed diastereoselective carbometalation reaction of cyclopropenes followed by a Pd-catalyzed cross-coupling reaction (Scheme 2).^24^ Cyclopropenes were easily prepared via the standard Rh-catalyzed decomposition of diazo esters in the presence of alkynes^30^ (see the Supporting Information for full details). A simple reduction of the ester provides the desired alkenyl cyclopropyl carbinols **1**.

**Scheme 2:**
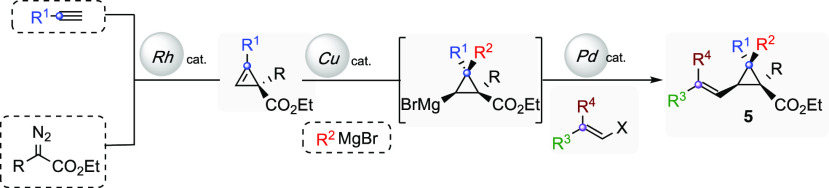
Regio- and Diastereoselective Preparation of Alkenyl Cyclopropyl Esters

With alkenyl cyclopropyl carbinol derivatives **1** (and **6** and **7**) as well as esters **5** at our disposal, we started to investigate if a distant functional group could direct the cyclopropanation reaction using the classical Simmons–Smith–Furukawa condition.^31^ To our delight, subjecting our model substrate **1a** (R = R^4^ = R^5^ = H, R^2^ = Me, R^1^ = Bu, R^3^ = Bu) to an equimolar mixture of diiodomethane and diethyl zinc in DCM at 0 °C resulted in a quantitative formation of bicyclopropyl carbinol **3a** with excellent diastereoselectivity (88% yield and dr > 98:02, Scheme 3). This result indicates that the in situ formed zinc carbenoid must be preassociated with the alcoholate functional group to stereodirect the carbenoid toward one of the diastereotopic faces of the alkene.^31^

**Scheme 3:**
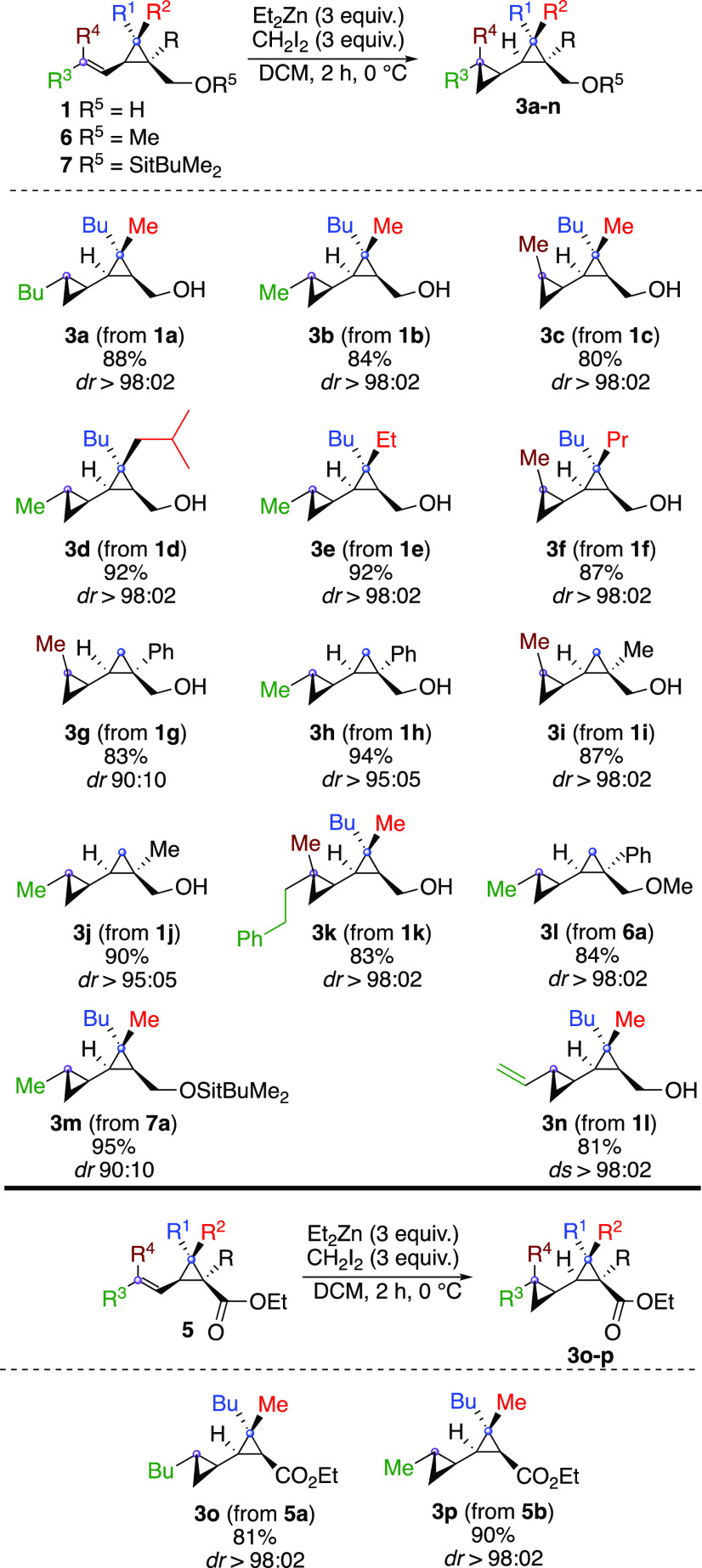
Diastereoselective Cyclopropanation of Alkenyl Cyclopropyl Carbinol Derivatives

Using this optimized condition, we demonstrated that various R^2^ primary (**3a**, **3b**, **3c**, **3e**, and **3f**) as well as secondary alkyl groups (**3d**) are well tolerated in the reaction without altering yields and selectivities. Similarly, the stereochemistry (*E* or *Z*) of the alkenyl side chain has no effect on the stereochemical outcome of the reaction (compare **3b** and **3c**, Scheme 3). Interestingly, the presence of the quaternary carbon stereocenter can be located at different places without changing the chemistry (compare **3b** with **3h** and **3b** with **3k**, Scheme 3). Importantly, the reaction is stereospecific, with *E* and *Z*-alkenyl moieties converted into their respective *trans*- and *cis*-cyclopropanes with a complete stereospecificity. Interestingly, sterically crowded trisubstituted olefins are smoothly converted under our standard conditions to bicyclopropane **3k** bearing two quaternary carbon stereocenters. If the double bond is one more carbon away from the cyclopropyl core, the reaction is not diastereoselective anymore.

Finally, we decided to test other directing groups as potential promoters of the cyclopropanation reaction. We were pleased to observe that alkenyl cyclopropyl methyl ether **6a** as well as alkenyl cyclopropyl silyl ether **7a** were successfully converted to **3l** and **3m**, respectively, with excellent yields and diastereoselectivities. Subjecting compound **1l** possessing a dienyl moiety to the developed cyclopropanation conditions resulted in an efficient and selective transformation to **3n** in excellent yield and diastereospecificity.^32^

The lack of cyclopropanation at the terminal double bond further demonstrates the strong directing effect of the hydroxyl group. Similarly, alkenyl cyclopropyl esters **5a** and **5b** proved to direct the reaction equally well to give the expected products **3n** and **3p** as single diastereomers. Based on the outstanding abilities of the functional group to direct the cyclopropanation reaction of alkenyl cyclopropyl carbinols **1**, we then set out to extend the strategy to other important synthetic transformation. We therefore turned our attention to the diastereoselective epoxidation reaction. When *m*CPBA or *t*BuOOH was added to **1b**, a non-diastereoselective epoxidation reaction occurred to deliver the epoxide **4a** in a 2:1 ratio. This highlights the insufficient direction provided by hydrogen-bonding interactions with the carbinol group and the importance of metal-mediated epoxidation reactions for achieving high stereoselectivity.

Indeed, when the same reaction was performed on **1b** but in the presence of 10 mol % of vanadium acetylacetonate,^33−35^**4a** was obtained in excellent yield with complete diastereoselectivity (Scheme 4). When the opposite geometrical isomer was treated in the same conditions, the epoxide **4b** was obtained with a similar selectivity featuring the specificity of the transformation. The degree of substitution has no effect on the diastereoselectivity of the process as trisubstituted alkenyl cyclopropyl carbinol **1k** was epoxidized as a single diastereomer (formation of **4d**, Scheme 4).

**Scheme 4:**
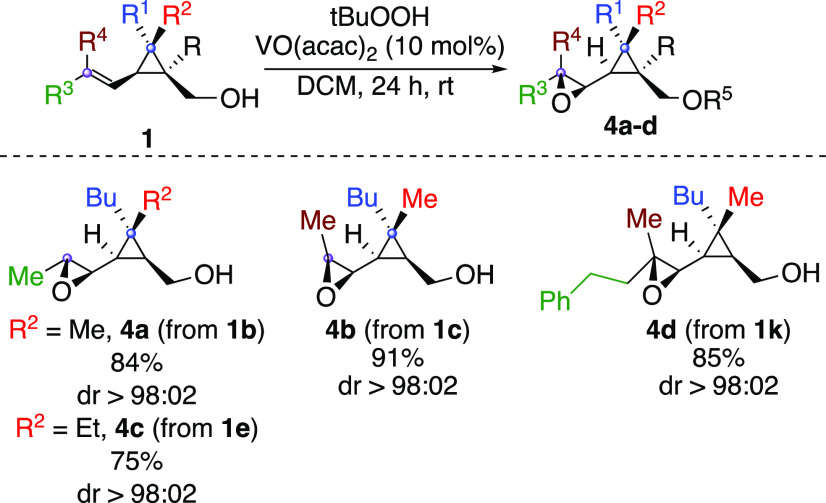
Vanadium-Catalyzed Epoxidation Reaction of Alkenyl Cyclopropyl Carbinol

In conclusion, the directed diastereoselective Simmons–Smith cyclopropanation reaction of alkenyl cyclopropyl carbinol derivatives as well as alkenyl cyclopropyl esters provides the corresponding polysubstituted stereodefined bicyclopropanes as a single diastereomer in each case. The same trend holds for the vanadium-catalyzed epoxidation reaction. The rigidity of the cyclopropyl core allows diastereoselective reactions on the alkenyl moiety, emphasizing the uniqueness of the cyclopropyl ring as a central platform in stereoselective synthesis.

## Notes

The data underlying this study are available in the published article and its online Supporting Information.

## Supporting Information Available

Experimental procedures, characterization data for all new compounds, along with copies of spectra. Computational methods and data, geometries of computed intermediates and transition states (PDF)
