# Supplementary material for: Directed Diastereoselective Cyclopropanation and Epoxidation of Alkenyl Cyclopropyl Carbinol Derivatives
Source: Org Lett. 2022 Nov 10;24(45):8322–5. doi: 10.1021/acs.orglett.2c03305 (PMC9743385; doi:10.1021/acs.orglett.2c03305)
Supplement: Supplementary file 1 — ol2c03305_si_001.pdf [file ol2c03305_si_001.pdf]

Supporting information

Directed Diastereoselective Cyclopropanation and Epoxidation of Alkenyl Cyclopropyl Carbinols Derivatives

Anthony Cohen, Yogesh Siddaraju and Ilan Marek

Schulich Faculty of Chemistry

Technion – Israel Institute of Technology,

Technion City

Haifa, 32000 (Israel)

E-mail: chilanm@technion.ac.il

Table of Contents

Experimental procedures S-3

General information S-3

Procedures for the preparation of cyclopropenes S-3

Procedures for the preparation of alkenyl halides S-3

Procedure for the preparation of alkenyl cyclopropyl carbinols **1** (procedure A) S-4

Procedure for the preparation of alkenyl cyclopropyl carbinols **1** (procedure B) S-5

Procedure for the preparation of cyclopropenyl lactol **12** S-7

Procedure for the preparation of dienyl cyclopropyl carbinols **1l** S-8

Procedure for the Simmons-Smith cyclopropanation of alkenyl cyclopropyl carbinols **1** S-9

Procedure for the epoxidation of alkenyl cyclopropyl carbinols **1** S-13

References S-15

NMR spectra for new compounds S-16

Experimental procedures

General information

Unless stated otherwise, reactions were conducted in a flame-dried glassware under a positive pressure of argon. Ether and THF were dried from Pure-Solv® Purification System (Innovative Technology©). All other commercially obtained reagents were used as received. Thin-layer chromatography (TLC) was conducted with E. Merck silica gel 60 F254 pre-coated plates (0.25 mm) and visualized by exposure to UV light (254 nm) or stained with anisaldehyde, phosphomolybdic acid, or potassium permanganate solutions. Column chromatography was performed using Fluka silica gel 60 Å (40-63mm, 230-400 mesh). NMR spectra were recorded on Bruker spectrometers (AVIII400) and are reported relative to deuterated solvent signals. Chemical shifts are reported in parts per million (ppm) with respect to the residual solvent signal $CDCl_3$ (^1H NMR: δ = 7.26; ^{13}C NMR: δ = 77.16). Peak multiplicities are reported as follows: s = singlet, bs = broad singlet, d = doublet, t = triplet, q = quartet, dd = doublet of doublets, td = triplet of doublets, qd = quartet of doublets, qt = quartet of triplets, m = multiplet. High-resolution mass spectra (HRMS) were obtained by the mass spectrometry facility at the Technion. Reactions were monitored by gas chromatography spectrometry (GC) using an Agilent Technologies 7820A GC with an Agilent Technologies 19091J-413 (30 m × 0.3 mm) column. The diastereomeric purity of the compounds was determined by ^{1}HNMR integration, comparing the ratio between the signals of the main diastereomers and the minor ones.

Procedures for the preparation of cyclopropenes

Cyclopropenyl carbinols were prepared through the rhodium catalyzed decomposition of diazo esters in the presence of terminal alkynes, followed by reduction of the ester using DIBAL. Cyclopropene **10a**[1], **10b**, **10c** and **10d**[2] were synthesized according to previously reported protocols. The compounds were isolated and characterized; the experimental results are in good agreement with the literature reports.

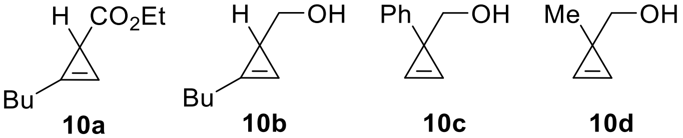

10a **10b** **10c** **10d**

Procedures for the preparation of alkenyl halides

(*Z*)-1-bromoprop-1-ene **11a** and (*E*)-1-bromoprop-1-ene **11b** were used as received from commercial sources. (*E*)-1-bromohex-1-ene **11c**,[3] (E)-(4-iodo-3-methylbut-3-en-1-yl)benzene **11d**[4] were prepared following literature reports and analytic data were in full agreement with the reports.

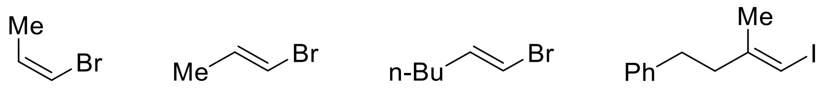

11a **11b** **11c** **11d**

Procedure for the preparation of alkenyl cyclopropyl carbinols 1 (procedure A)

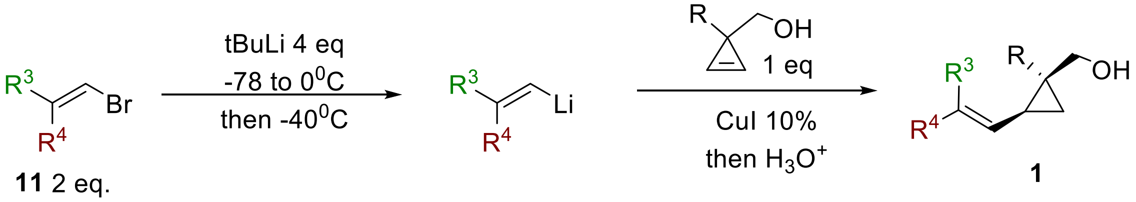

Following a previously reported procedure:[5] In a flamed-dried three-neck flask under argon atmosphere, alkenyl halide **11** (1 equiv., scale of 2-20 mmol) was dissolved in dry Et_2O (0.2 M, 5 mL/mmol of **11**). The solution was cooled to -78 °C using an acetone/dry ice cooling bath, and *tert*-BuLi (1.7 M, 2 equiv.) was added dropwise while maintaining the temperature below -60 °C. The cooling bath was then removed, and the solution was let to warm up to 0 °C. The cooling bath was then replaced and upon reaching a temperature of -40 °C, copper iodide (10 mol%) was added to the reaction mixture and was stirred for 5 minutes. A solution of cyclopropene carbinols (**10c** or **10d**) in Et_2O (1 M, 0.5 equiv., scale of 1-10 mmol, 1 mL of Et_2O/mmol of **10**) was then added and the solution was warmed up to 0 °C over a period of one hour by placing an ice bath. Following the complete conversion of the cyclopropene (conversion monitored by TLC and/or GC), the reaction was quenched with an aqueous saturated solution of ammonium chloride and the aqueous phase was extracted with Et_2O three times. The combined organic phases were dried over anhydrous Na_2SO_4 and concentrated under reduced pressure. The resulting crude mixture was purified by column chromatography using petroleum ether and EtOAc as eluents.

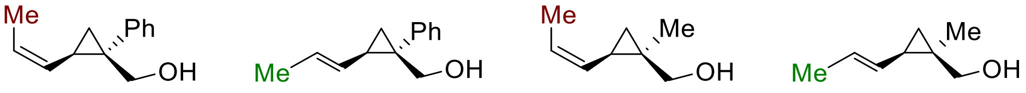

1g **1h** **1i** **1j**

Procedure for the preparation of alkenyl cyclopropyl carbinols 1 (procedure B)

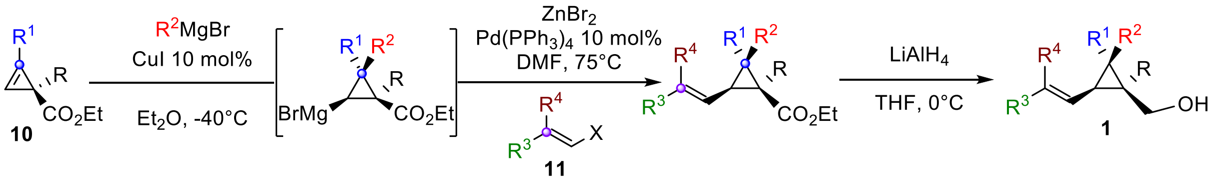

Following a previously reported procedure:[6] In a flamed-dried three-neck flask under Ar, CuI (10 mol%) and cyclopropene **10a** (1 equiv.) were dissolved into Et_2O (8 mL / mmol substrate) and the solution was cooled down to – 45 °C. Then, the Grignard reagent (1.2 equiv., in solution in Et_2O) was added dropwise and the resulting solution was let to stir from -35 °C to -25 °C (conversion monitored by TLC and/or GC). Following the complete conversion of **10a**, the reaction mixture was then cooled to – 50 °C and a solution of flame-dried $ZnBr_2$ (1 M in THF, 1 mL of THF/mmol of $ZnBr_2$, 1.5 equiv.) was slowly added and the resulting white suspension was let to warm-up until -20 °C (c.a. 1.5 h) under vigorous stirring. Then, in a separate flask, [$Pd(PPh_3)_4$] (5 mol%) and vinyl halide **11** (1.2 equiv.) were dissolved into DMF (4 mL/mmol cyclopropene **10**) and transferred to the reaction mixture at – 20 °C. The resulting orange suspension was heated until 75 °C and stirred overnight (monitored by GC). The reaction was quenched with brine and the aqueous phase was extracted with Et_2O three times. The combined organic phases were dried over anhydrous $MgSO_4$ and concentrated under reduced pressure. A column chromatography (gradient eluent: 100% hexane to 2% Et_2O/hexane, stain: phosphomolybdic acid solution) afforded the desired vinyl cyclopropane ester as the major product.

Compounds **1a-f** were subjected to a subsequent reduction step: In a flamed dried three-neck flask under Ar, a solution of the vinyl cyclopropylcarbinol ester (1 equiv.) in THF (5 mL/mmol substrate) was slowly added to a suspension of $LiAlH_4$ (3 equiv.) in THF (5 mL/mmol substrate) under stirring at 0 °C. After completion of the reaction, the reaction mixture was quenched with EtOAc, then carefully a saturated aqueous solution of Rochelle salt (5 mL/mmol substrate) was added and the suspension was vigorously stirred for 1 h at 0 °C. The phases were separated and the aqueous phase was extracted with Et_2O three times. The combined organic phases were dried over anhydrous $MgSO_4$ and concentrated under reduced pressure. Purification of the crude reaction mixture by column chromatography (gradient eluent: 10 – 20% Et2O/hexane) afforded the desired alcohol as a pure compound.

Compounds **1a-d**, **1f**, **5a-b** were previously reported[6,7], the characterization data are in good agreement with literature precedents.

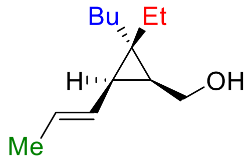

((1S*,2S*,3R*)-2-butyl-2-ethyl-3-((E)-prop-1-en-1-yl)cyclopropyl)methanol (1e)
5 mmol scale, colorless oil, 70% yield, 687 mg. Flash chromatography: 10 – 20% Et_2O/hexane. ^{1}H NMR (400 MHz, CDCl3) δ 5.72 – 5.55 (m, 1H), 5.36 – 5.20 (m, 1H), 3.80 (dd, *J* = 11.5, 7.2 Hz, 1H), 3.67 (dd, *J* = 11.5, 8.6 Hz, 1H), 1.68 (dd, *J* = 6.5, 1.5 Hz, 3H), 1.50 – 1.34 (m, 4H), 1.34 – 1.24 (m, 6H), 1.04 (td, *J* = 8.6, 7.4 Hz, 1H), 0.92 – 0.85 (m, 7H). ^{13}C NMR (101 MHz, CDCl3) δ 127.1, 126.3, 60.1, 37.9, 31.6, 30.3, 29.8, 28.3, 22.9, 19.0, 18.4, 14.2, 10.7. HRMS m/z: (APCI+) $[M-H]^+$, calculated for $C_{13}H_{22}O$; 195.1749; found 195.1744.

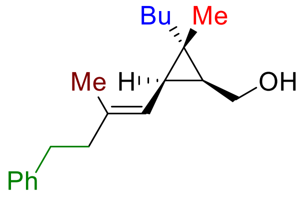

((1S*,2S*,3R*)-2-butyl-2-methyl-3-((E)-2-methyl-4-phenylbut-1-en-1-yl)cyclopropyl)methanol (1k)
5 mmol scale, colorless oil, 63% yield, 902 mg. Flash chromatography: 10 – 20% Et_2O/hexane. ^{1}H NMR (400 MHz, $CDCl_3$) δ 7.23 – 7.15 (m, 2H), 7.14 – 7.02 (m, 3H), 4.83 (dd, *J* = 8.3, 1.2 Hz, 1H), 3.46 (dt, *J* = 19.8, 11.5 Hz, 2H), 2.75 – 2.54 (m, 2H), 2.26 (t, *J* = 7.7 Hz, 2H), 1.67 (d, *J* = 1.0 Hz, 3H), 1.30 – 1.13 (m, 7H), 1.01 – 0.91 (m, 2H), 0.84 (s, 2H), 0.82 (t, *J* = 7.1 Hz, 3H). ^{13}C NMR (101 MHz, $CDCl_3$) δ 142.2, 137.7, 128.5, 128.4, 125.8, 120.1, 60.5, 42.7, 41.6, 34.6, 30.7, 28.9, 25.7, 25.2, 23.0, 16.8, 14.3, 12.9. HRMS m/z: (APCI+) $[M-H]^+$, calculated for $C_{20}H_{29}O$; 285.2213; found 285.2227.

Procedure for the preparation of cyclopropenyl lactol 12

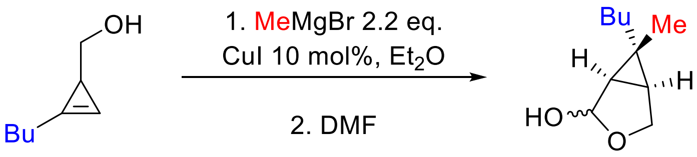

In a flamed-dried three-neck flask under argon atmosphere, CuI (10 mol%, 0.5 mmol, 95 mg) and cyclopropene **10b** (1 equiv., 5 mmol, 631 mg) were dissolved in Et_2O (40 mL, 0.125 M) and the solution was cooled down to – 45 °C. Then, methyl magnesium bromide (2.2 equiv., 3M solution in Et_2O, 11 mmol, 3.67 mL) was added dropwise and the resulting solution was stirred from -35 °C to -25 °C (conversion monitored by TLC and/or GC). The acetone bath was removed and replaced with an ice bath. Following the complete conversion of **10b**, a solution of dry DMF (1 M in THF (50 mL of THF), 10 equiv., 50 mmol, 3.85 mL) was slowly added and the resulting white suspension was warm-up to rt (c.a. 3 h) under vigorous stirring. The reaction was quenched with an aqueous saturated solution of NH_4Cl and the aqueous phase was extracted with Et_2O three times. The combined organic phases were dried over anhydrous Na_2SO_4 and concentrated under reduced pressure. Purification of the crude reaction mixture by column chromatography afforded the desired alcohol as a pure compound (gradient eluent: 20 – 40% Et_2O/hexane).

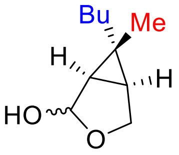

(1R*,5S*,6R*)-6-butyl-6-methyl-3-oxabicyclo[3.1.0]hexan-2-ol (12)
5 mmol scale, colorless oil, 57% yield, 485 mg. Flash chromatography: 20 – 40% Et_2O/hexane. ^1H NMR (400 MHz, $CDCl_3$) δ 5.12 (d, *J* = 4.3 Hz, 1H), 4.07 (dd, *J* = 8.6, 3.3 Hz, 1H), 3.67 (dd, *J* = 9.4, 6.7 Hz, 2H), 1.23 (ddd, *J* = 17.3, 10.8, 5.6 Hz, 6H), 0.91 (d, *J* = 2.7 Hz, 3H), 0.83 – 0.78 (m, 5H). ^{13}C NMR (101 MHz, $CDCl_3$) δ 97.9, 66.8, 39.8, 34.8, 28.7, 27.4, 22.9, 22.5, 14.1, 10.1. HRMS m/z: (APCI+) $[M-OH]^+$, calculated for $C_{10}H_{17}O$; 153.1279; found 153.1268.

Procedure for the preparation of dienyl cyclopropyl carbinols 1l

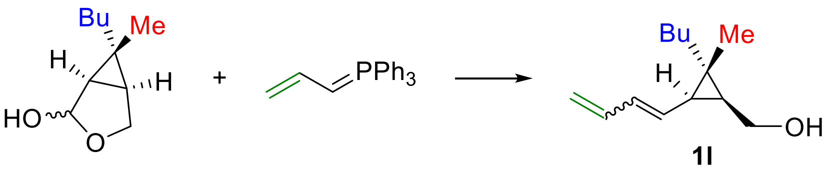

1l

Potassium tert-butoxide (3 equiv., 7.5 mmol, 842 mg) was added to a suspension of triphenyl[(*E*)-3-phenyl-2-propenyl]phosphonium chloride (2 equiv., 5 mmol, 1694 mg) in anhydrous diethyl ether (0.3 M, 16.67 mL of Et_2O) at rt and under an argon atmosphere. After stirring for 15 minutes the solution was cooled to 0 °C and a solution of the lactol **12** (1 equiv., 2.5 mmol, 423 mg) in anhydrous THF (0.6 M, 4.25 mL THF) was added. After stirring for 48 h at rt the solution was diluted by half with hexanes and filtered through silica (3 cm). The silica was washed with a solution of 2:3 ethyl acetate/hexanes until all dienes had been removed. The volatiles were then removed from the combined filtrates and were evaporated under reduced pressure. The resulting residue was purified by flash chromatography (gradient eluent: 10 – 20% Et2O/hexane) to give the desired diene **1l.**

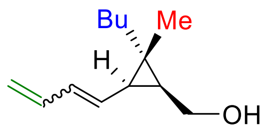

((1S*,2S*,3R*)-3-(buta-1,3-dien-1-yl)-2-butyl-2-methylcyclopropyl)methanol (1l)
2.5 mmol scale, colorless oil, 81% yield, 394 mg, mixture of isomers – *E:Z=2:1*. Flash chromatography: 10 – 20% Et_2O/hexane. ^1H NMR (400 MHz, CDCl3) δ 6.73 – 6.54 (m, 1H), 6.29 – 6.08 (m, 1H), 6.02 (t, J = 11.0 Hz, 1H), 5.40 (dd, J = 14.5, 10.0 Hz, 1H), 5.24 – 4.92 (m, 1H), 4.82 (dd, J = 9.4, 1.3 Hz, 1H), 3.69 – 3.48 (m, 2H), 1.64 – 1.51 (m, 1H), 1.39 (s, 1H), 1.31 (dd, J = 17.5, 7.9 Hz, 1H), 1.18 (dddd, J = 16.7, 11.3, 7.4, 4.5 Hz, 3H), 1.14 – 0.99 (m, 1H), 0.95 (d, J = 7.0 Hz, 1H), 0.78 (td, J = 6.9, 3.0 Hz, 2H). ^{13}C NMR (101 MHz, CDCl3) δ 137.1, 132.8, 132.5, 131.3, 131.0, 127.7, 117.5, 114.4, 60.14, 60.12, 42.61, 42.57, 32.4, 32.0, 30.3, 28.8, 28.7, 26.9, 26.5, 25.7, 22.91, 22.88, 14.3, 13.0, 12.9. HRMS m/z: (APCI+) [M-H]+, calculated for $C_{13}H_{21}O$; 193.1587; found 193.1600.

Procedure for the Simmons-Smith cyclopropanation of alkenyl cyclopropyl carbinols 1

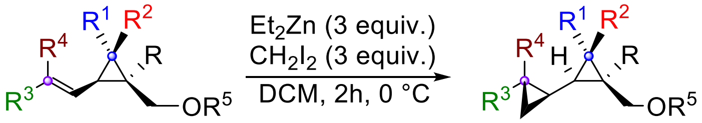

In a flamed-dried three-neck flask under argon atmosphere, diiodomethane (CH_2I_2, 3 equiv., 1.5 mmol, 402 mg) was dissolved in dry DCM (0.1 M, 10 mL of DCM/ mmol of CH_2I_2). The solution was cooled to 0 °C using an ice bath. A solution of diethylzinc (Et_2Zn, 3 equiv., 1M in hexane, 1.5 mL) was added dropwise to the solution, resulting in the formation of a white suspension. The suspension was stirred for 10 minutes and then a solution of alkenyl cyclopropane **1** in DCM (0.5 mmol, 1 M, 1 mL of DCM/ mmol of **1**) was added dropwise. The ice bathe was removed, and the reaction was stirred for 3 h. Upon completion, as monitored by GC analysis of hydrolyzed aliquots, the reaction was quenched with an aqueous saturated solution of NH_4Cl and extracted three times with DCM. The combined organic phases were dried over anhydrous Na_2SO_4 and concentrated under reduced pressure. Column chromatography (gradient eluent: 10 – 25% Et2O/hexane) afforded the desired alcohol as a pure compound.

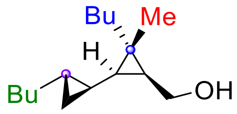

((1R*,1'S*,2S*,2'S*,3S*)-2',3-dibutyl-3-methyl-[1,1'-bi(cyclopropan)]-2-yl)methanol (3a)
0.5 mmol scale, colorless oil, 88% yield, 105 mg. Flash chromatography: 10 – 25% Et_2O/hexane. ^1H NMR (400 MHz, $CDCl_3$) δ 3.88 – 3.55 (m, 2H), 1.36 – 1.14 (m, 12H), 1.08 (s, 3H), 0.88 (dd, *J* = 12.9, 6.7 Hz, 6H), 0.80 (dd, *J* = 16.4, 8.2 Hz, 1H), 0.61 – 0.50 (m, 1H), 0.36 – 0.29 (m, 1H), 0.26 (dt, *J* = 8.9, 4.6 Hz, 1H), 0.21 – 0.12 (m, 1H), 0.08 (t, *J* = 8.9 Hz, 1H). ^{13}C NMR (101 MHz, $CDCl_3$) δ 60.8, 42.7, 34.1, 31.8, 31.5, 29.0, 28.4, 23.4, 22.9, 22.6, 19.9, 14.3, 14.2, 13.4, 13.0, 12.4. HRMS m/z: (APCI+) $[M+H]^+$, calculated for $C_{16}H_{31}O$; 239.2369; found 239.2356.

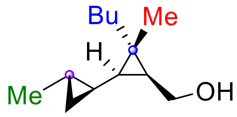

((1R*,1'S*,2S*,2'S*,3S*)-3-butyl-2',3-dimethyl-[1,1'-bi(cyclopropan)]-2-yl)methanol (3b)
The reaction was conducted according to the general procedure described above: In a flamed-dried three-neck flask under argon atmosphere, diiodomethane (CH_2I_2, 3 equiv., 3 mmol, 804 mg) was dissolved in dry DCM (0.1 M, 30 mL). The solution was cooled to 0 °C using an ice bath. A solution of diethylzinc (Et_2Zn, 3 equiv., 1M in hexane, 3 mL) was added dropwise to the solution, resulting in the formation of a white suspension. The suspension was stirred for 10 minutes and then a solution of alkenyl cyclopropane **1b** (colorless oil, 1 mmol 182 mg, 1 equiv.) in DCM (1M, 1 m) was added dropwise. The ice bath was removed, and the reaction was stirred for 3 h. Upon completion, as monitored by GC analysis of hydrolyzed aliquots, the reaction was quenched with an aqueous saturated solution of NH_4Cl and extracted three times with DCM. The combined organic phases were dried over anhydrous Na_2SO_4 and concentrated under reduced pressure. Column chromatography (gradient eluent: 10 – 25% Et_2O/hexane) afforded the desired alcohol as a pure compound: colorless oil, 84% yield, 165 mg. ^1H NMR (400 MHz, $CDCl_3$) δ 3.78 – 3.66 (m, 2H), 1.32 – 1.09 (m, 3H), 1.05 (s, 1H), 1.01 (d, *J* = 6.0 Hz, 2H), 0.86 (t, *J* = 7.0 Hz, 1H), 0.78 (dd, *J* = 14.7, 6.7 Hz, 1H), 0.55 (qt, *J* = 11.3, 5.9 Hz, 1H), 0.32 (dd, *J* = 8.1, 4.3 Hz, 1H), 0.26 – 0.20 (m, 1H), 0.15 – 0.04 (m, 1H). ^{13}C NMR (101 MHz, $CDCl_3$) δ 60.7, 42.6,

31.3, 29.0, 28.5, 23.4, 22.9, 19.0, 14.6, 14.3, 13.78, 13.75, 13.0. HRMS m/z: (APCI+) [M-OH]+, calculated for $C_{13}H_{23}$; 179.1800; found 179.1797.

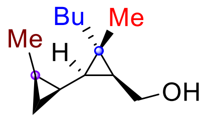

((1R*,1'S*,2S*,2'R*,3S*)-3-butyl-2',3-dimethyl-[1,1'-bi(cyclopropan)]-2-yl)methanol (3c)
1 mmol scale, colorless oil, 80% yield, 157 mg. Flash chromatography: 10 – 25% Et_2O/hexane. ^1H NMR (400 MHz, $CDCl_3$) δ 3.81 – 3.68 (m, 2H), 1.34 – 1.24 (m, 6H), 1.09 – 1.04 (m, 6H), 0.90 – 0.85 (m, 5H), 0.69 (td, *J* = 8.4, 4.2 Hz, 1H), 0.46 (ddd, *J* = 17.2, 8.6, 5.3 Hz, 1H), 0.32 (t, *J* = 9.0 Hz, 1H), -0.15 (dd, *J* = 9.6, 5.3 Hz, 1H). ^{13}C NMR (101 MHz, $CDCl_3$) δ 60.7, 42.9, 28.9, 28.4, 26.3, 23.2, 23.1, 14.3, 14.2, 13.05, 12.96, 10.6, 9.9. HRMS m/z: (APCI+) [M-OH]+, calculated for $C_{13}H_{23}$; 179.1794; found 179.1794.

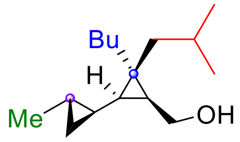

((1R*,1'S*,2S*,2'S*,3R*)-3-butyl-3-isobutyl-2'-methyl-[1,1'-bi(cyclopropan)]-2-yl)methanol (3d)
0.5 mmol scale, colorless oil, 92% yield contains 7% sm (accounted in yield determination), 118 mg. Flash chromatography: 10 – 25% Et_2O/hexane. ^1H NMR (400 MHz, CDCl3) δ 3.76 (dd, J = 11.4, 6.6 Hz, 1H), 3.65 – 3.58 (m, 1H), 1.75 (dt, J = 13.5, 6.7 Hz, 1H), 1.30 – 1.27 (m, 2H), 1.25 – 1.19 (m, 4H), 1.00 (s, 2H), 0.92 (d, J = 6.6 Hz, 3H), 0.85 (dd, J = 6.6, 3.6 Hz, 5H), 0.77 (d, J = 6.8 Hz, 1H), 0.57 (dt, J = 10.8, 5.4 Hz, 1H), 0.34 – 0.26 (m, 1H), 0.23 – 0.14 (m, 2H), -0.00 (t, J = 8.8 Hz, 1H). ^{13}C NMR (101 MHz, $CDCl_3$) δ 60.8, 39.1, 34.6, 32.0, 28.6, 28.5, 26.3, 25.1, 23.13, 23.08, 22.8, 19.0, 14.8, 14.4, 14.3, 13.1. HRMS m/z: (APCI+) [M-H]+, calculated for $C_{16}H_{29}O$; 237.2213; found 237.2217.

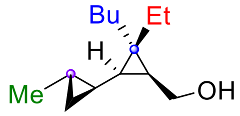

((1R*,1'S*,2S*,2'S*,3S*)-3-butyl-3-ethyl-2'-methyl-[1,1'-bi(cyclopropan)]-2-yl)methanol (3e)
0.5 mmol scale, colorless oil, 92% yield, 97 mg. Flash chromatography: 10 – 25% Et_2O/hexane. ^1H NMR (400 MHz, $CDCl_3$) δ 3.86 – 3.75 (m, 1H), 3.70 (dd, *J* = 11.5, 8.7 Hz, 1H), 1.46 (ddd, *J* = 14.5, 7.3, 3.4 Hz, 2H), 1.24 (tt, *J* = 9.9, 4.9 Hz, 4H), 1.01 (d, *J* = 6.0 Hz, 3H), 0.94 (t, *J* = 7.4 Hz, 3H), 0.88 – 0.79 (m, 4H), 0.60 – 0.53 (m, 1H), 0.35 – 0.28 (m, 1H), 0.27 – 0.20 (m, 1H), 0.20 – 0.11 (m, 1H), 0.07 – 0.02 (m, 1H). ^{13}C NMR (101 MHz, $CDCl_3$) δ 60.4, 38.0, 31.7, 28.9, 28.6, 28.4, 23.0, 19.1, 19.0, 14.4, 14.3, 14.0, 13.6, 10.9. HRMS m/z: (APCI+) [M-H]+, calculated for $C_{14}H_{25}O$; 209.1905; found 209.1907.

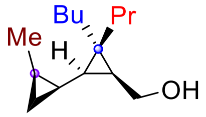

((1R*,1'S*,2S*,2'R*,3S*)-3-butyl-2'-methyl-3-propyl-[1,1'-bi(cyclopropan)]-2-yl)methanol (3f)
0.5 mmol scale, colorless oil, 87% yield, 98 mg. Flash chromatography: 10 – 25% Et_2O/hexane. ^1H NMR (400 MHz, $CDCl_3$) δ 3.91 – 3.79 (m, 1H), 3.77 – 3.67 (m, 1H), 1.48 (dd, *J* = 14.4, 7.2 Hz, 1H), 1.40 (dd, *J* = 13.5, 6.3 Hz, 3H), 1.31 – 1.21 (m, 7H), 1.10 (d, *J* = 6.3 Hz, 3H), 0.90 (dd, *J* = 15.9, 7.5 Hz, 7H), 0.69 (td, *J* = 8.3, 4.0 Hz, 1H), 0.58 – 0.47 (m, 1H), 0.32 (t, *J* = 9.3 Hz, 1H), -0.17 (q, *J* = 4.7 Hz, 1H). ^{13}C NMR (101 MHz, $CDCl_3$) δ 60.6, 39.1, 28.9, 28.8, 28.5, 27.6, 26.1, 23.2, 19.7, 14.9, 14.3, 14.0, 12.5, 10.5, 10.2. HRMS m/z: (APCI+) [M-H]+, calculated for $C_{15}H_{27}O$; 223.2056; found 223.2068.

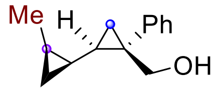

((1R*,1'S*,2R*,2'R*)-2'-methyl-2-phenyl-[1,1'-bi(cyclopropan)]-2-yl)methanol (3g)
0.5 mmol scale, colorless oil, 83% yield, 84 mg, dr of the starting material **1g** was 90:10. Flash chromatography: 10 – 25% Et_2O/hexane. ^1H NMR (400 MHz, $CDCl_3$) δ 7.29 (q, *J* = 7.7 Hz, 4H), 7.18 (t, *J* = 6.9 Hz, 1H), 3.96 (d, *J* = 11.6 Hz, 1H), 3.85 (d, *J* = 11.8 Hz, 1H), 1.18 – 1.10 (m, 4H), 0.96 – 0.74 (m, 4H), 0.65 – 0.57 (m, 1H), -0.01 (q, *J* = 5.0 Hz, 1H).. ^{13}C NMR (101 MHz, $CDCl_3$) δ 144.5, 128.9, 128.4, 126.4, 68.0, 31.8, 23.9, 19.1, 14.9, 13.8, 13.1, 10.5. HRMS m/z: (APCI+) $[M+H]^+$, calculated for $C_{14}H_{17}O$; 201.1274; found 201.1282.

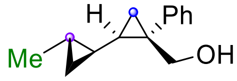

((1R*,1'S*,2R*,2'S*)-2'-methyl-2-phenyl-[1,1'-bi(cyclopropan)]-2-yl)methanol (3h)
0.5 mmol scale, colorless oil, 94% yield, 95 mg. Flash chromatography: 10 – 25% Et_2O/hexane. ^1H NMR (400 MHz, CDCl3) δ 7.31 (q, J = 7.8 Hz, 4H), 7.21 (t, J = 6.7 Hz, 1H), 3.96 (dd, J = 11.5, 6.9 Hz, 1H), 3.88 (dd, J = 11.6, 3.7 Hz, 1H), 1.51 (t, J = 5.6 Hz, 1H), 1.06 (d, J = 5.9 Hz, 3H), 1.01 (dd, J = 11.1, 7.0 Hz, 2H), 0.69 (dt, J = 11.7, 4.8 Hz, 2H), 0.57 (td, J = 9.3, 4.7 Hz, 1H), 0.46 (dt, J = 8.6, 4.5 Hz, 1H), 0.38 (dt, J = 8.5, 4.4 Hz, 1H). ^{13}C NMR (101 MHz, $CDCl_3$) δ 144.3, 128.8, 128.5, 126.5, 67.8, 32.5, 28.3, 18.8, 18.4, 17.5, 13.6, 13.3. HRMS m/z: (APCI+) $[M+H]^+$, calculated for $C_{14}H_{19}O$; 203.1436; found 203.1426.

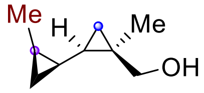

((1R*,1'S*,2R*,2'R*)-2,2'-dimethyl-[1,1'-bi(cyclopropan)]-2-yl)methanol (3i)
0.5 mmol scale, colorless oil, 87% yield, 61 mg. Flash chromatography: 10 – 25% Et_2O/hexane. ^1H NMR (400 MHz, $CDCl_3$) δ 3.66 (d, *J* = 11.3 Hz, 1H), 3.55 (d, *J* = 11.3 Hz, 1H), 1.11 (s, 3H), 0.99 (d, *J* = 6.0 Hz, 3H), 0.56 (dt, *J* = 11.6, 5.7 Hz, 1H), 0.49 (dt, *J* = 8.5, 5.3 Hz, 1H), 0.38 (dd, *J* = 8.3, 4.7 Hz, 1H), 0.36 – 0.29 (m, 2H), 0.29 – 0.20 (m, 2H). ^{13}C NMR (101 MHz, $CDCl_3$) δ 68.2, 27.4, 22.7, 22.3, 18.7, 18.3, 16.9, 13.1, 13.0. HRMS m/z: (APCI+) $[M+H]^+$, calculated for $C_9H_{17}O$; 141.1274; found 141.1274.

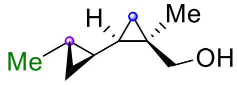

((1R*,1'S*,2R*,2'S*)-2,2'-dimethyl-[1,1'-bi(cyclopropan)]-2-yl)methanol (3j)
0.5 mmol scale, colorless oil, 90% yield, 63 mg. Flash chromatography: 10 – 25% Et_2O/hexane. ^1H NMR (400 MHz, $CDCl_3$) δ 3.72 (d, *J* = 11.4 Hz, 1H), 3.55 (d, *J* = 11.5 Hz, 1H), 1.14 (s, 3H), 1.11 (d, *J* = 6.3 Hz, 3H), 1.07 (s, 1H), 0.85 (ddd, *J* = 11.3, 7.1, 2.1 Hz, 1H), 0.68 (td, *J* = 8.3, 4.2 Hz, 1H), 0.56 (dd, *J* = 7.5, 3.9 Hz, 1H), 0.43 (ddd, *J* = 16.8, 8.4, 5.6 Hz, 1H), 0.36 – 0.27 (m, 2H), -0.14 (dd, *J* = 9.8, 5.1 Hz, 1H). ^{13}C NMR (101 MHz, $CDCl_3$) δ 68.5, 22.8, 22.5, 22.1, 18.9, 15.1, 13.8, 12.7, 10.1. HRMS m/z: (APCI+) $[M+H]^+$, calculated for $C_9H_{17}O$; 141.1274; found 141.1274.

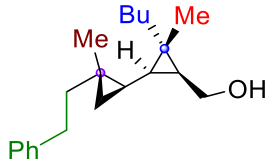

((1R*,1'R*,2S*,2'S*,3S*)-3-butyl-2',3-dimethyl-2'-phenethyl-[1,1'-bi(cyclopropan)]-2-yl)methanol (3k)
0.5 mmol scale, colorless oil, 83% yield, 125 mg. Flash chromatography: 10 – 25% Et_2O/hexane. ^1H NMR (400 MHz, $CDCl_3$) δ 7.15 (dd, *J* = 10.0, 5.1 Hz, 2H), 7.05 (dd, *J* = 7.2, 5.4 Hz, 3H), 3.64 (qd, *J* = 11.5, 7.9 Hz, 2H), 2.65 – 2.47 (m, 2H), 1.40 (ddd, *J* = 10.7, 6.2, 1.5 Hz, 2H), 1.30 – 1.11 (m, 7H), 1.05 (s, 3H), 0.94 (s, 3H), 0.78 (t, *J* = 7.1

Hz, 4H), 0.44 (dd, *J* = 8.0, 4.2 Hz, 1H), 0.35 – 0.12 (m, 2H), 0.00 (t, *J* = 4.4 Hz, 1H). ^{13}C NMR (101 MHz, $CDCl_3$) δ 142.9, 128.41, 128.37, 125.7, 60.6, 44.0, 42.8, 33.3, 28.9, 28.5, 27.4, 23.3, 23.1, 20.3, 20.2, 19.1, 18.2, 14.3, 13.2. HRMS m/z: (APCI+) $[M+H]^+$, calculated for $C_{21}H_{33}O$; 301.2526; found 301.2543.

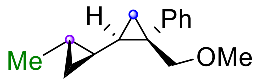

(1R*,1'S*,2R*,2'S*)-2-(methoxymethyl)-2'-methyl-2-phenyl-1,1'-bi(cyclopropane) (3l)
0.5 mmol scale, colorless oil, 84% yield, 91 mg. Flash chromatography: 5 – 10% Et_2O/hexane. ^{1}H NMR (400 MHz, $CDCl_3$) δ 7.23 – 7.17 (m, 4H), 7.09 (t, *J* = 6.4 Hz, 1H), 3.62 (s, 2H), 3.24 (s, 3H), 1.09 – 1.02 (m, 1H), 0.97 (d, *J* = 5.4 Hz, 3H), 0.91 (dd, *J* = 8.6, 4.9 Hz, 1H), 0.61 (dd, *J* = 11.6, 6.0 Hz, 1H), 0.52 (dtd, *J* = 12.7, 8.4, 4.5 Hz, 2H), 0.40 – 0.32 (m, 1H), 0.28 (dt, *J* = 9.4, 4.7 Hz, 1H). ^{13}C NMR (101 MHz, $CDCl_3$) δ 145.2, 128.23, 128.18, 128.1, 126.0, 58.9, 30.0, 28.5, 18.8, 18.3, 17.4, 13.2, 13.1. HRMS m/z: (APCI+) $[M+H]^+$, calculated for $C_{15}H_{21}O$; 217.1587; found 217.1579.

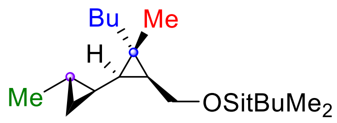

tert-butyl(((1R*,1'S*,2S*,2'S*,3S*)-3-butyl-2',3-dimethyl-[1,1'-bi(cyclopropan)]-2-yl)methoxy)dimethylsilane (3m)
0.5 mmol scale, colorless oil, 95% yield, 148 mg. Flash chromatography: 0 – 10% Et_2O/hexane. ^{1}H NMR (400 MHz, $CDCl_3$) δ 3.73 – 3.68 (dd, 1H), 3.64 – 3.59 (dd, 1H), 1.37 – 1.16 (m, 5H), 1.07 – 1.04 (t, 2H), 0.96 – 0.95 (m, 6H), 0.84 – 0.80 (m, 14H) 0.69 – 0.63 (m, 1H), 0.47 – 0.43 (m, 1H), 0.29-0.25 (m, 1H) 0.17-0.14 (m, 1H), 0.001 (s, 9H). ^{13}C NMR (101 MHz, $CDCl_3$) δ 127.2, 126.2, 60.4, 42.6, 30.8, 29.5, 28.7, 26.0, 24.6, 22.8, 18.4, 14.3, 12.8, -5.1, -5.2. HRMS m/z: (APCI+) $[M+H]^+$, calculated for $C_{19}H_{39}OSi$; 311.2765; found 311.2762.

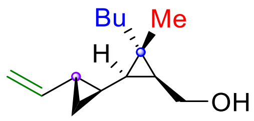

((1R*,1'S*,2S*,2'R*,3S*)-3-butyl-3-methyl-2'-vinyl-[1,1'-bi(cyclopropan)]-2-yl)methanol (3n)
0.5 mmol scale, colorless oil, 81% yield, 84 mg, *dr = 2:1* in mixture with 10% starting material, accounted yield determination. Flash chromatography: 10 – 25% Et_2O/hexane. ^{1}H NMR (400 MHz, CDCl3) δ 5.48 (m, 1H), 5.19 – 4.76 (m, 2H), 3.82 – 3.63 (m, 2H), 1.55 (ddd, J = 17.3, 8.6, 5.5 Hz, 1H), 1.37 – 1.19 (m, 6H), 1.14 (ddd, J = 11.9, 7.9, 3.0 Hz, 1H), 1.06 – 1.00 (m, 3H), 0.94 (ddd, J = 15.8, 7.9, 4.1 Hz, 1H), 0.91 – 0.81 (m, 4H), 0.79 – 0.71 (m, 1H), 0.69 – 0.61 (m, 1H), 0.53 – 0.43 (m, 1H), 0.36 (ddd, J = 15.3, 8.8, 5.6 Hz, 1H), 0.21 – 0.12 (m, 1H). ^{13}C NMR (101 MHz, CDCl3) δ 141.4, 139.0, 114.1, 111.5, 60.4, 60.2, 42.6, 42.4, 30.7, 28.9, 28.8, 28.60, 28.57, 26.7, 23.4, 23.3, 23.2, 22.9, 22.8, 20.2, 16.1, 14.5, 14.2, 13.8, 13.6, 12.9, 12.8. HRMS m/z: (APCI+) $[M-H]^+$, calculated for $C_{14}H_{25}O$; 209.1905; found 209.1909.

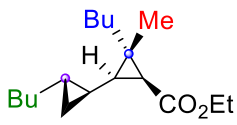

Ethyl (1R*,1'S*,2S*,2'S*,3S*)-2',3-dibutyl-3-methyl-[1,1'-bi(cyclopropane)]-2-carboxylate (3o)
0.5 mmol scale, colorless oil, 81% yield, 114 mg. Flash chromatography: 5 – 15% Et_2O/hexane. ^{1}H NMR (400 MHz, $CDCl_3$) δ 4.17 – 4.05 (m, 2H), 1.31 (ddd, *J* = 20.8, 11.0, 6.9 Hz, 15H), 1.19 – 1.09 (m, 2H), 0.88 (t, *J* = 7.0 Hz, 6H), 0.62 – 0.51 (m, 1H), 0.48 (t, *J* = 9.0 Hz, 1H), 0.34 – 0.24 (m, 2H). ^{13}C NMR (101 MHz, $CDCl_3$) δ 172.0, 59.7, 42.7, 38.2, 33.8, 31.9, 30.2, 28.8, 28.7, 22.8, 22.6, 19.4, 14.5, 14.2, 12.6, 12.3, 12.1. HRMS m/z: (APCI+) $[M+H]^+$, calculated for $C_{18}H_{33}O_2$; 281.2475; found 281.2450.

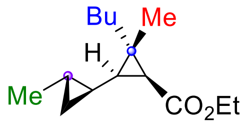

ethyl (1R*,1'S*,2S*,2'S*,3S*)-3-butyl-2',3-dimethyl-[1,1'-bi(cyclopropane)]-2-carboxylate (3p)
0.5 mmol scale, colorless oil, 90% yield, 107 mg. Flash chromatography: 5 – 15% Et_2O/hexane. ^1H NMR (400 MHz, $CDCl_3$) δ 4.10 (qd, J = 7.1, 2.5 Hz, 2H), 1.37 (d, J = 8.8 Hz, 1H), 1.27 (s, 3H), 1.24 (d, J = 7.1 Hz, 6H), 1.03 (d, J = 6.0 Hz, 3H), 0.86 (dd, J = 12.1, 5.1 Hz, 4H), 0.60 – 0.42 (m, 2H), 0.28 (ddt, J = 12.9, 9.3, 4.6 Hz, 2H). ^{13}C NMR (101 MHz, $CDCl_3$) δ 172.0, 59.7, 42.6, 38.0, 30.2, 28.79 28.72, 22.8, 18.8, 14.5, 14.2, 13.7, 13.6, 13.4, 12.2. HRMS m/z: (APCI+) $[M+H]^+$, calculated for $C_{15}H_{27}O_2$; 239.2006; found 239.2001.

Procedure for the epoxidation of alkenyl cyclopropyl carbinols 1

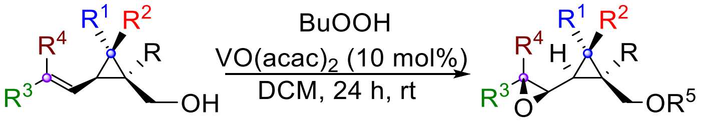

In a flamed-dried three-neck flask under argon atmosphere, vanadyl acetylacetonate ($VO(acac)_2$, 10 mol%) was dissolved in dry DCM (0.1 M, 10 mL of DCM/ mmol of **1**). The solution was cooled to 0 °C using an ice bath. A solution of tertbutyl hydroperoxide (*t*BuOOH, 5.5 M in decane, 5 equiv.) was added dropwise to the solution, resulting in the formation of a brown solution. The solution was stirred for 5 minutes and then a solution of alkenyl cyclopropane **1** in DCM (1 equiv., 0.5 mmol, 1 M, 1mL of DCM/ mmol of **1**) was added dropwise. The ice bath was removed, and the reaction was stirred for 24 h. Upon completion, as monitored by GC analysis of aliquots, the reaction was diluted with DCM and washed with an aqueous saturated solution of sodium sulfite. The layers were separated, and the aqueous phase was extracted three times with Et_2O. The combined organic phases were dried over anhydrous Na_2SO_4 and concentrated under reduced pressure. Purification by column chromatography (gradient eluent: 10 – 25% Et2O/hexane) afforded the desired pure compound.

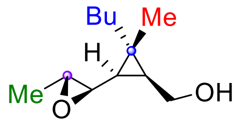

((1S*,2R*,3R*)-2-butyl-2-methyl-3-((2R*,3R*)-3-methyloxiran-2-yl)cyclopropyl)methanol (4a)
1 mmol scale, colorless oil, 84% yield, 167 mg. Flash chromatography: 10 – 25% Et_2O/hexane. ^1H NMR (400 MHz, $CDCl_3$) δ 3.93 (dd, J = 8.6, 4.4 Hz, 1H), 3.74 (d, J = 8.6 Hz, 1H), 3.64 – 3.54 (m, 1H), 3.50 (d, J = 7.6 Hz, 1H), 1.46 – 1.41 (m, 1H), 1.33 – 1.26 (m, 4H), 1.19 – 1.15 (m, 5H), 0.99 (s, 3H), 0.87 (t, J = 7.1 Hz, 4H). ^{13}C NMR (101 MHz, $CDCl_3$) δ 83.7, 69.4, 67.5, 40.0, 30.8, 29.2, 28.9, 23.1, 23.0, 18.6, 14.2, 10.2. HRMS m/z: (APCI+) $[M+H]^+$, calculated for $C_{12}H_{23}O_2$; 199.1693; found 199.1692.

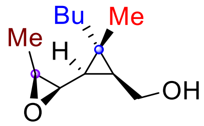

((1S*,2R*,3R*)-2-butyl-2-methyl-3-((2R*,3S*)-3-methyloxiran-2-yl)cyclopropyl)methanol (4b)

1 mmol scale, colorless oil, 91% yield, 180 mg. Flash chromatography: 10 – 25% Et_2O/hexane. ^1H NMR (400 MHz, $CDCl_3$) δ 3.94 (dd, *J* = 8.4, 4.5 Hz, 1H), 3.72 (dt, *J* = 14.1, 4.1 Hz, 1H), 2.35 (s, 1H), 1.46 (dd, *J* = 7.6, 4.5 Hz, 1H), 1.34 – 1.19 (m, 9H), 1.18 – 1.12 (m, 5H), 0.94 (s, 3H), 0.85 (t, *J* = 7.1 Hz, 3H). ^{13}C NMR (101 MHz, $CDCl_3$) δ 83.0, 70.6, 68.9, 39.9, 30.6, 29.6, 28.9, 23.2, 23.0, 18.5, 14.2, 10.0. HRMS m/z: (APCI+) $[M+H]^+$, calculated for $C_{12}H_{23}O_2$; 199.1693; found 199.1705.

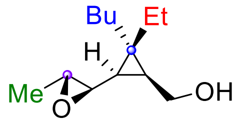

((1S*,2R*,3R*)-2-butyl-2-ethyl-3-((2R*,3R*)-3-methyloxiran-2-yl)cyclopropyl)methanol (4c)
0.5 mmol scale, colorless oil, 75% yield, 80 mg. Flash chromatography: 10 – 25% Et_2O/hexane. ^1H NMR (400 MHz, $CDCl_3$) δ 3.89 (dd, *J* = 8.6, 4.6 Hz, 1H), 3.69 (d, *J* = 8.6 Hz, 1H), 3.57 – 3.49 (m, 1H), 3.46 (d, *J* = 7.5 Hz, 1H), 1.43 (dd, *J* = 7.3, 4.5 Hz, 1H), 1.37 (d, *J* = 7.5 Hz, 2H), 1.25 – 1.17 (m, 6H), 1.14 – 1.11 (m, 3H), 0.87 (t, *J* = 7.5 Hz, 3H), 0.85 – 0.78 (m, 5H). ^{13}C NMR (101 MHz, $CDCl_3$) δ 83.7, 69.5, 67.7, 35.4, 31.6, 30.0, 28.4, 28.1, 23.0, 18.5, 15.8, 14.2, 10.9. HRMS m/z: (APCI+) $[M-H]^+$, calculated for $C_{13}H_{23}O_2$; 211.1698; found 211.1700.

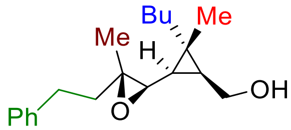

((1S*,2R*,3R*)-2-butyl-2-methyl-3-((2R*,3R*)-3-methyl-3-phenethyloxiran-2-yl)cyclopropyl)methanol (4d)
0.5 mmol scale, colorless oil, 85% yield, 129 mg. Flash chromatography: 10 – 25% Et_2O/hexane. ^1H NMR (400 MHz, $CDCl_3$) δ 7.23 – 7.18 (m, 2H), 7.17 – 7.09 (m, 3H), 3.93 (dd, *J* = 8.4, 4.5 Hz, 1H), 3.70 (d, *J* = 8.4 Hz, 1H), 3.59 (s, 1H), 2.70 (ddd, *J* = 9.2, 6.6, 3.7 Hz, 2H), 1.78 – 1.68 (m, 2H), 1.44 (dd, *J* = 7.7, 4.5 Hz, 1H), 1.28 – 1.17 (m, 9H), 1.12 (s, 3H), 0.90 (s, 3H), 0.81 (t, *J* = 7.1 Hz, 4H). ^{13}C NMR (101 MHz, $CDCl_3$) δ 142.9, 128.53, 128.50, 125.8, 85.6, 75.0, 69.0, 40.5, 40.0, 30.6, 30.1, 29.8, 29.0, 23.4, 23.0, 22.4, 14.3, 10.0. HRMS m/z: (APCI+) $[M+H]^+$, calculated for $C_{20}H_{31}O_2$; 303.2319; found 303.2315.

References

(1) Liao, L. A.; Fox, J. M. A. Copper-Catalyzed Method for the Facially Selective Addition of Grignard Reagents to Cyclopropenes. *J. Am. Chem. Soc.* **2002**, *124*, 14322–14323.

(2) Liu, X.; Fox, J. M. Enantioselective, Facially Selective Carbomagnesation of Cyclopropenes. *J. Am. Chem. Soc.* **2006**, *128*, 5600–5601.

(3) Müller, D.; Alexakis, A. Formation of Quaternary Stereogenic Centers by Copper-Catalyzed Asymmetric Conjugate Addition Reactions of Alkenylaluminums to Trisubstituted Enones. *Chem. - A Eur. J.* **2013**, *19*, 15226–15239.

(4) Wipf, P.; Lim, S. Rapid Carboalumination of Alkynes in the Presence of Water. *Angew. Chem. Int. Ed. Eng.* **1993**, *32*, 1068–1071.

(5) Cohen, A.; Chagneau, J.; Marek, I. Stereoselective Preparation of Distant Stereocenters (1,5) within Acyclic Molecules. *ACS Catal.* **2020**, *10*, 7154–7161.

(6) Bruffaerts, J.; Pierrot, D.; Marek, I. Efficient and Stereodivergent Synthesis of Unsaturated Acyclic Fragments Bearing Contiguous Stereogenic Elements. *Nat. Chem.* **2018**, *10*, 1164–1170.

(7) Pierrot, D.; Marek, I. Stereospecific Reactions Leading to Allylboronic Esters Within Acyclic Systems Bearing Distant Stereocenters. *Angew. Chem. Int. Ed.* **2020**, *59*, 20434–20438.

NMR spectra for new compounds

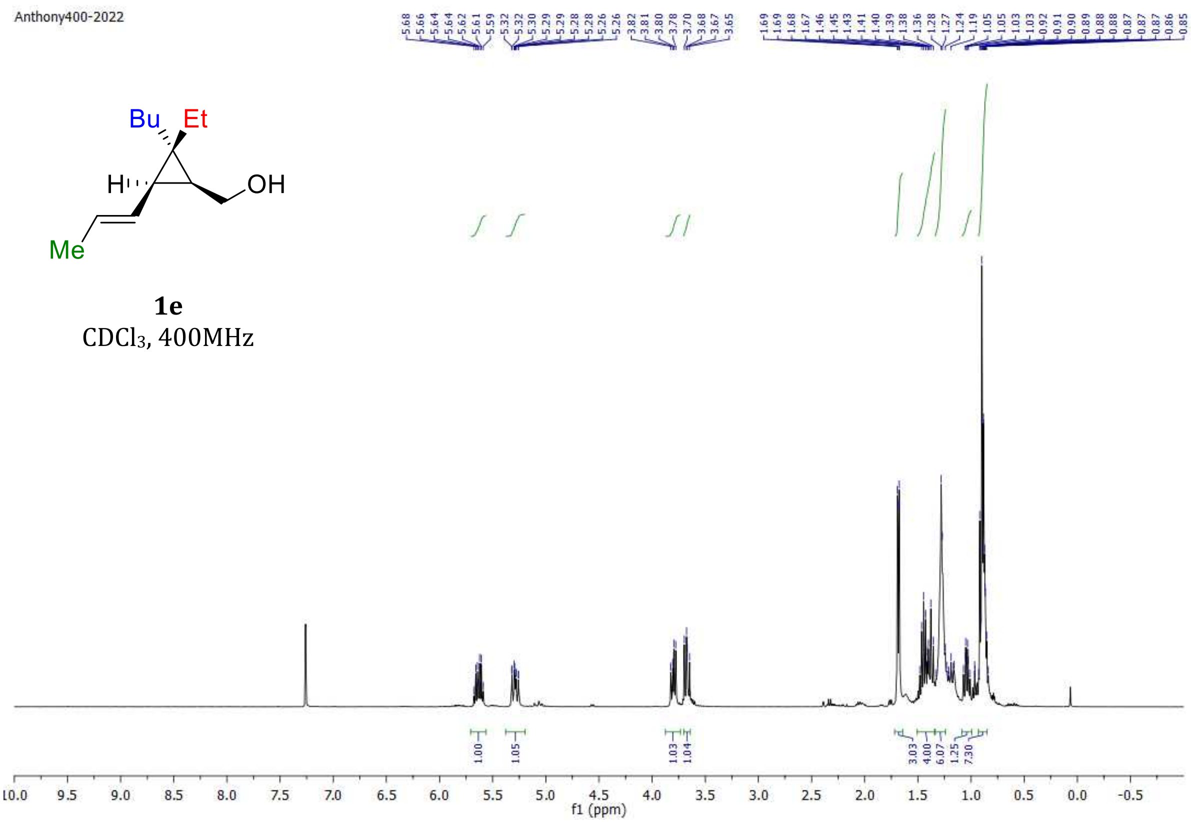

1e
$CDCl_3$, 400MHz

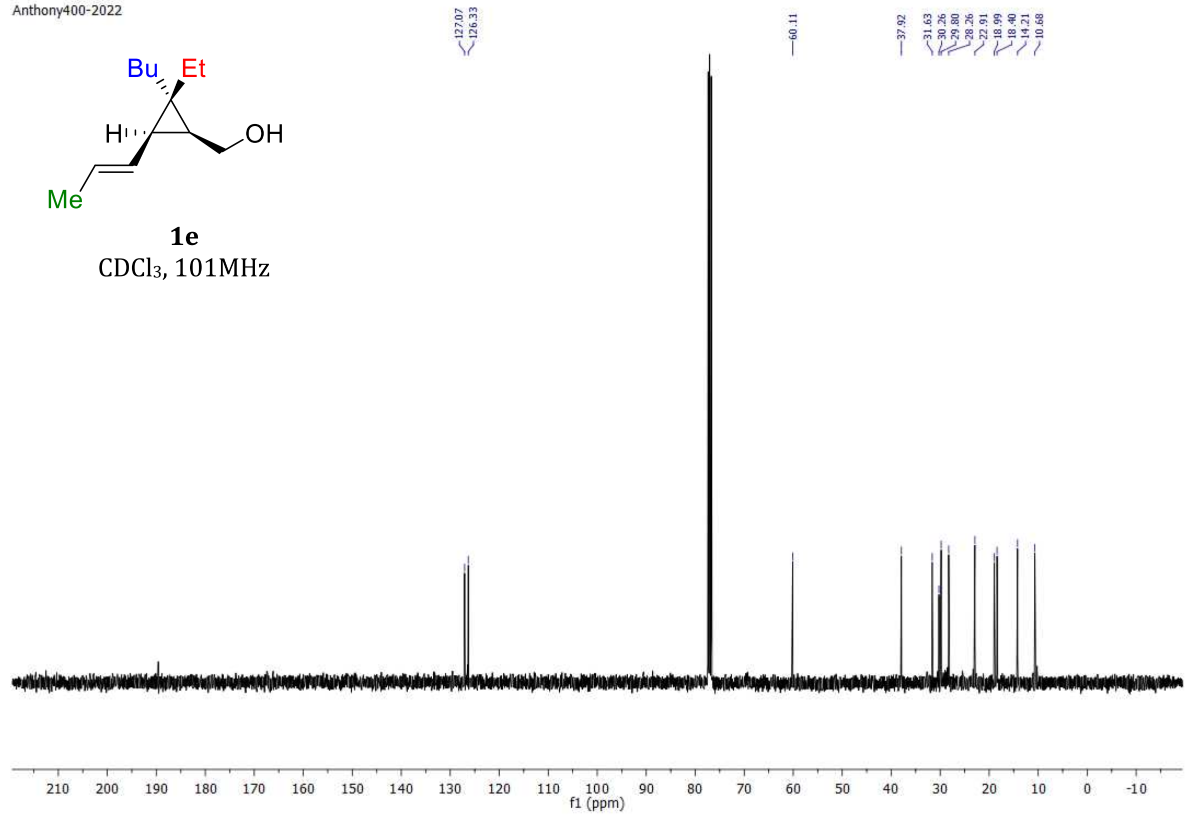

1e
$CDCl_3$, 101MHz

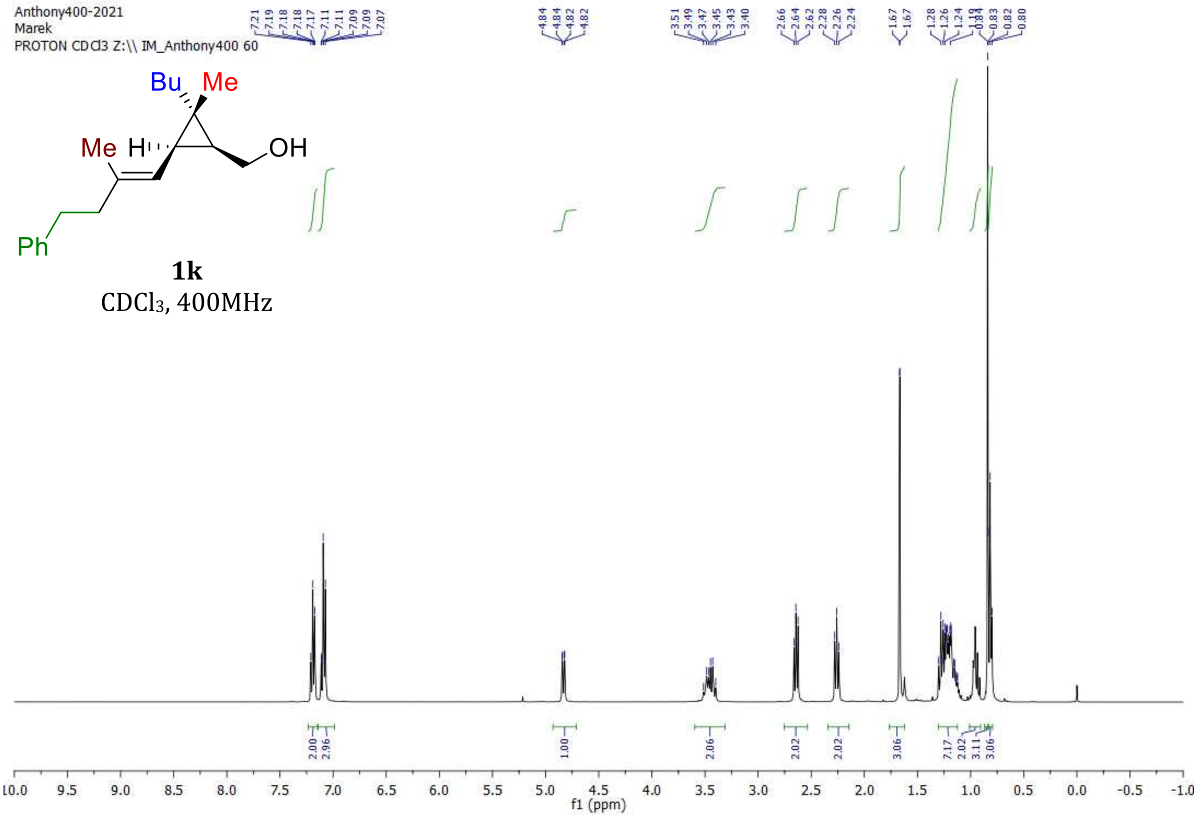
Anthony400-2021
Marek
PROTON CDCl3 Z:\\ IM_Anthony400 60
7.21 7.19 7.18 7.18 7.17 7.11 7.11 7.11 7.09 7.09 7.07
4.84 4.84 4.82 4.82
3.51 3.49 3.47 3.45 3.45 3.43 3.40
2.66 2.64 2.62 2.28 2.26 2.24
1.67 1.67
1.28 1.26 1.24 1.19 0.84 0.83 0.82 0.80
Bu
Me
Me
H
OH
Ph
1k
CDCl3, 400MHz
2.00 2.96 1.00 2.06 2.02 2.02 3.06 7.17 2.02 3.11 3.06
f1 (ppm)

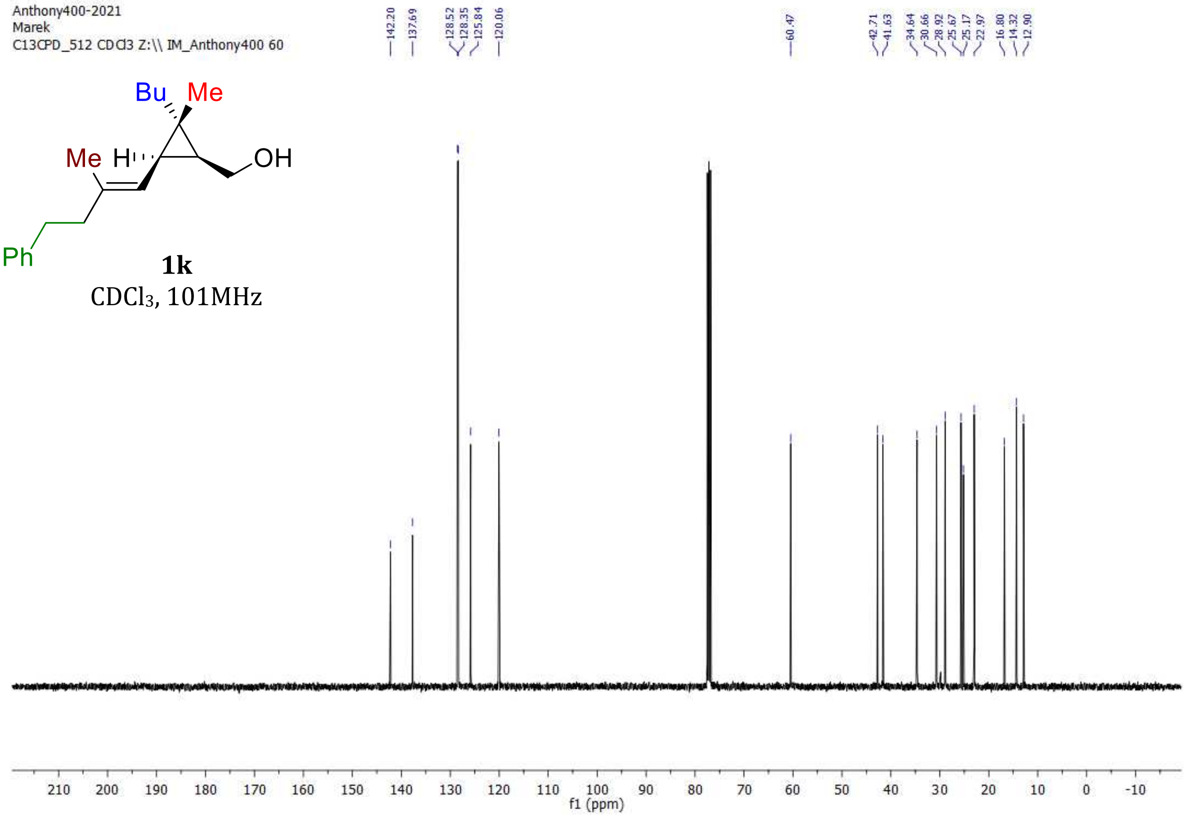
Anthony400-2021
Marek
C13CPD_512 CDCl3 Z:\\ IM_Anthony400 60
142.20 137.69 128.52 128.35 125.84 120.06
60.47
42.71 41.63 34.64 30.66 28.92 25.67 25.17 22.97 16.80 14.32 12.90
Bu
Me
Me
H
OH
Ph
1k
CDCl3, 101MHz
f1 (ppm)

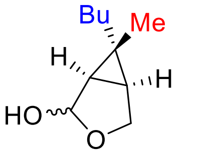

12
$CDCl_3$, 400MHz

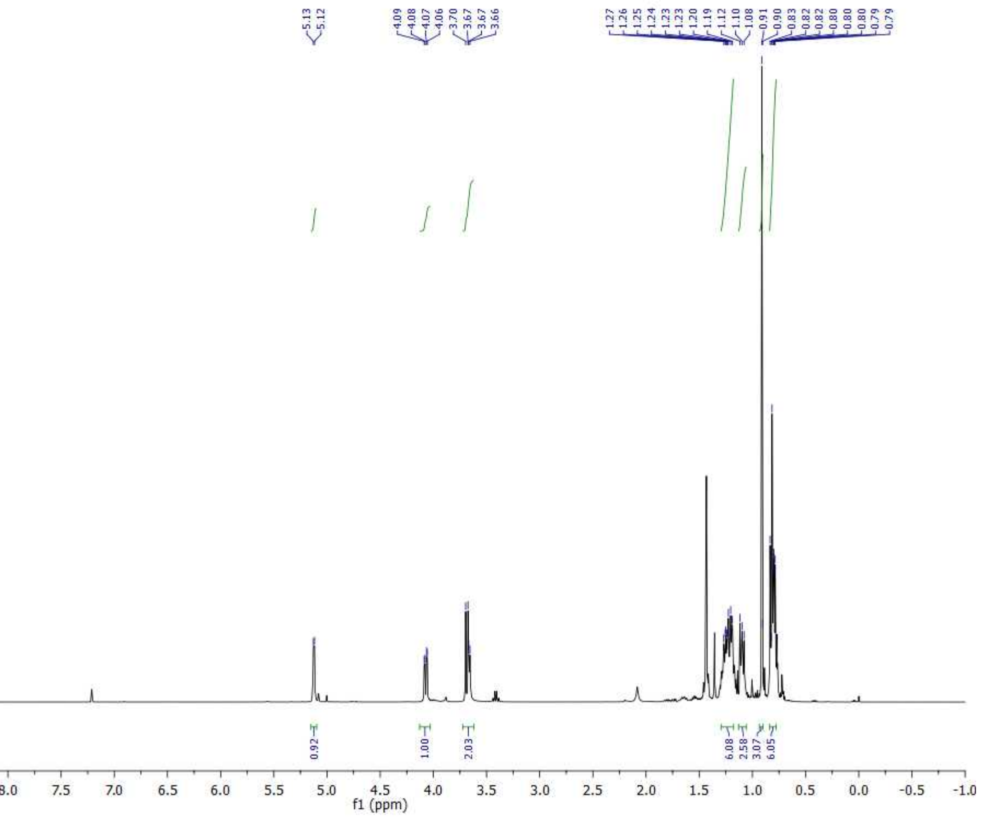

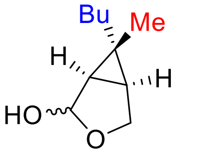

12
$CDCl_3$, 101MHz

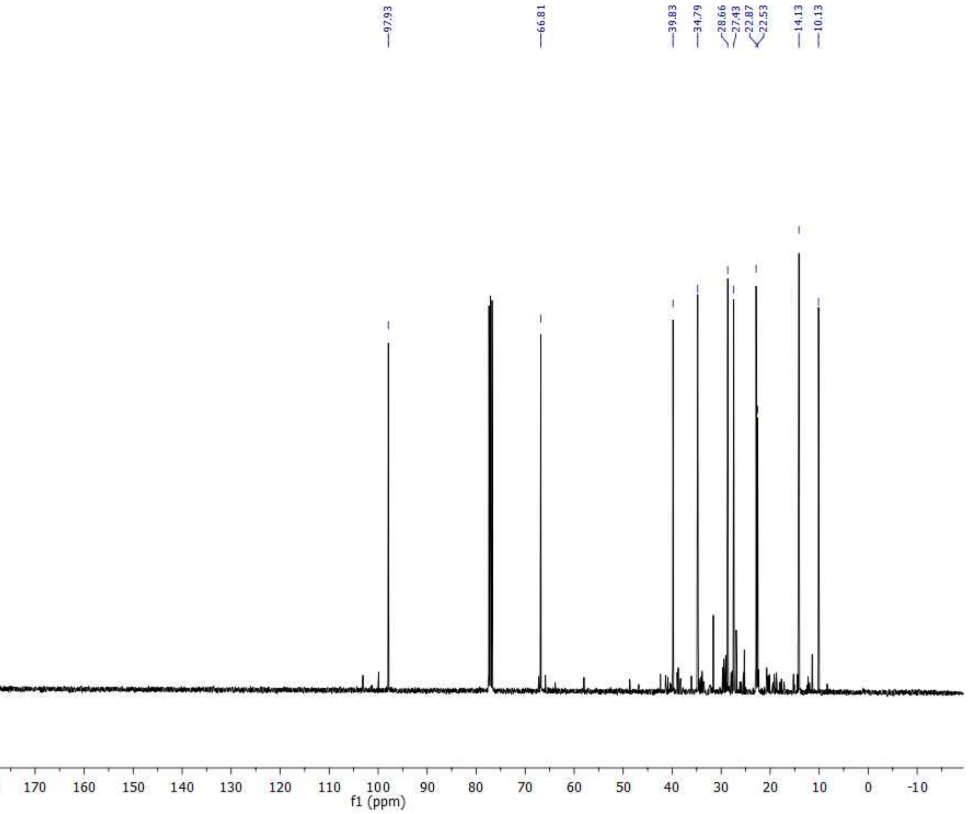

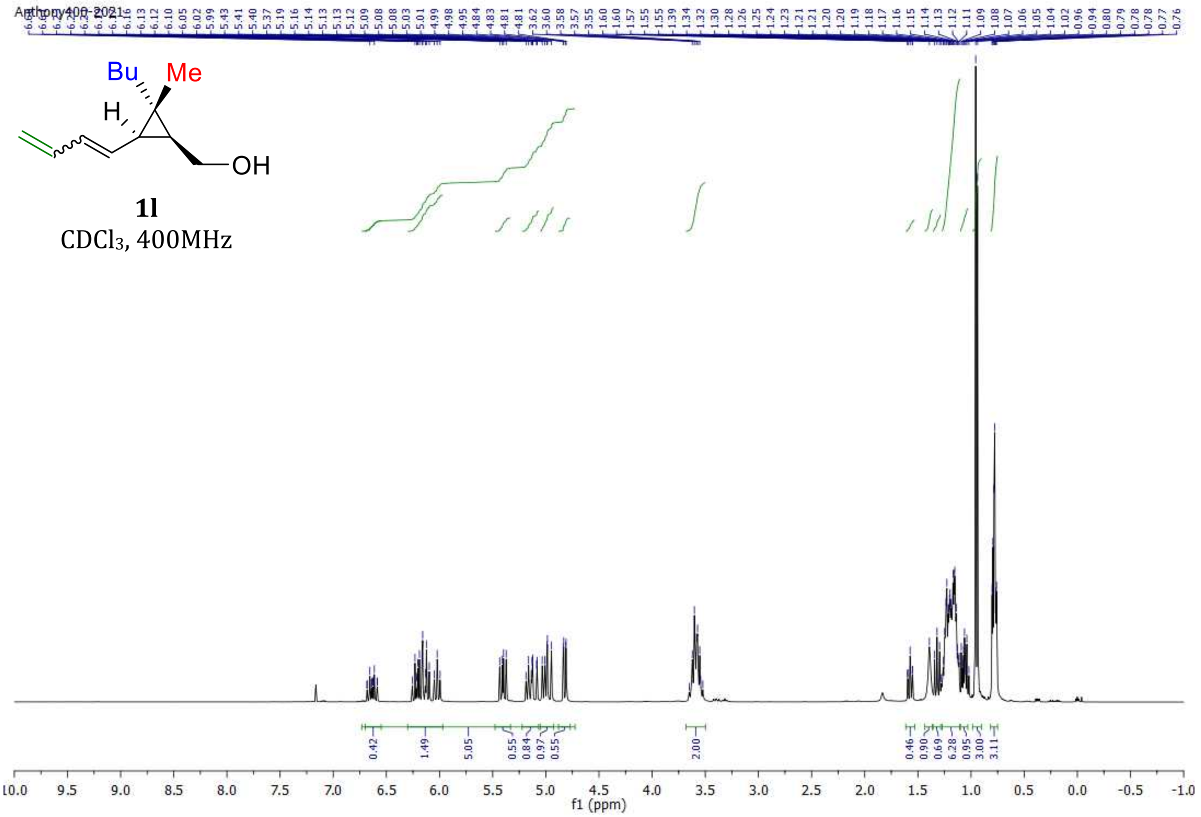

1l
$CDCl_3$, 400MHz

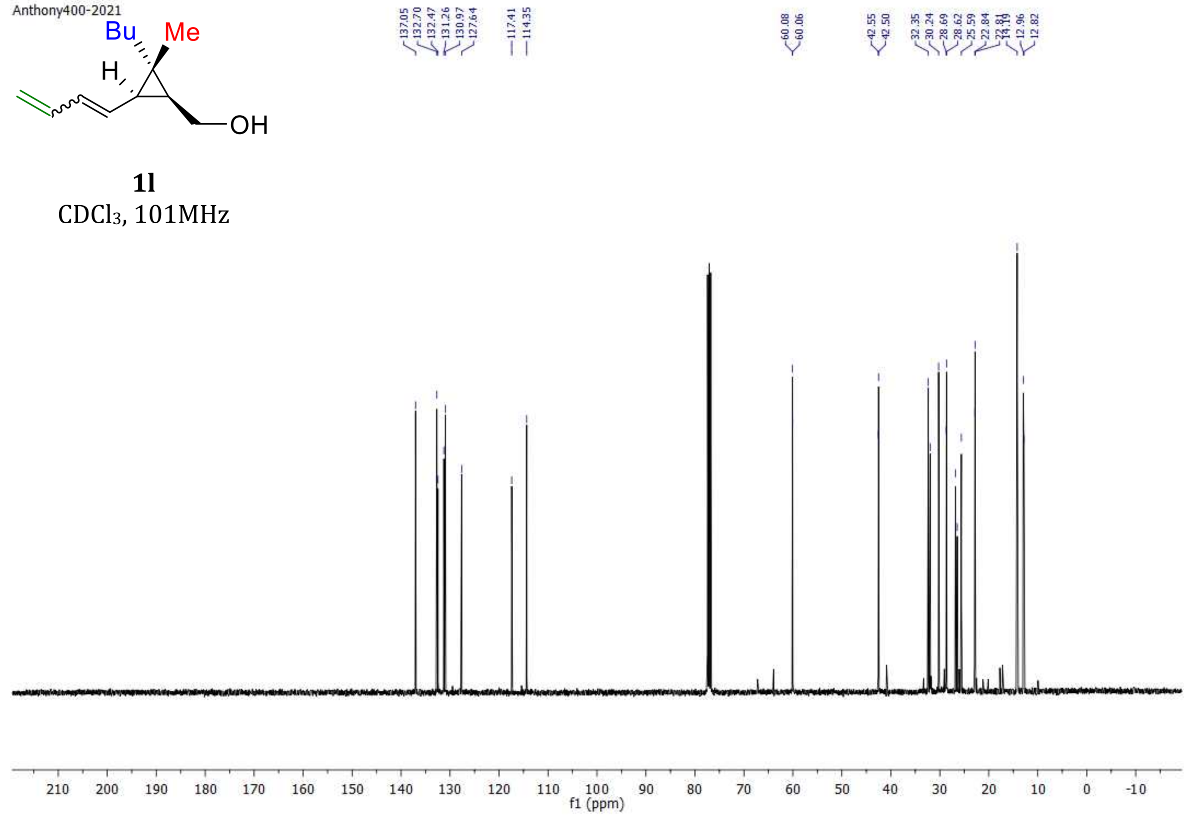

1l
$CDCl_3$, 101MHz

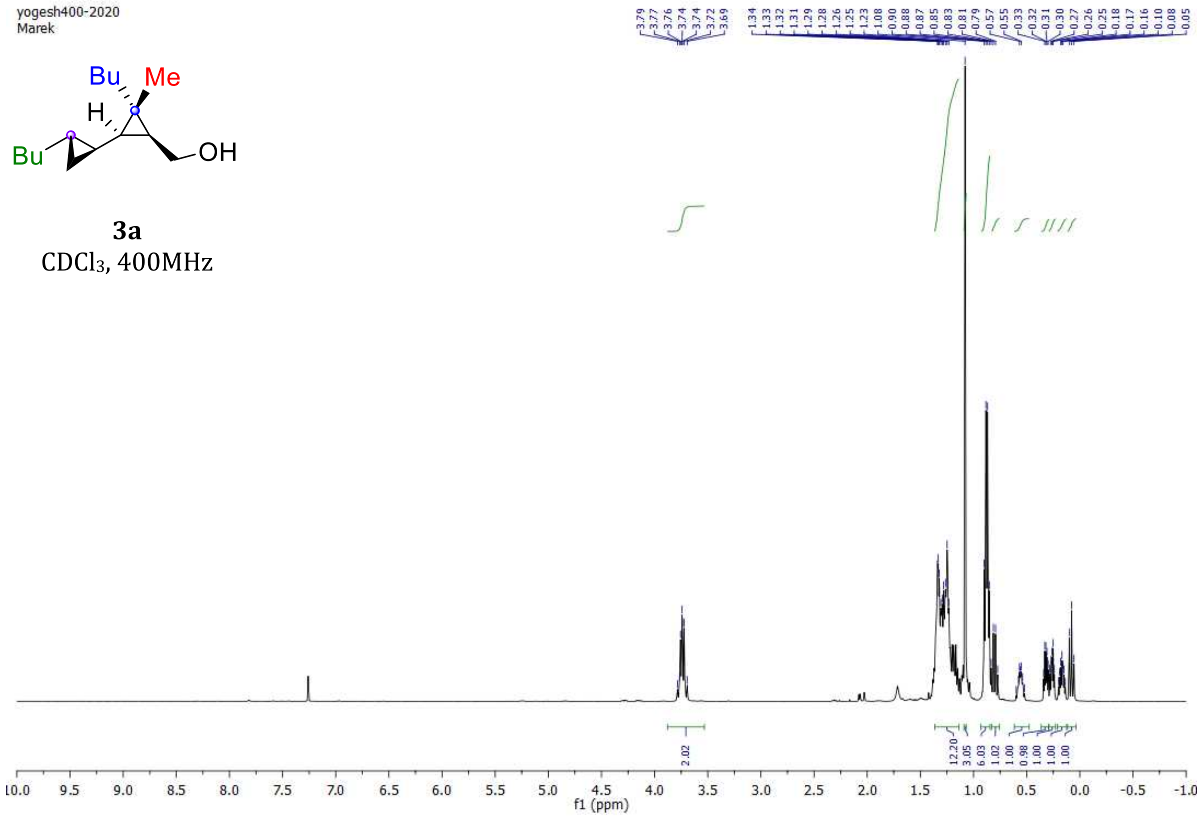
yogesh400-2020
Marek
Bu
Me
H
Bu
OH
3a
CDCl3, 400MHz
f1 (ppm)

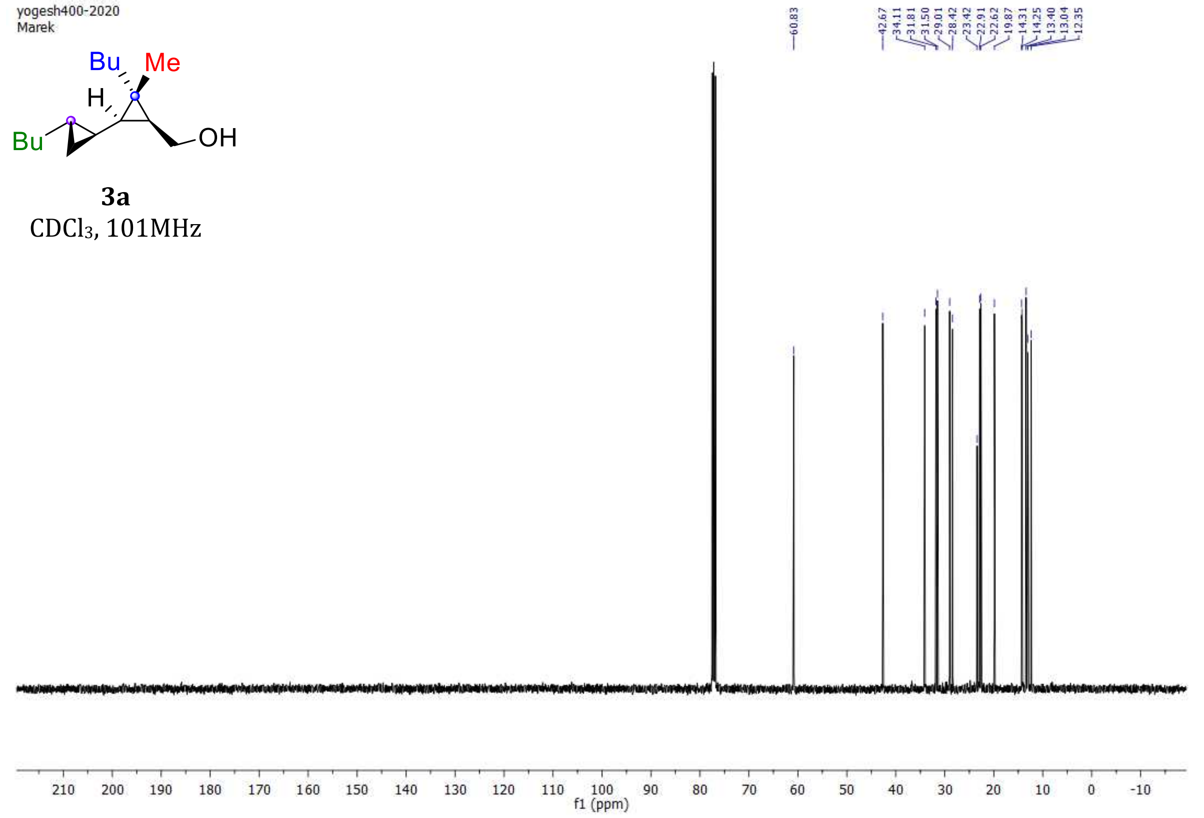
yogesh400-2020
Marek
Bu
Me
H
Bu
OH
3a
CDCl3, 101MHz
f1 (ppm)

yogesh400-2020
Marek

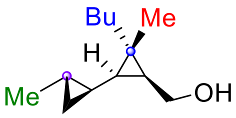

3b
$CDCl_3$, 400MHz

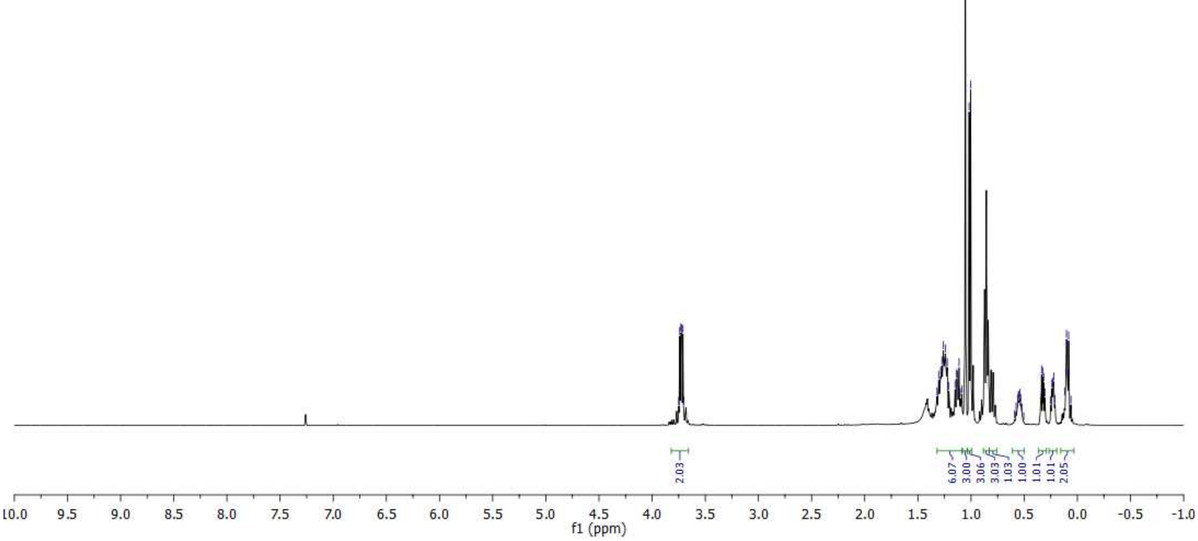

yogesh400-2020
Marek

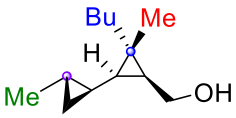

3b
$CDCl_3$, 101MHz

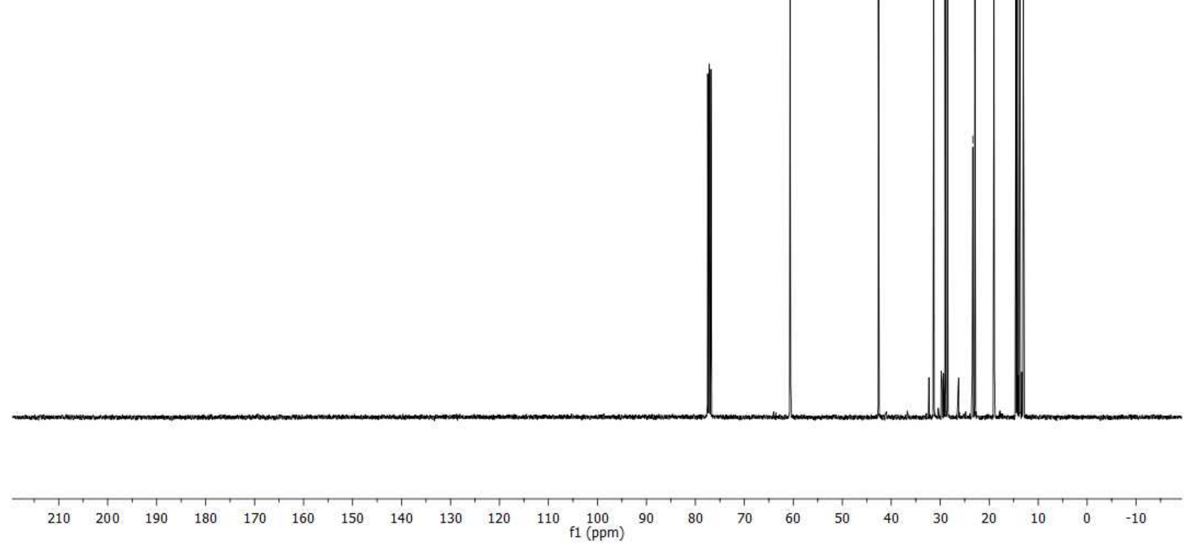

Anthony400-2022

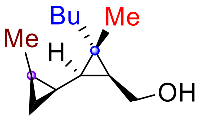

3c

$CDCl_3$, 400MHz

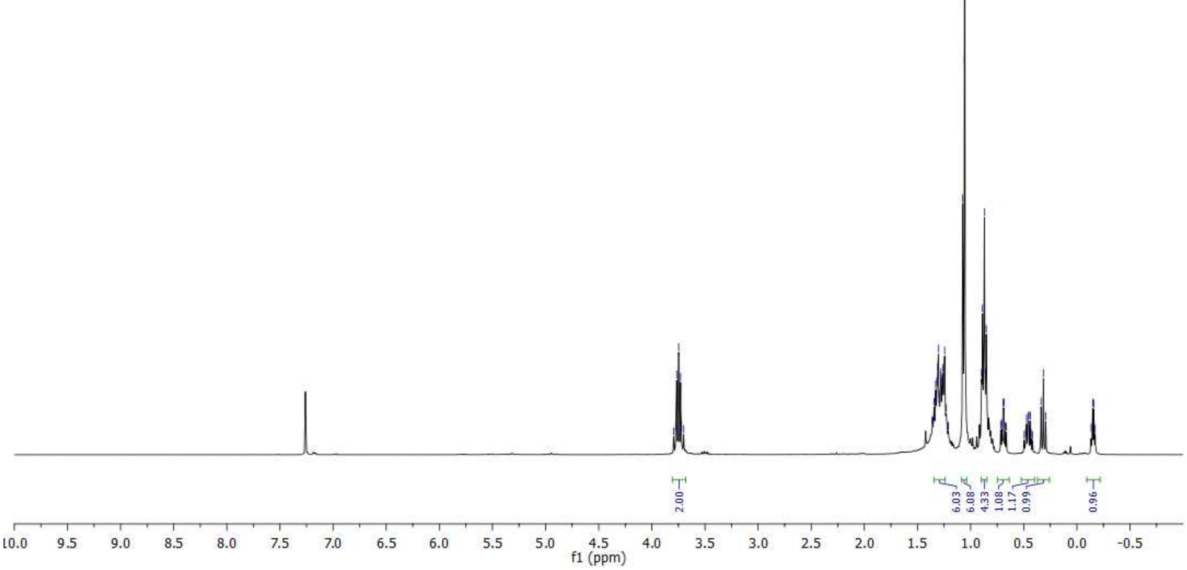

Anthony400-2022

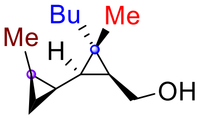

3c

$CDCl_3$, 101MHz

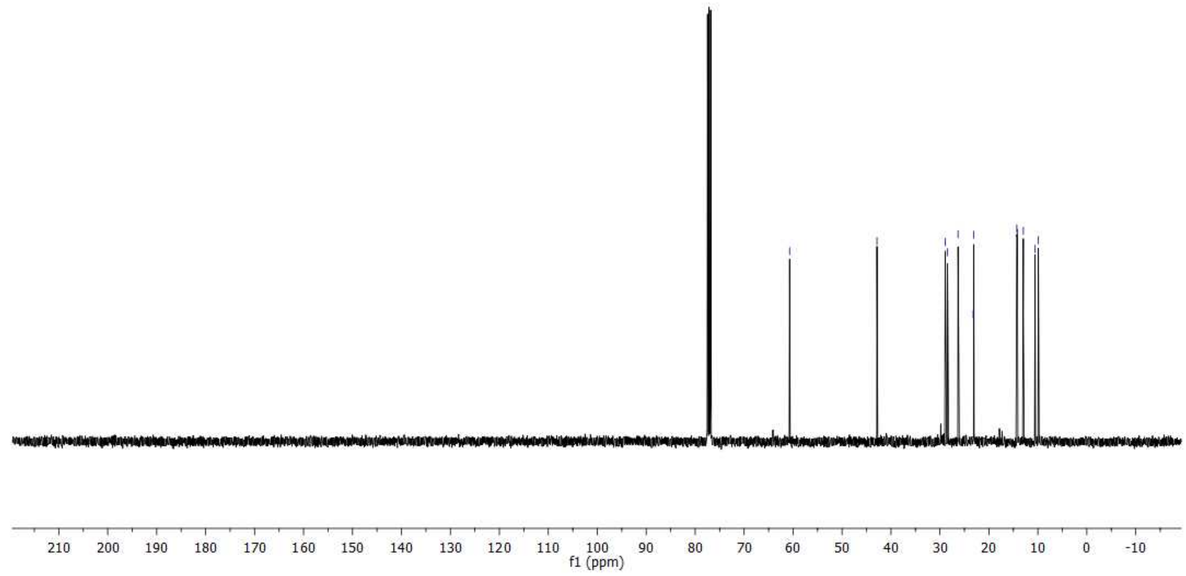

Overlay of 3b and 3c, demonstrating diastereomeric purity

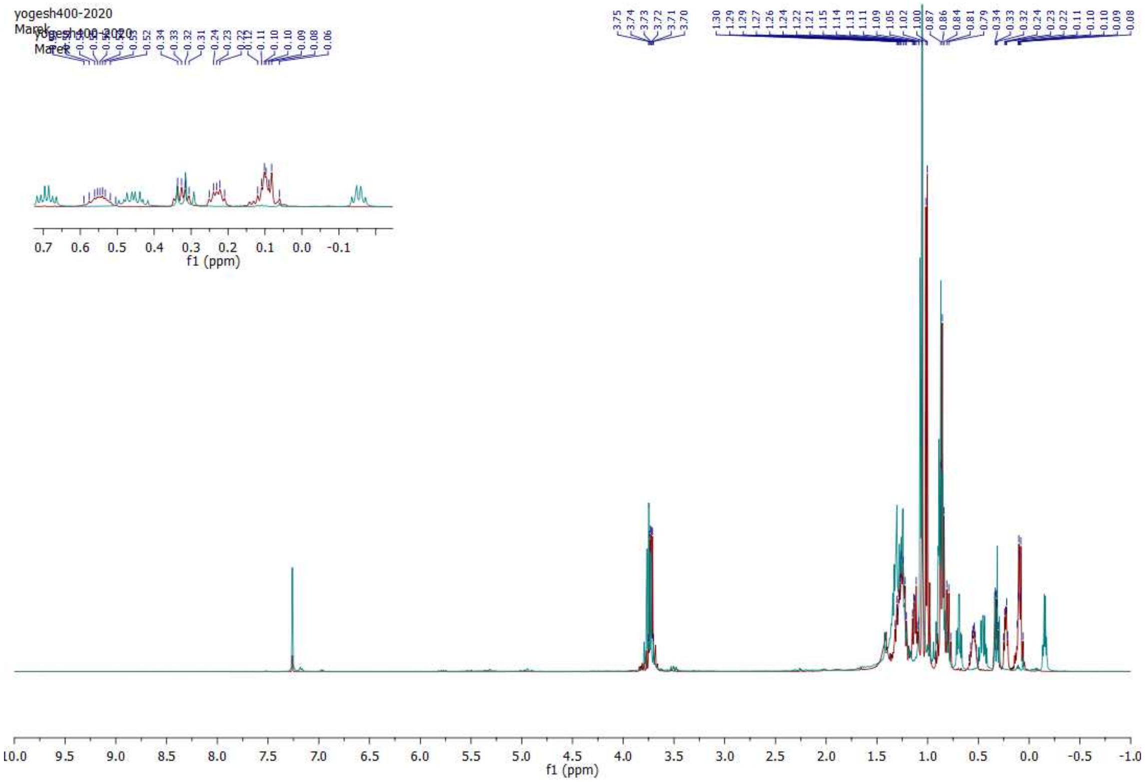

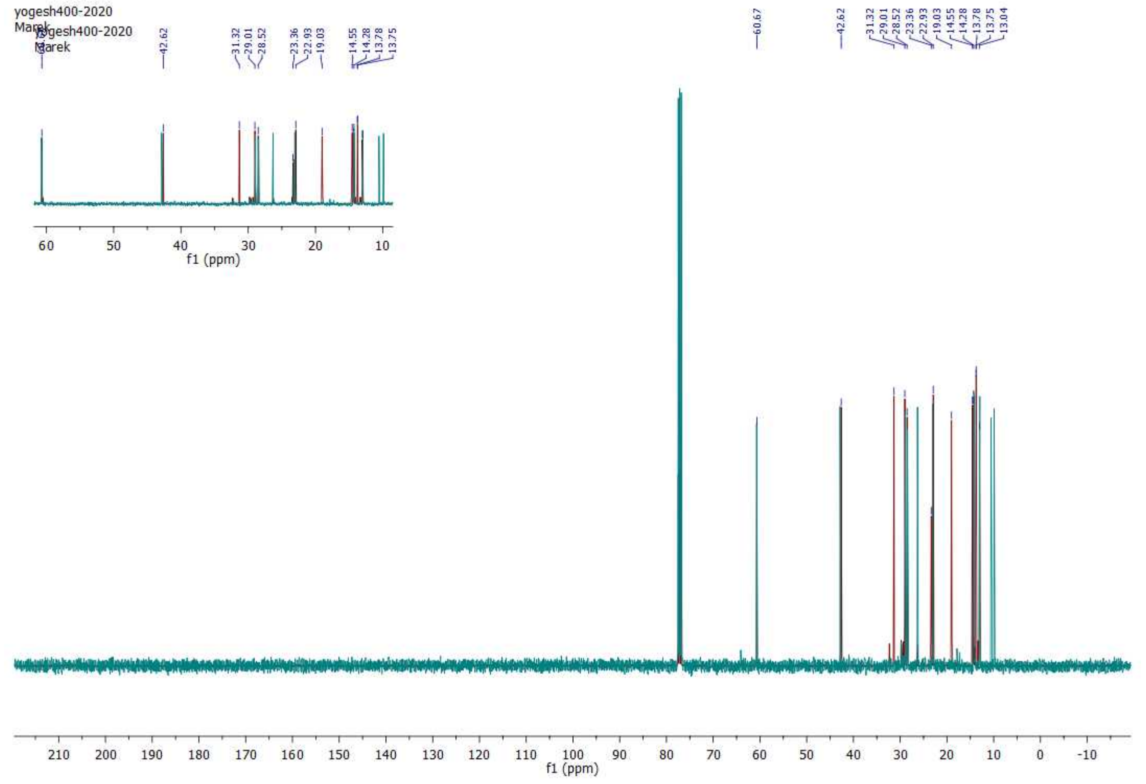

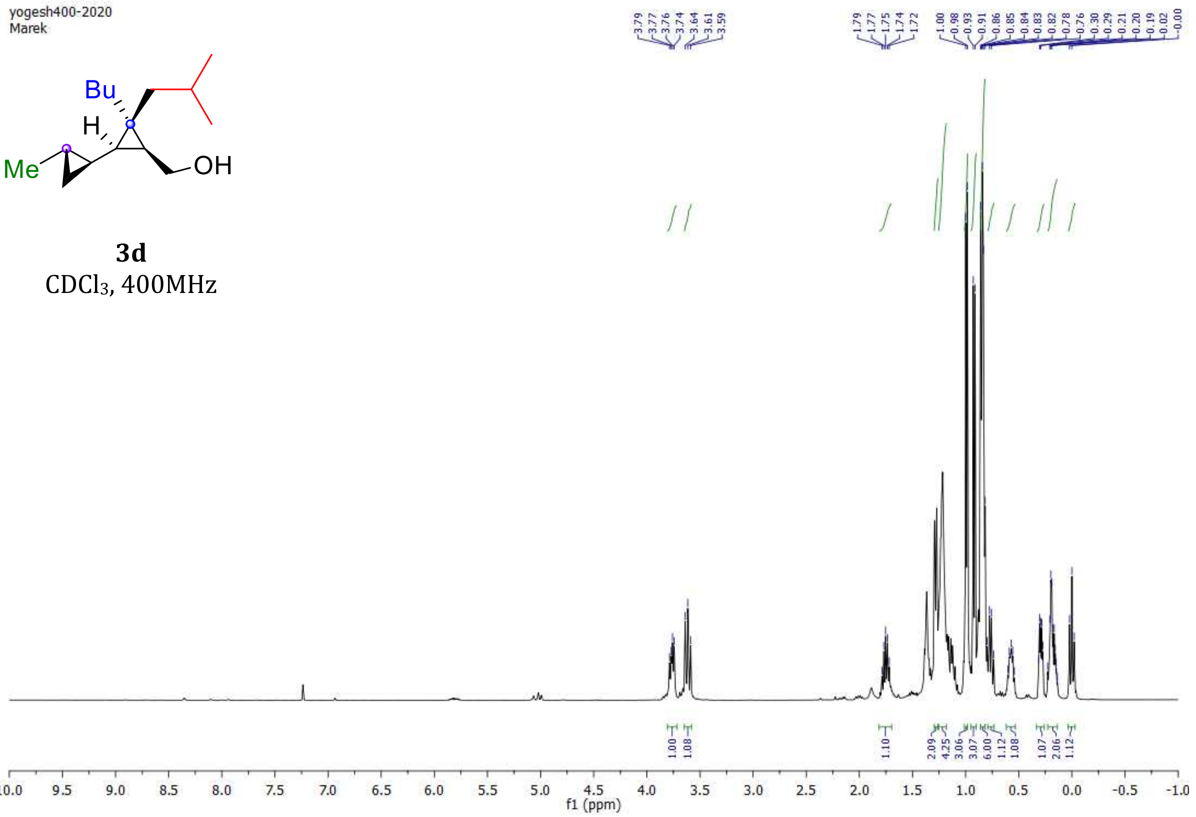
yogesh400-2020
Marek
Bu
H
Me
OH
3d
CDCl3, 400MHz
3.79 3.77 3.76 3.74 3.64 3.61 3.59
1.79 1.77 1.75 1.74 1.72
1.00 0.98 0.93 0.91 0.86 0.85 0.84 0.83 0.82 0.78 0.76 0.30 0.29 0.21 0.20 0.19 0.02 0.00
1.00 1.08 1.10 2.09 4.25 3.06 3.07 6.00 1.12 1.08 1.07 2.06 1.12
f1 (ppm)

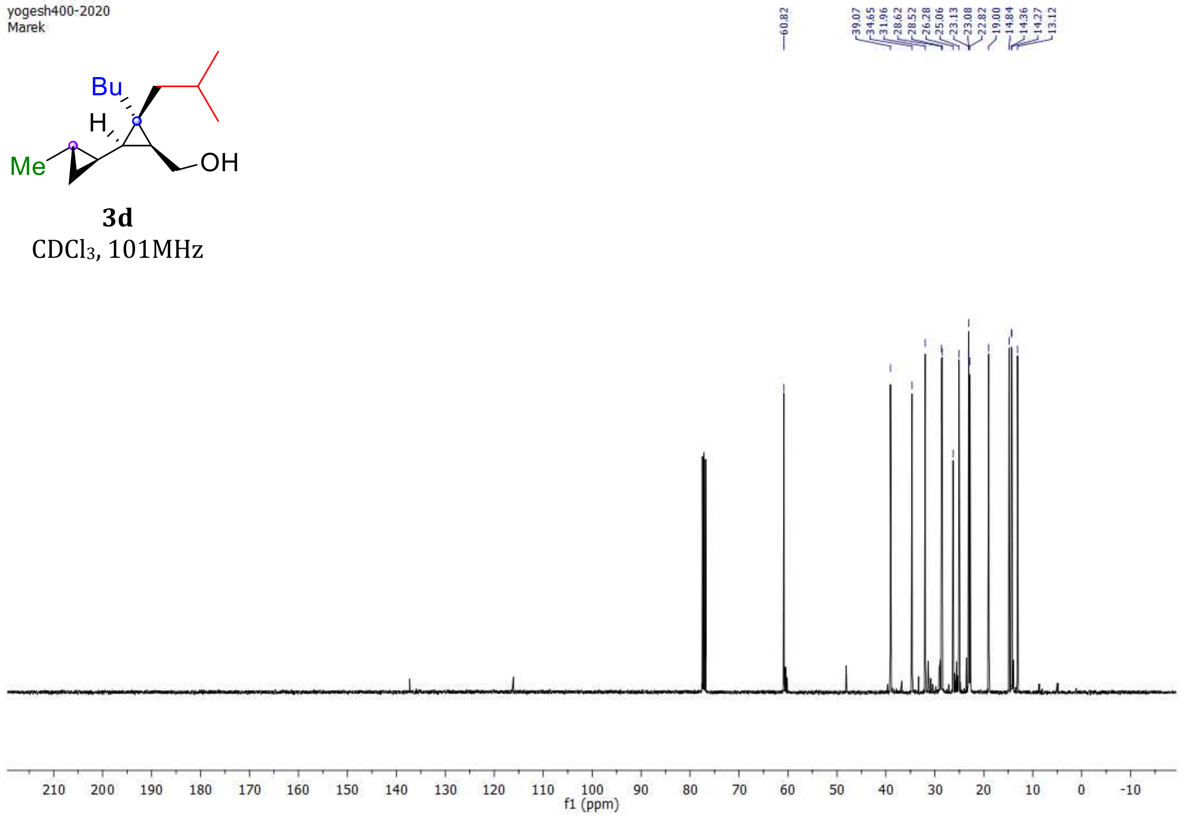
yogesh400-2020
Marek
Bu
H
Me
OH
3d
CDCl3, 101MHz
60.82
39.07 34.65 31.96 28.62 28.52 26.28 25.06 23.13 23.08 22.82 19.00 14.84 14.36 14.27 13.12
f1 (ppm)

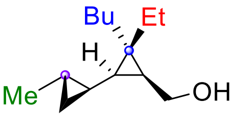

3e
$CDCl_3$, 400MHz

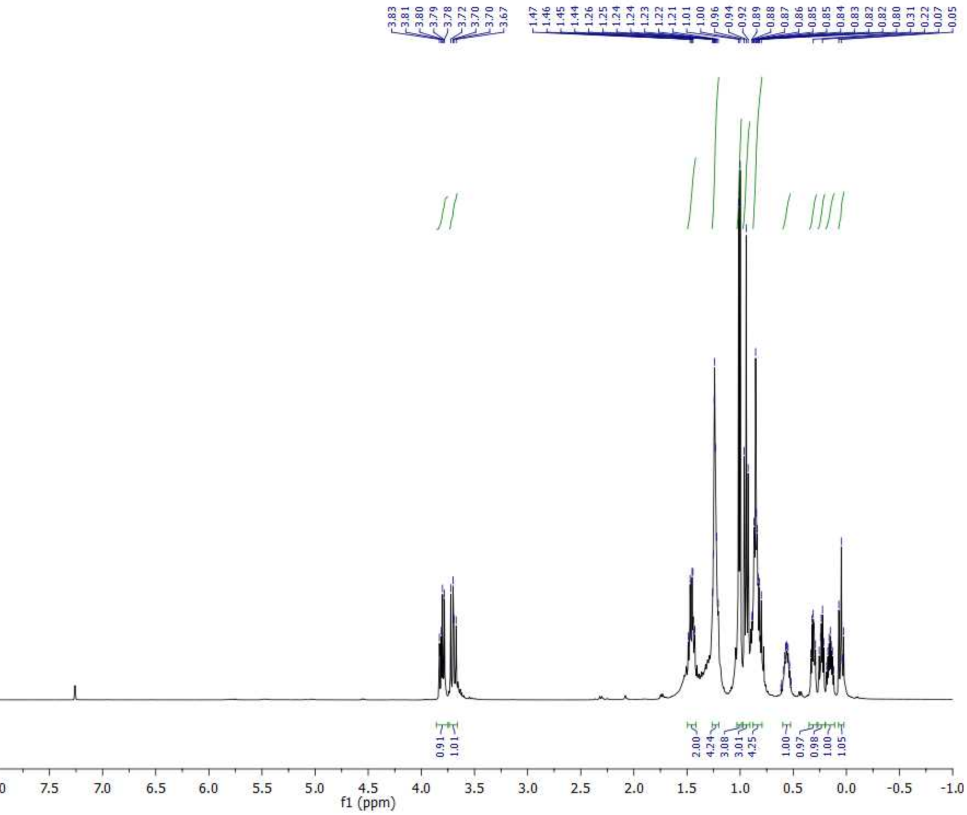

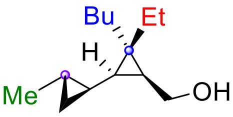

3e
$CDCl_3$, 101MHz

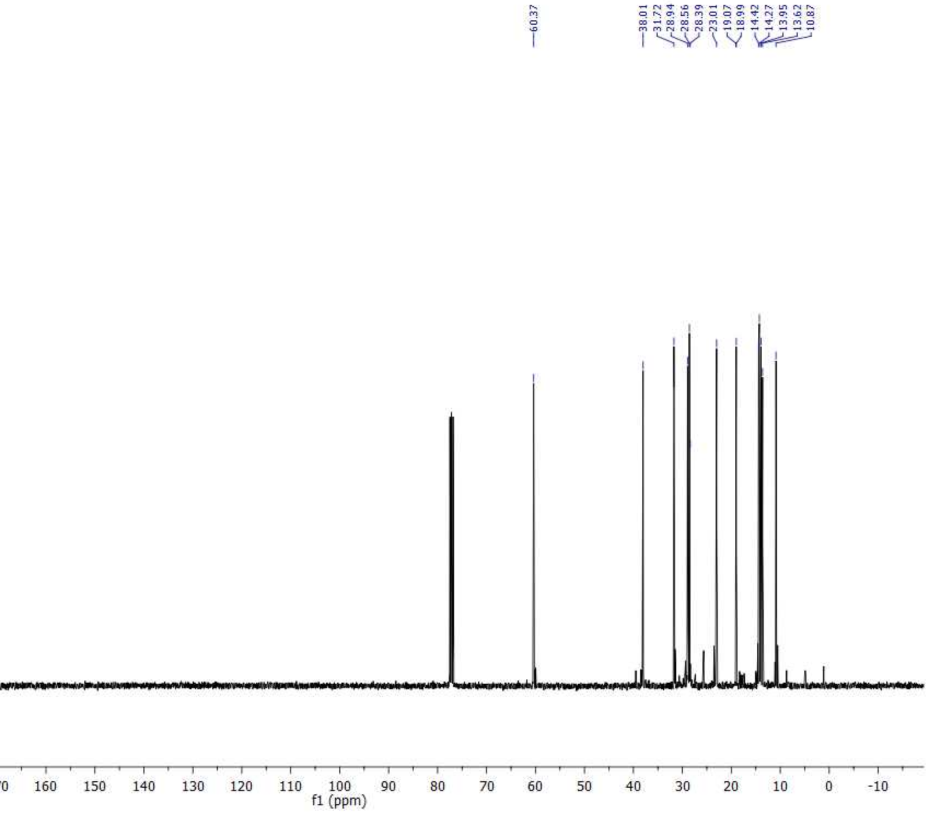

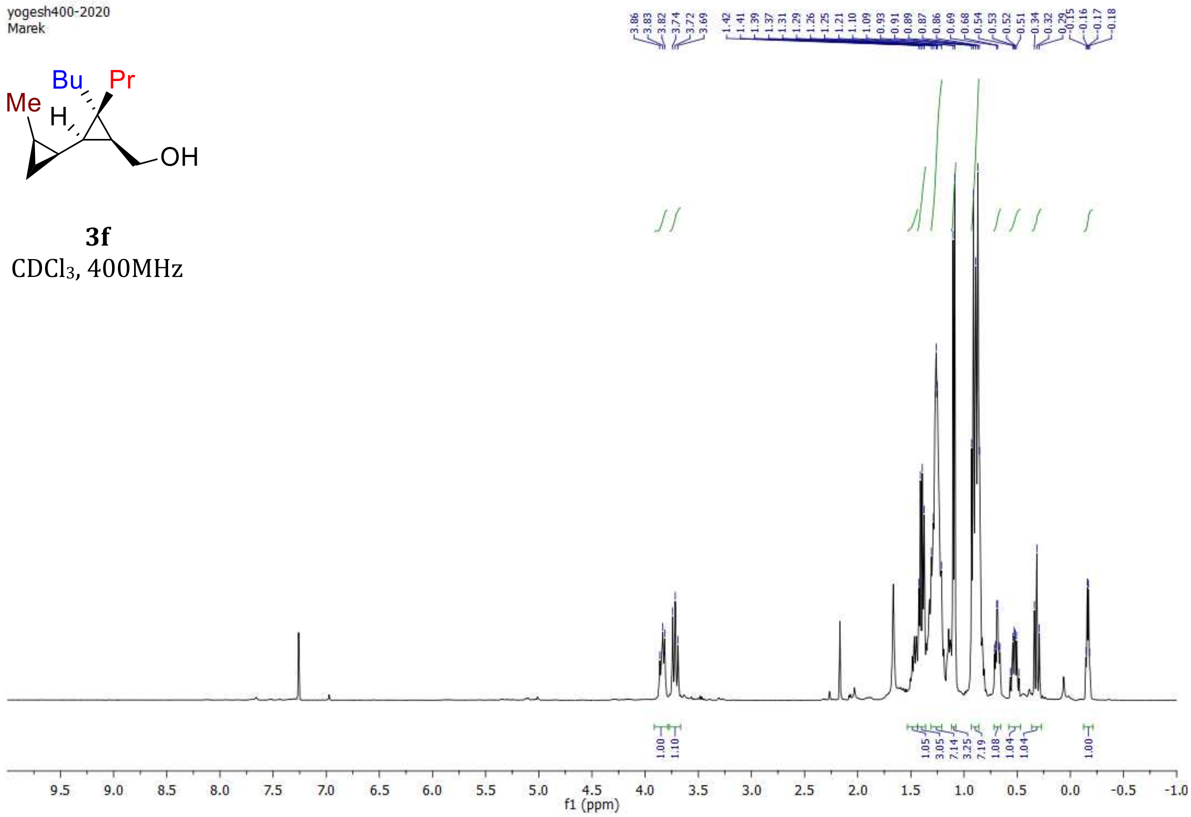
yogesh400-2020
Marek
Bu
Pr
Me
H
OH
3f
CDCl3, 400MHz
3.86 3.83 3.82 3.74 3.72 3.69 1.42 1.41 1.39 1.37 1.31 1.29 1.26 1.25 1.21 1.10 1.09 0.93 0.91 0.89 0.87 0.86 0.69 0.68 0.54 0.53 0.52 0.51 0.34 0.32 0.29 0.15 -0.16 -0.17 -0.18
1.00 1.10 1.05 3.05 7.14 3.25 7.19 1.08 1.04 1.04 1.00
f1 (ppm)

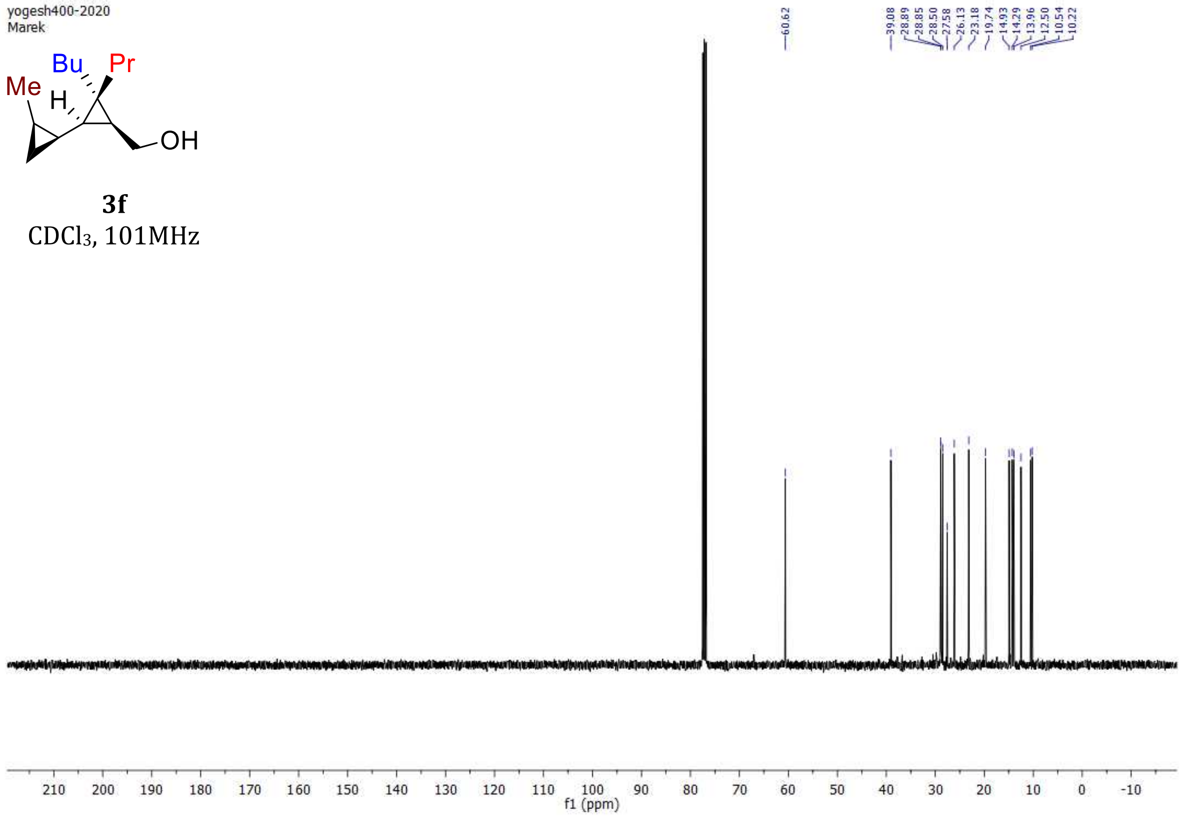
yogesh400-2020
Marek
Bu
Pr
Me
H
OH
3f
CDCl3, 101MHz
60.62
39.08 28.89 28.85 28.50 27.58 26.13 23.18 19.74 14.93 14.29 13.96 12.50 10.54 10.22
f1 (ppm)

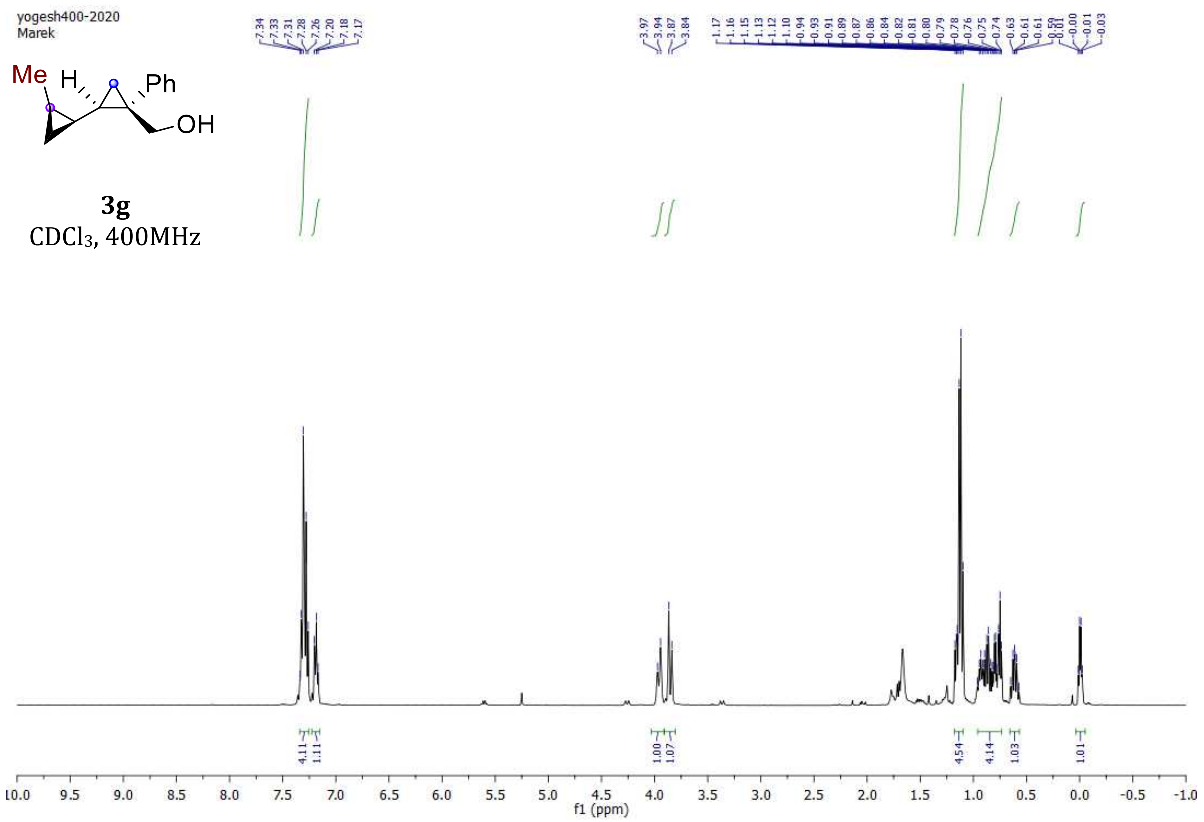
yogesh400-2020
Marek
Me
H
Ph
OH
3g
CDCl3, 400MHz
f1 (ppm)

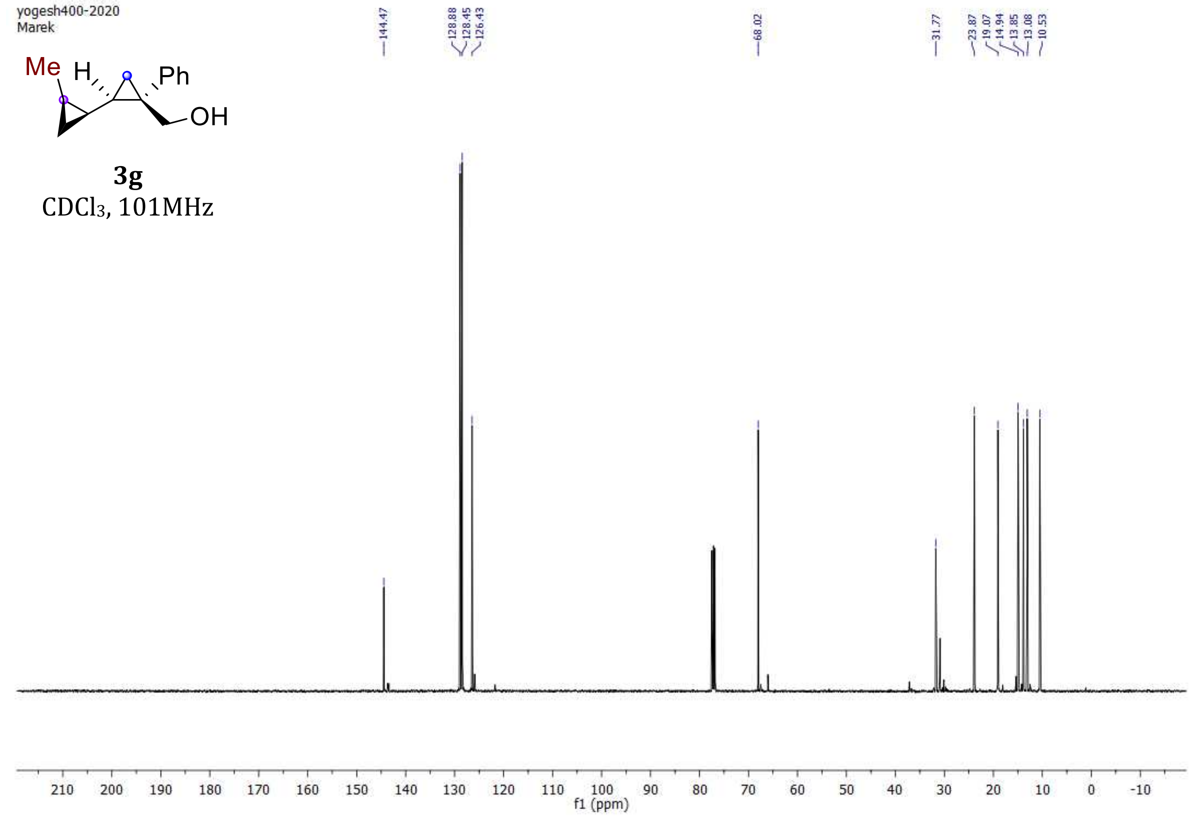
yogesh400-2020
Marek
Me
H
Ph
OH
3g
CDCl3, 101MHz
144.47
128.88
128.45
126.43
68.02
31.77
23.87
19.07
14.94
13.85
13.08
10.53
f1 (ppm)

yogesh400-2020
Marek

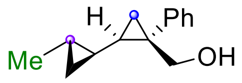

3h
CDCl3, 400MHz

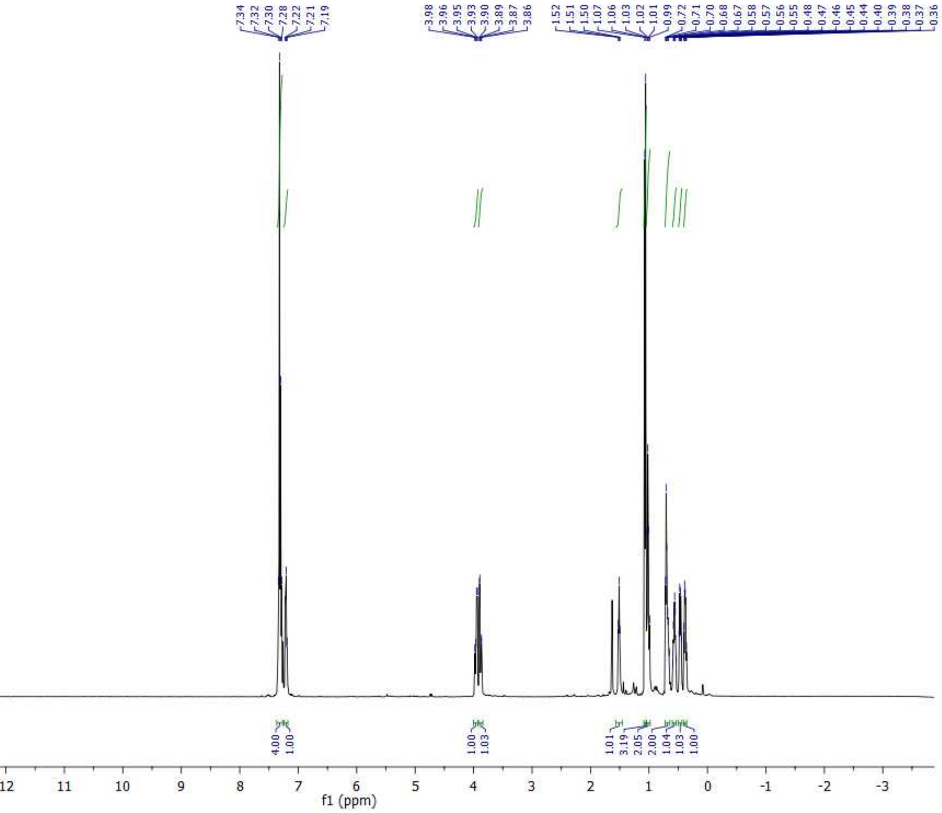

yogesh400-2020
Marek

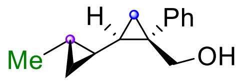

3h
$CDCl_3$, 101MHz

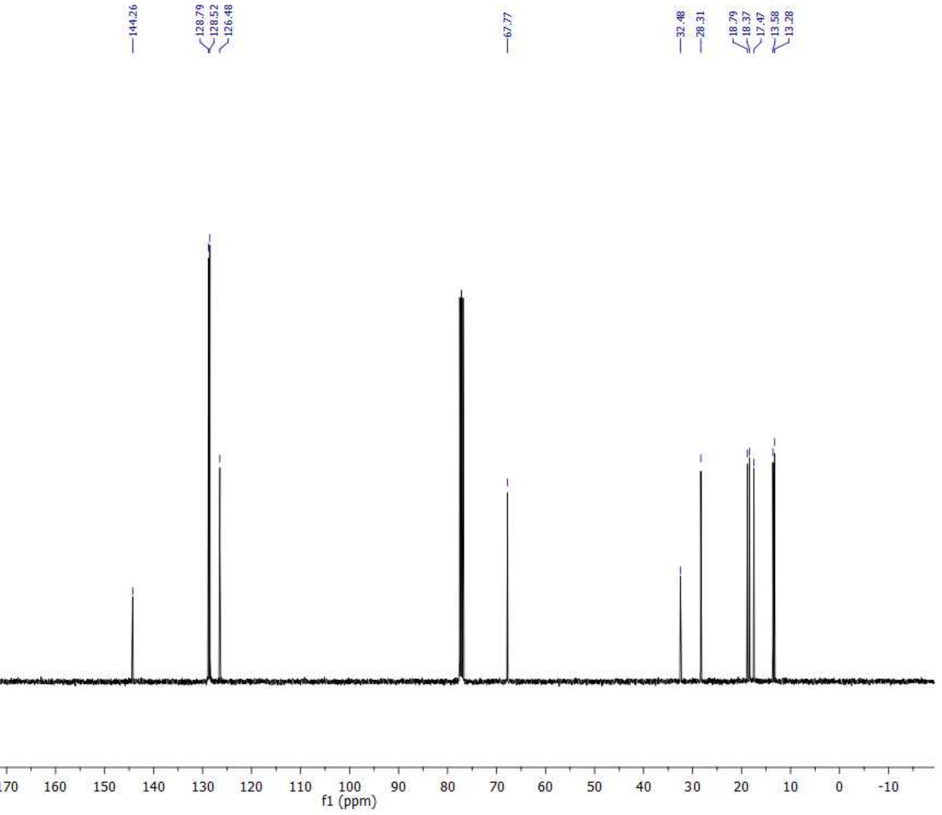

Overlay of 3g and 3h, demonstrating diastereomeric purity

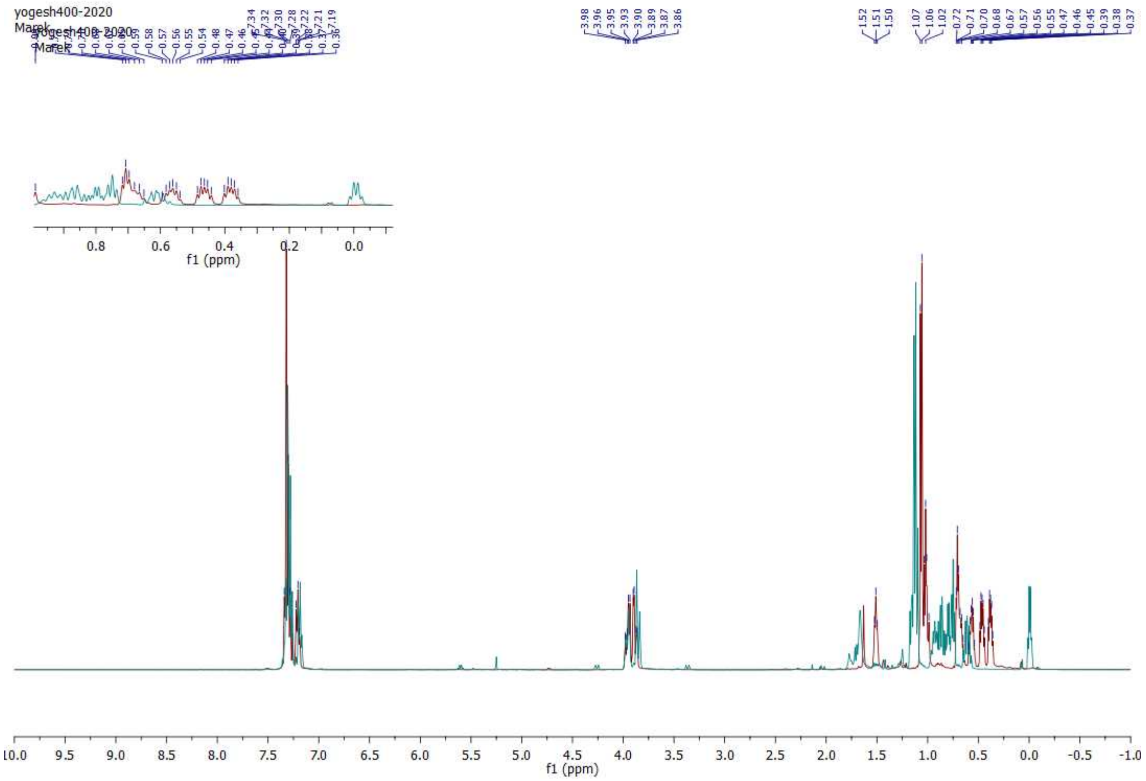

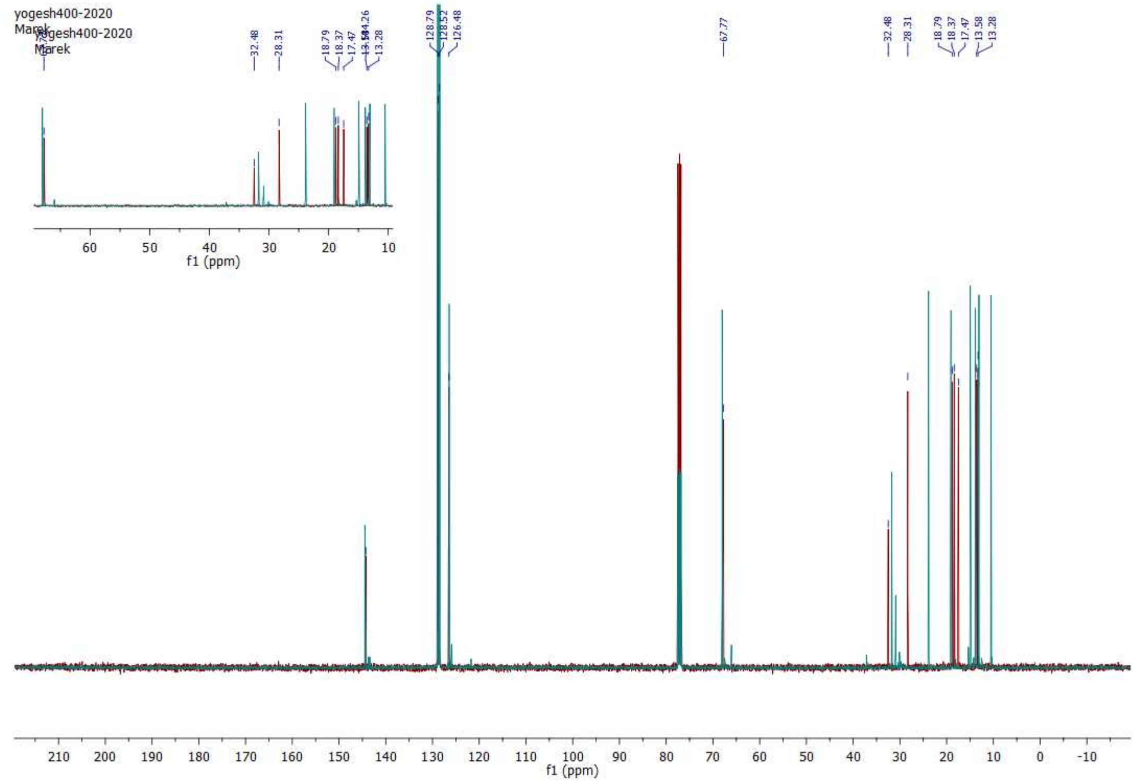

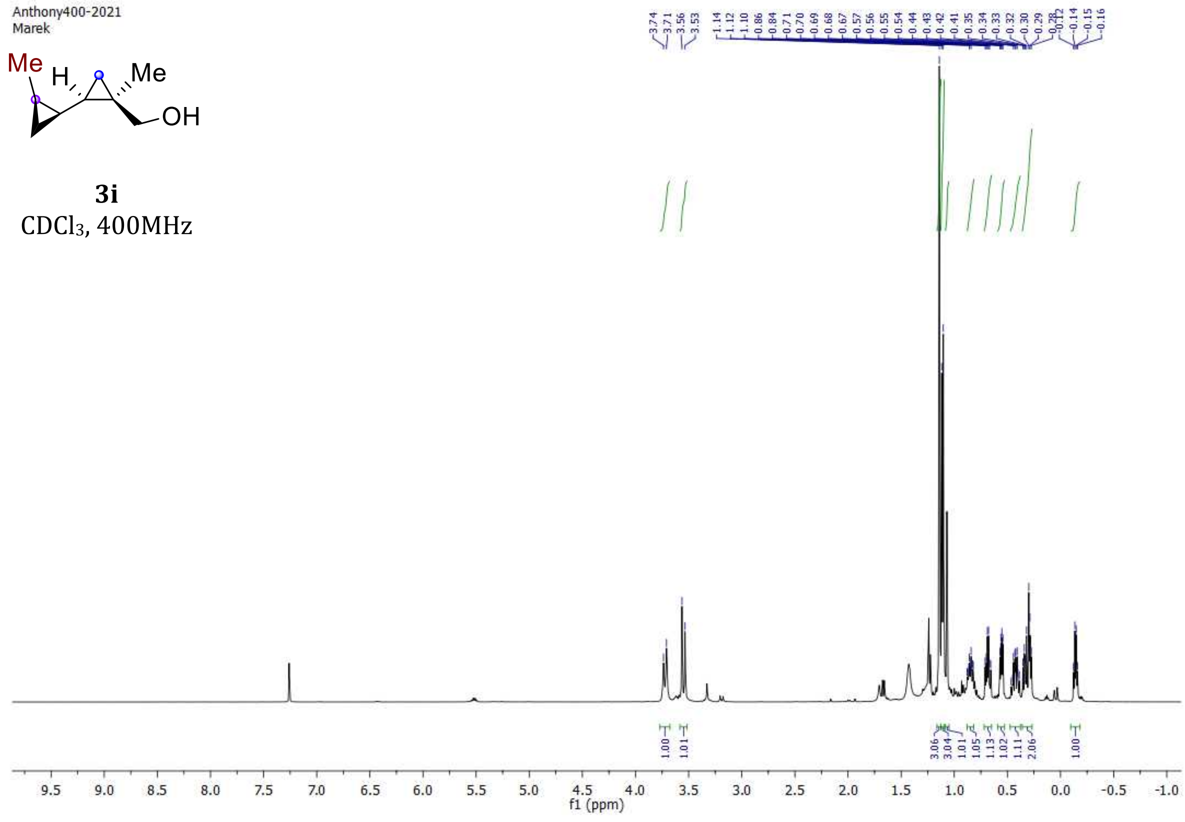
Anthony400-2021
Marek
Me
H
Me
OH
3i
CDCl3, 400MHz
f1 (ppm)

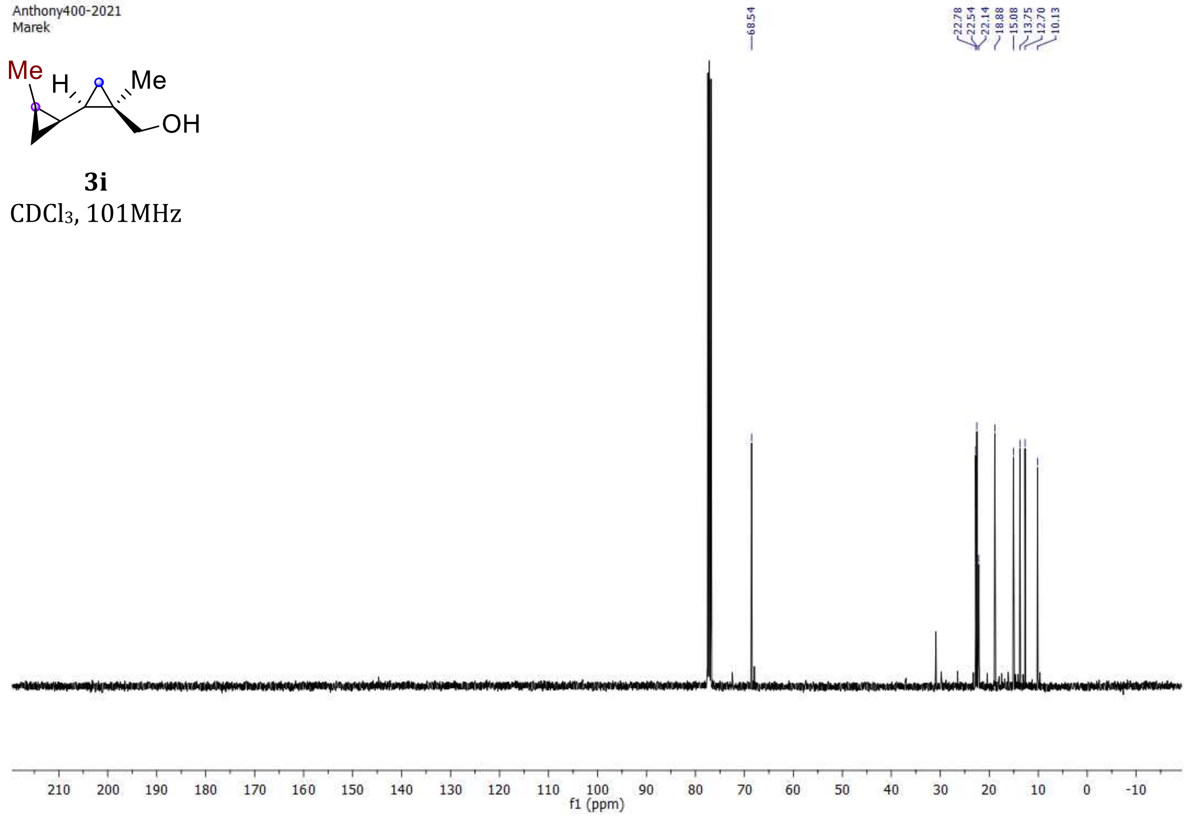
Anthony400-2021
Marek
Me
H
Me
OH
3i
CDCl3, 101MHz
f1 (ppm)

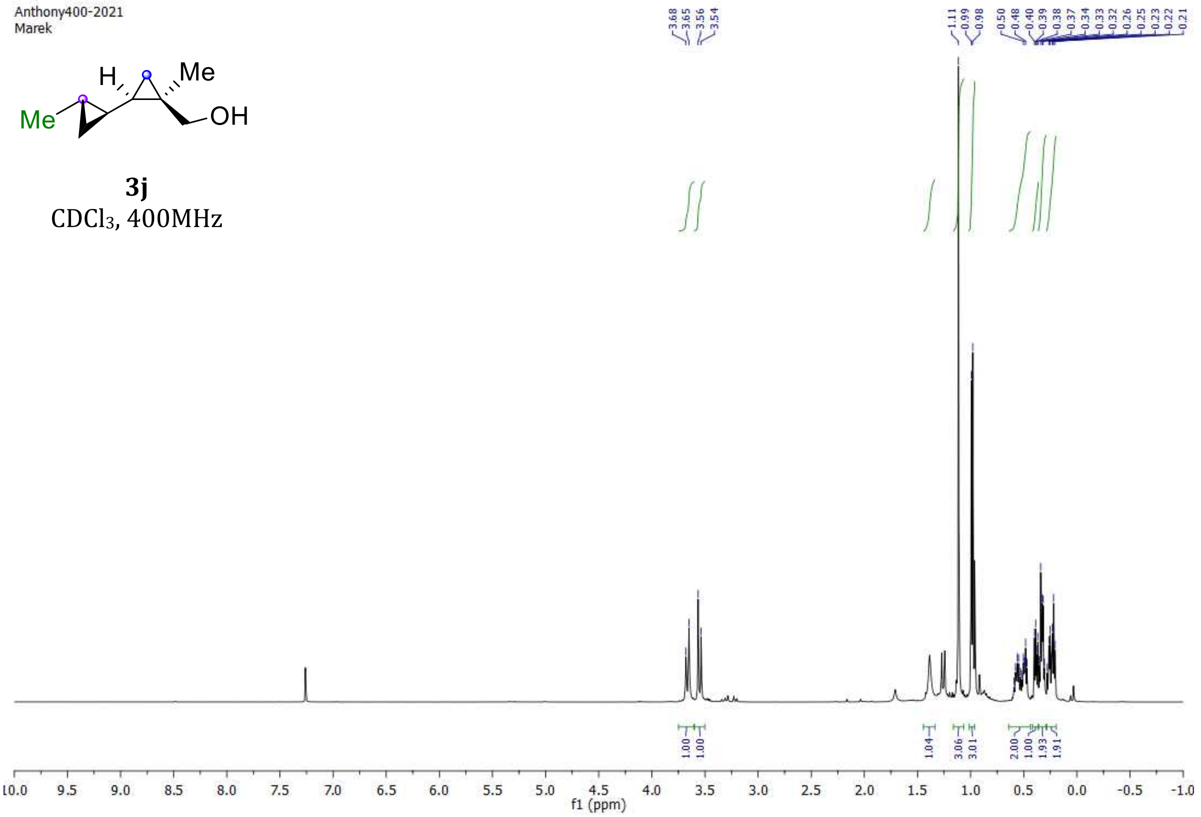

3j
$CDCl_3$, 400MHz

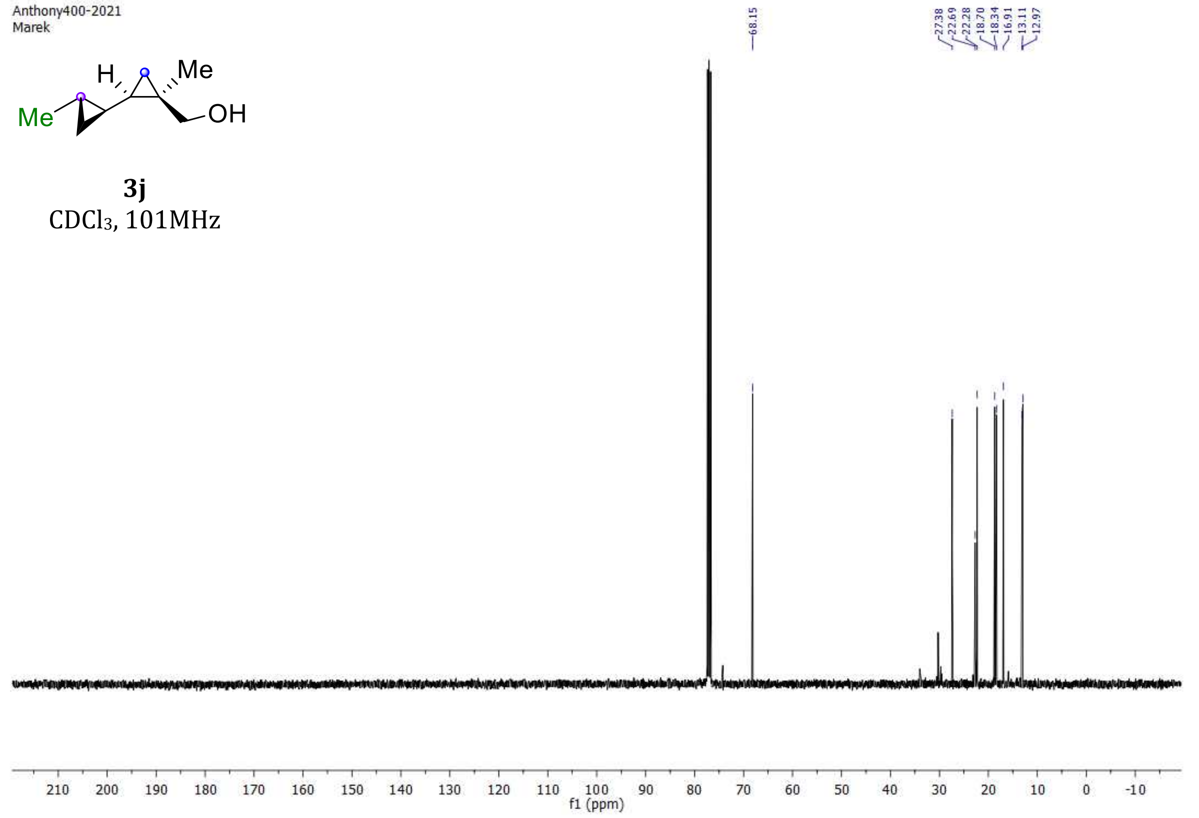

3j
$CDCl_3$, 101MHz

Overlay of 3i and 3j, demonstrating diastereomeric purity

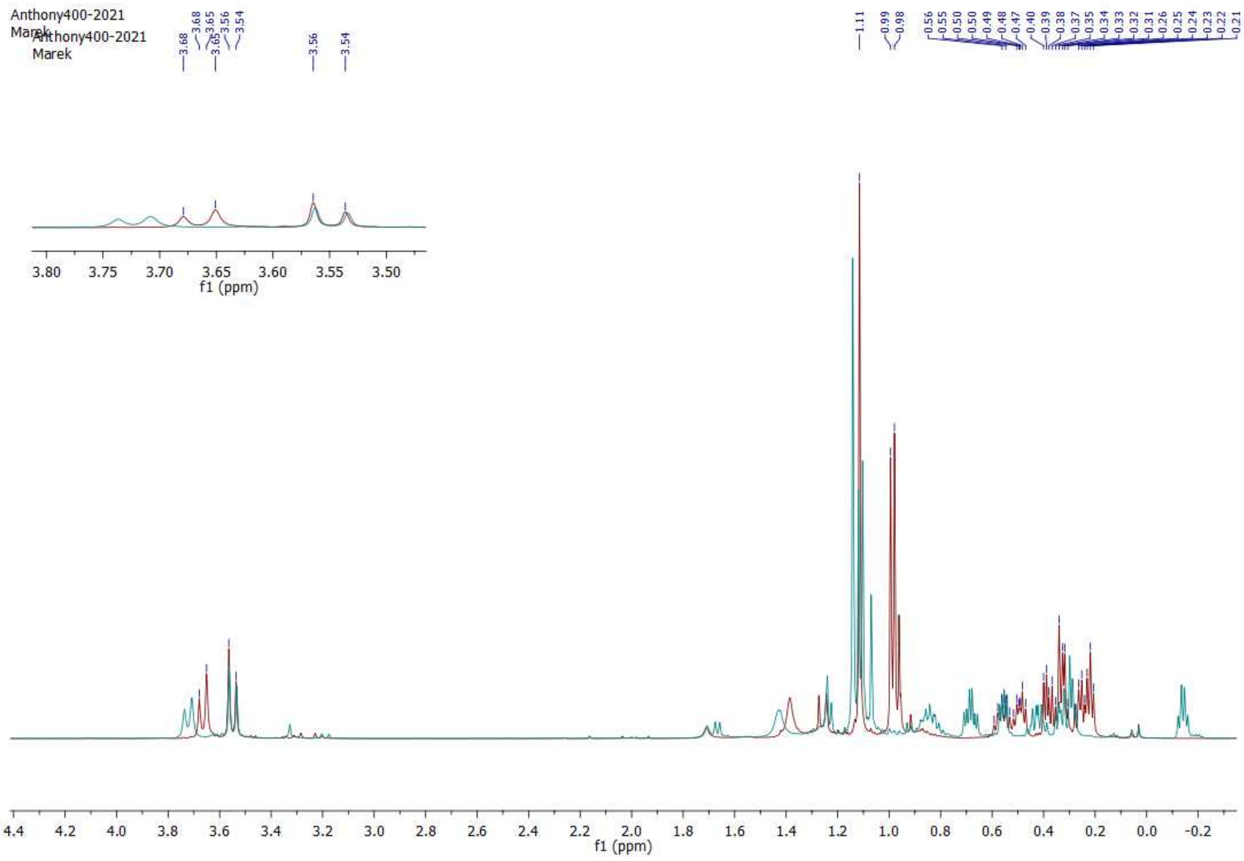

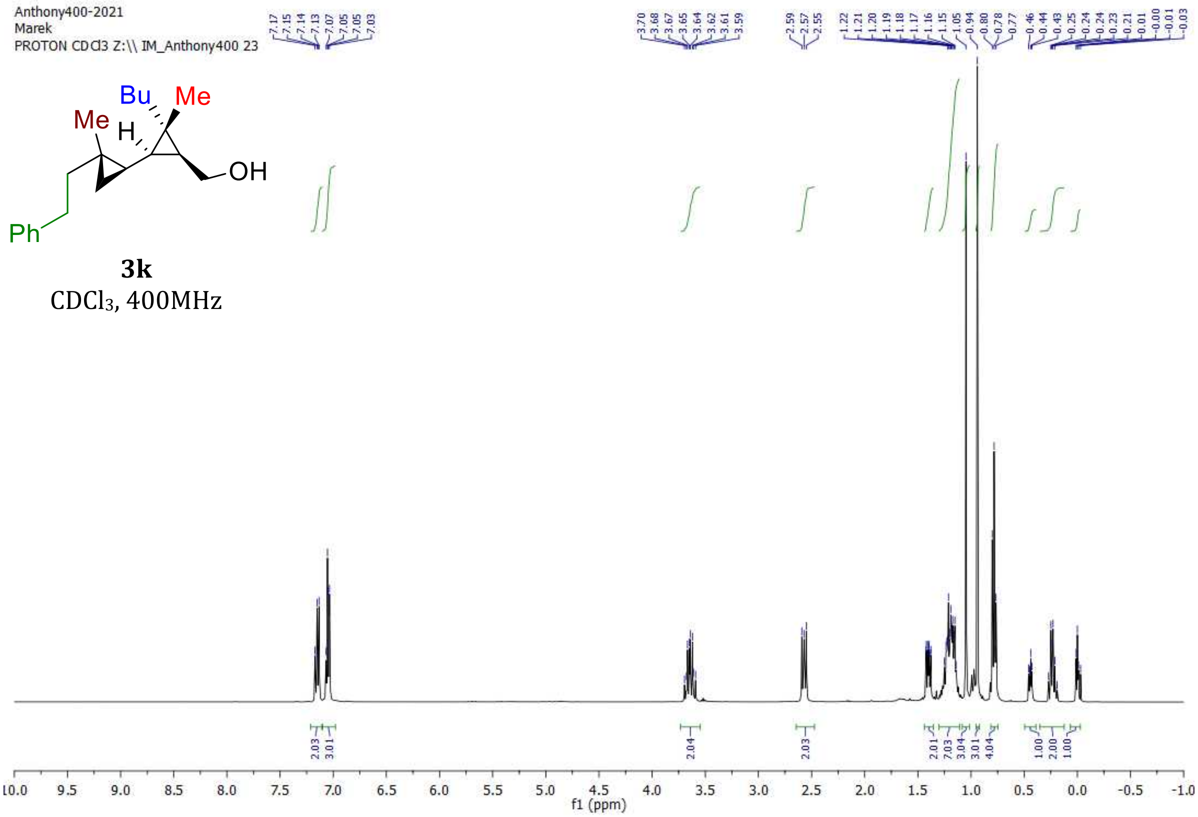
Anthony400-2021
Marek
PROTON CDCl3 Z:\\ IM_Anthony400 23
Bu
Me
Me
H
OH
Ph
3k
CDCl3, 400MHz
f1 (ppm)

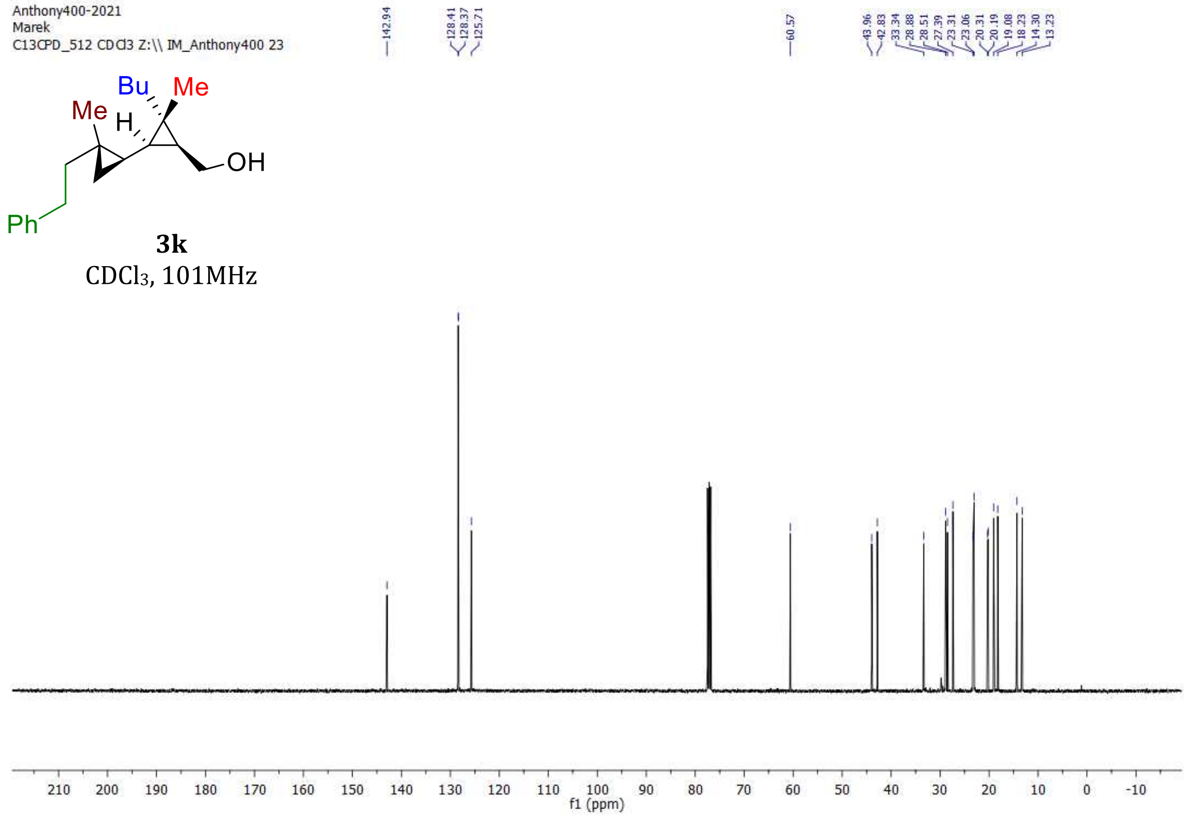
Anthony400-2021
Marek
C13CPD_512 CDCl3 Z:\\ IM_Anthony400 23
Bu
Me
Me
H
OH
Ph
3k
CDCl3, 101MHz
142.94
128.41
128.37
125.71
60.57
43.96
42.83
33.34
28.88
28.51
27.39
23.31
23.06
20.31
20.19
19.08
18.23
14.30
13.23
f1 (ppm)

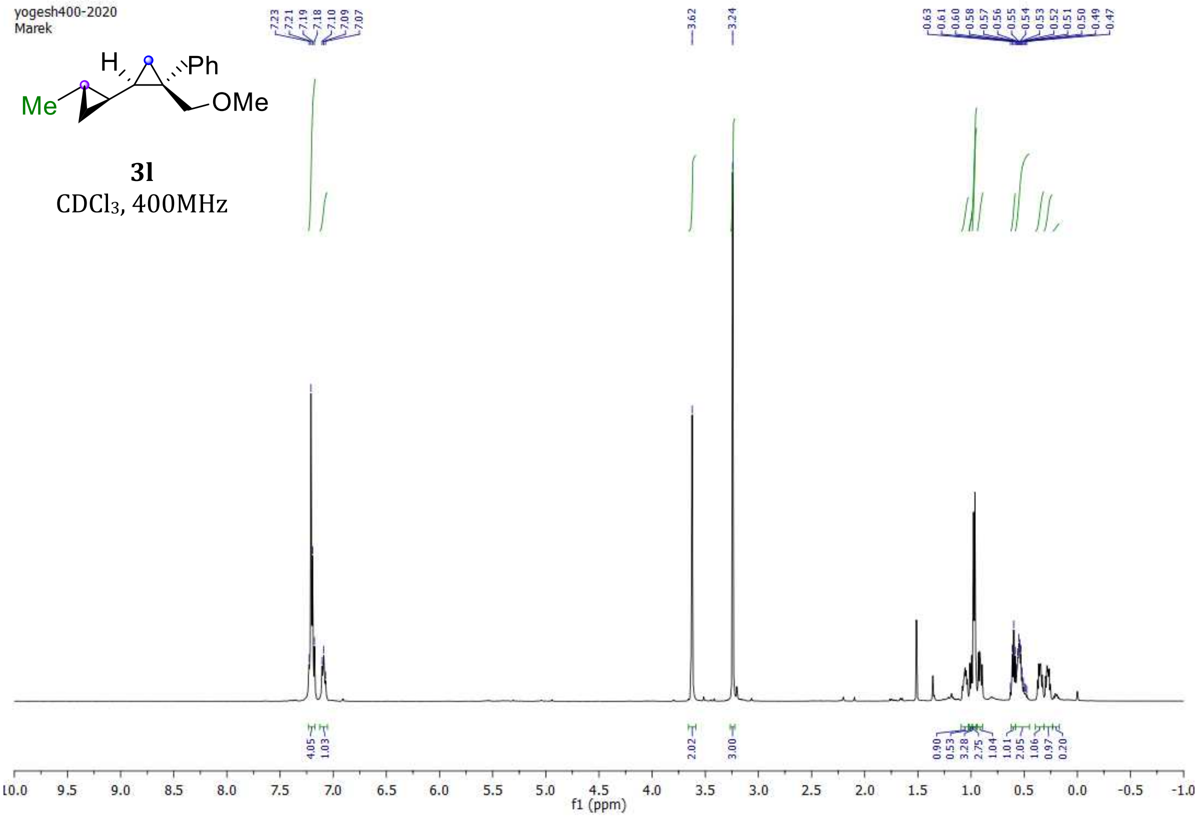
yogesh400-2020
Marek
H
Ph
Me
OMe
3l
CDCl3, 400MHz
7.23
7.21
7.19
7.18
7.10
7.09
7.07
3.62
3.24
0.63
0.61
0.60
0.58
0.57
0.56
0.55
0.54
0.53
0.52
0.51
0.50
0.49
0.47
4.05
1.03
2.02
3.00
0.90
0.53
3.28
2.75
1.04
1.01
2.05
1.06
0.97
0.20
f1 (ppm)

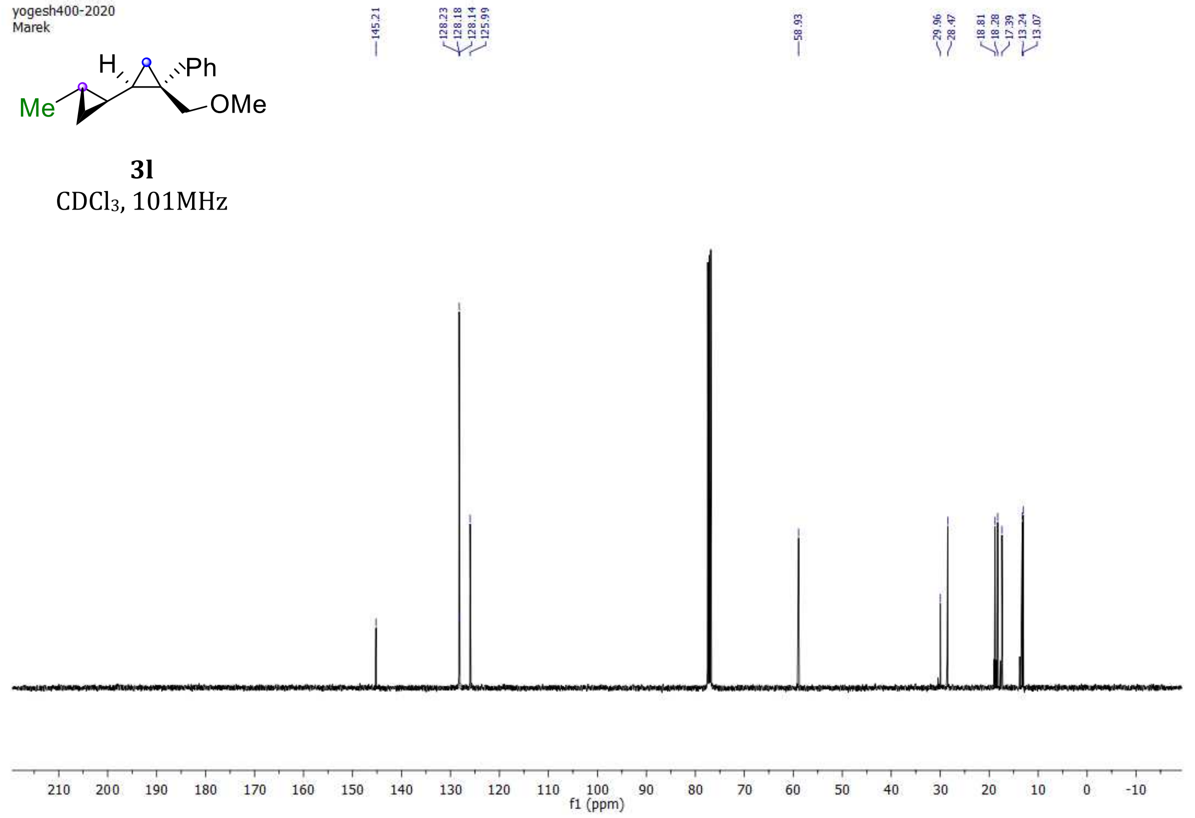
yogesh400-2020
Marek
H
Ph
Me
OMe
3l
CDCl3, 101MHz
145.21
128.23
128.18
128.14
125.99
58.93
29.96
28.47
18.81
18.28
17.39
13.24
13.07
f1 (ppm)

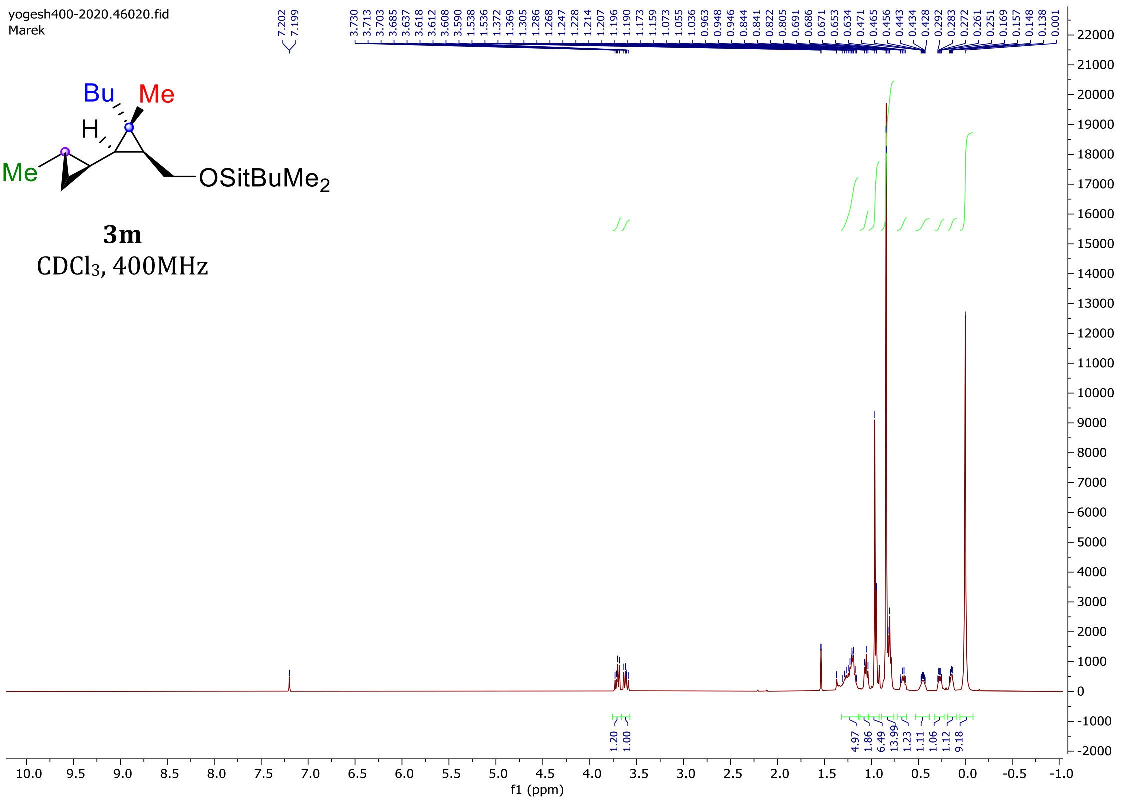

3m
CDCl$_3$, 400MHz

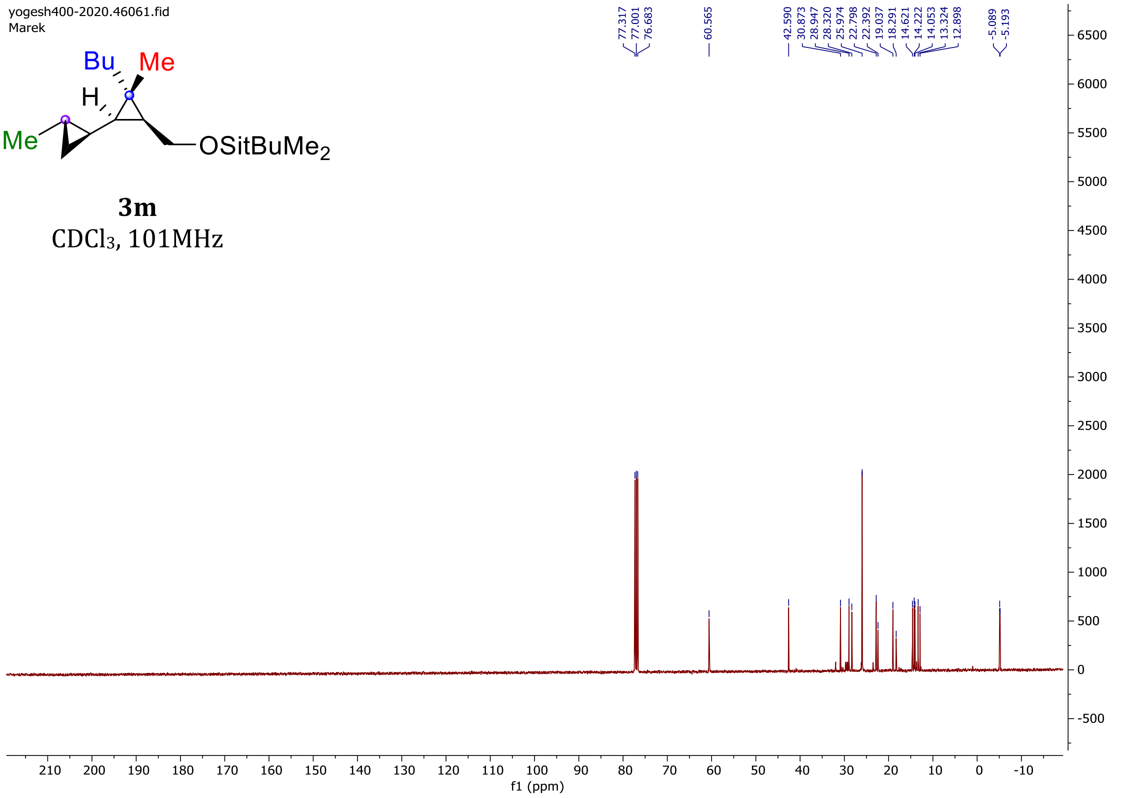

3m
CDCl$_3$, 101MHz

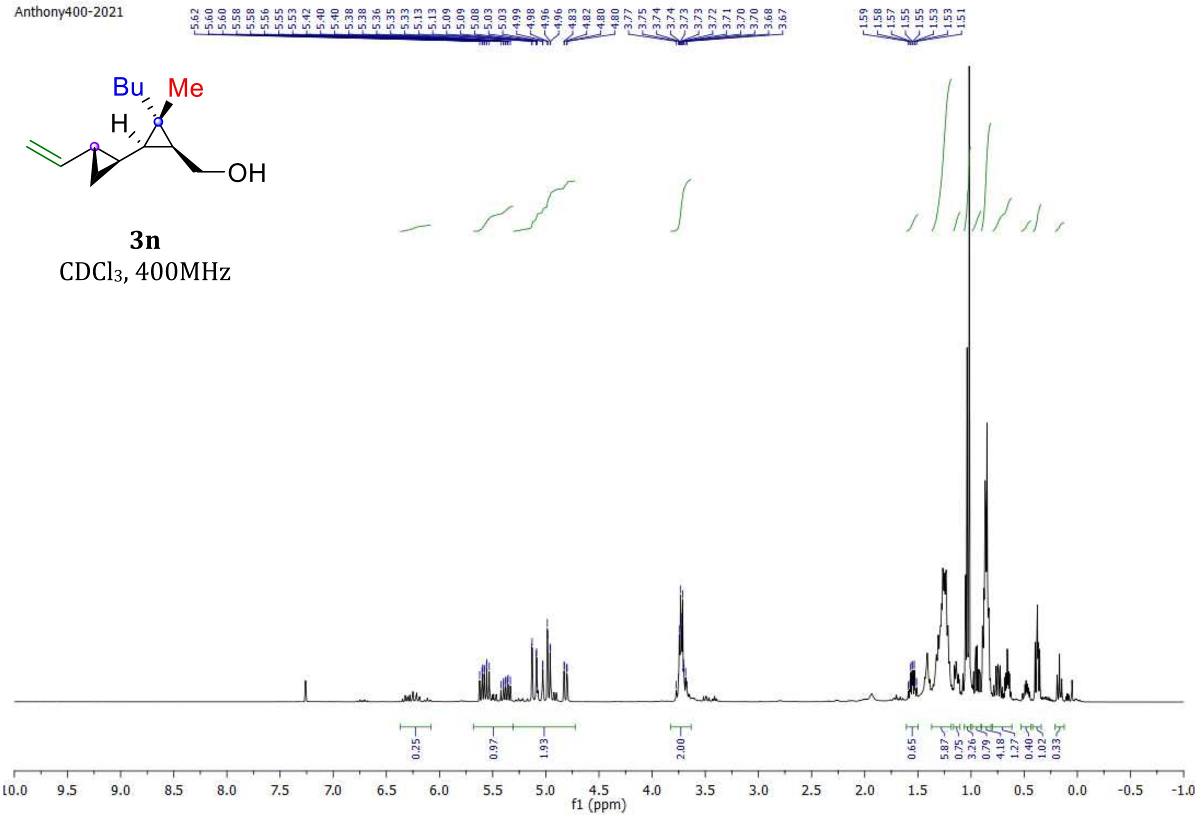
Anthony400-2021
5.62 5.60 5.60 5.58 5.56 5.55 5.53 5.42 5.40 5.40 5.38 5.38 5.36 5.35 5.33 5.33 5.13 5.13 5.09 5.09 5.08 5.03 5.03 4.99 4.98 4.96 4.96 4.83 4.82 4.80 4.80 3.77 3.75 3.74 3.73 3.73 3.72 3.71 3.70 3.70 3.68 3.67
1.59 1.58 1.57 1.55 1.55 1.53 1.53 1.51
Bu
Me
H
OH
3n
CDCl3, 400MHz
0.25 0.97 1.93 2.00 0.65 5.87 0.75 3.26 0.79 4.18 1.27 0.40 1.02 0.33
10.0 9.5 9.0 8.5 8.0 7.5 7.0 6.5 6.0 5.5 5.0 4.5 4.0 3.5 3.0 2.5 2.0 1.5 1.0 0.5 0.0 -0.5 -1.0
f1 (ppm)

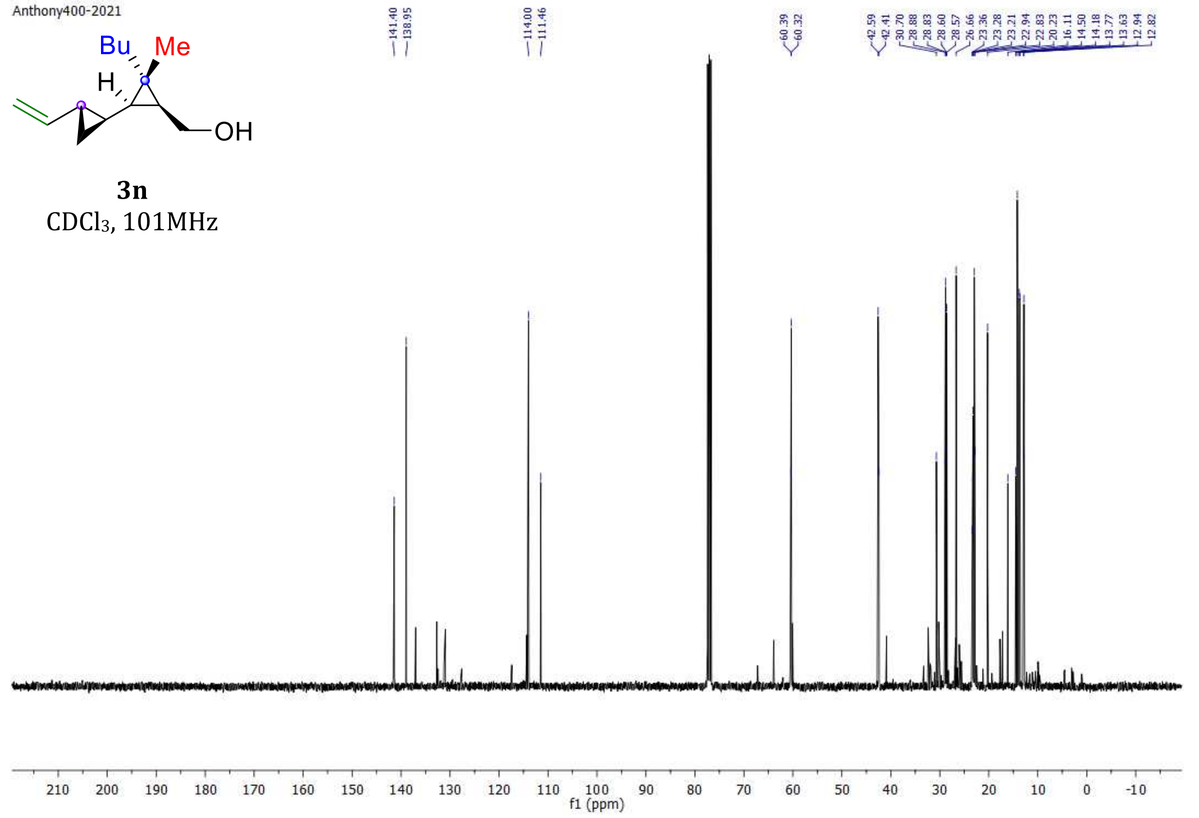
Anthony400-2021
141.40 138.95
114.00 111.46
60.39 60.32
42.59 42.41 30.70 28.88 28.83 28.60 28.57 26.66 23.36 23.28 23.21 22.94 22.83 20.23 16.11 14.50 14.18 13.77 13.63 12.94 12.82
Bu
Me
H
OH
3n
CDCl3, 101MHz
210 200 190 180 170 160 150 140 130 120 110 100 90 80 70 60 50 40 30 20 10 0 -10
f1 (ppm)

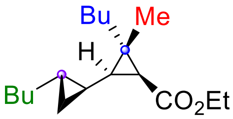

3p
$CDCl_3$, 400MHz

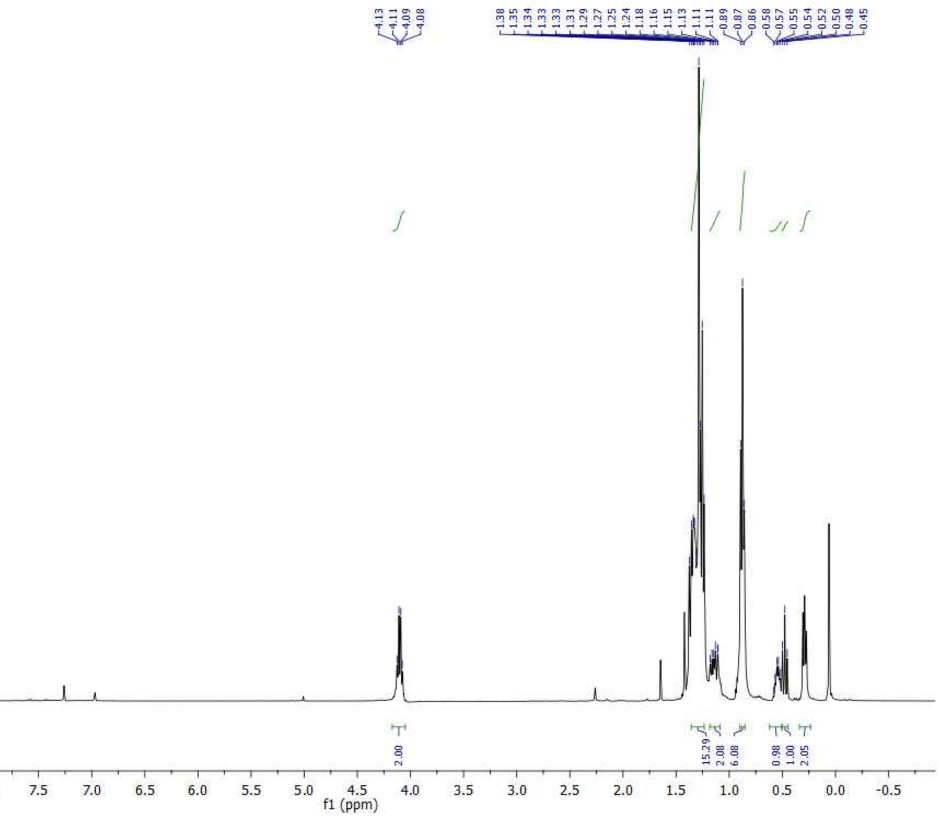

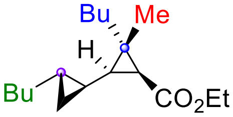

3p
$CDCl_3$, 101MHz

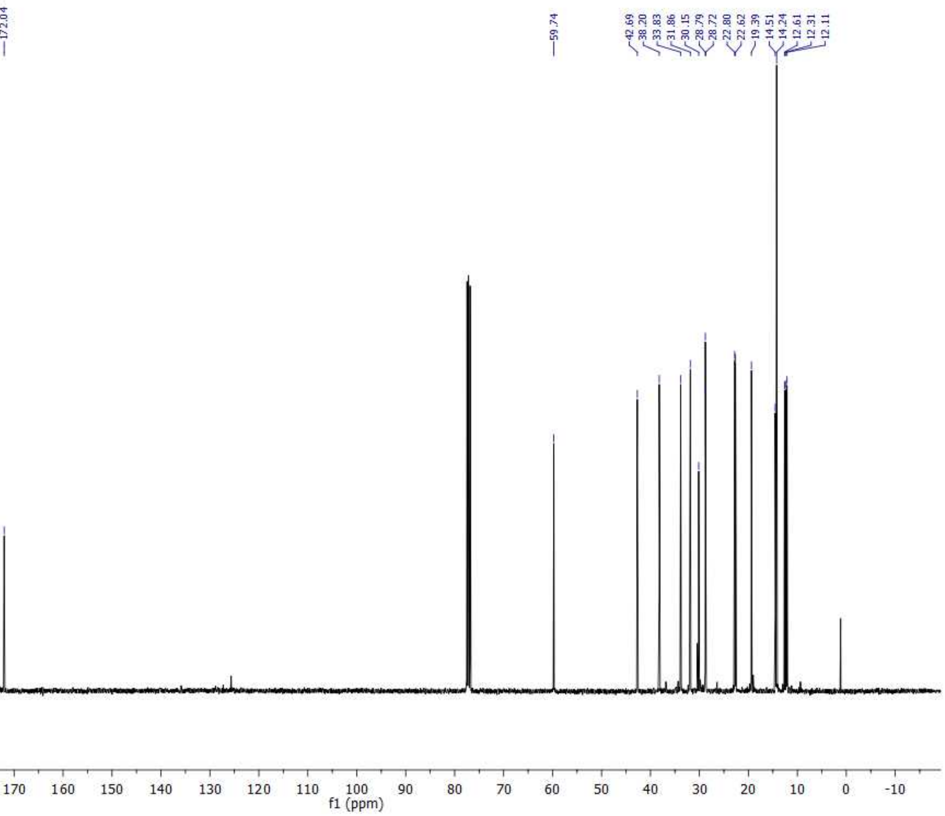

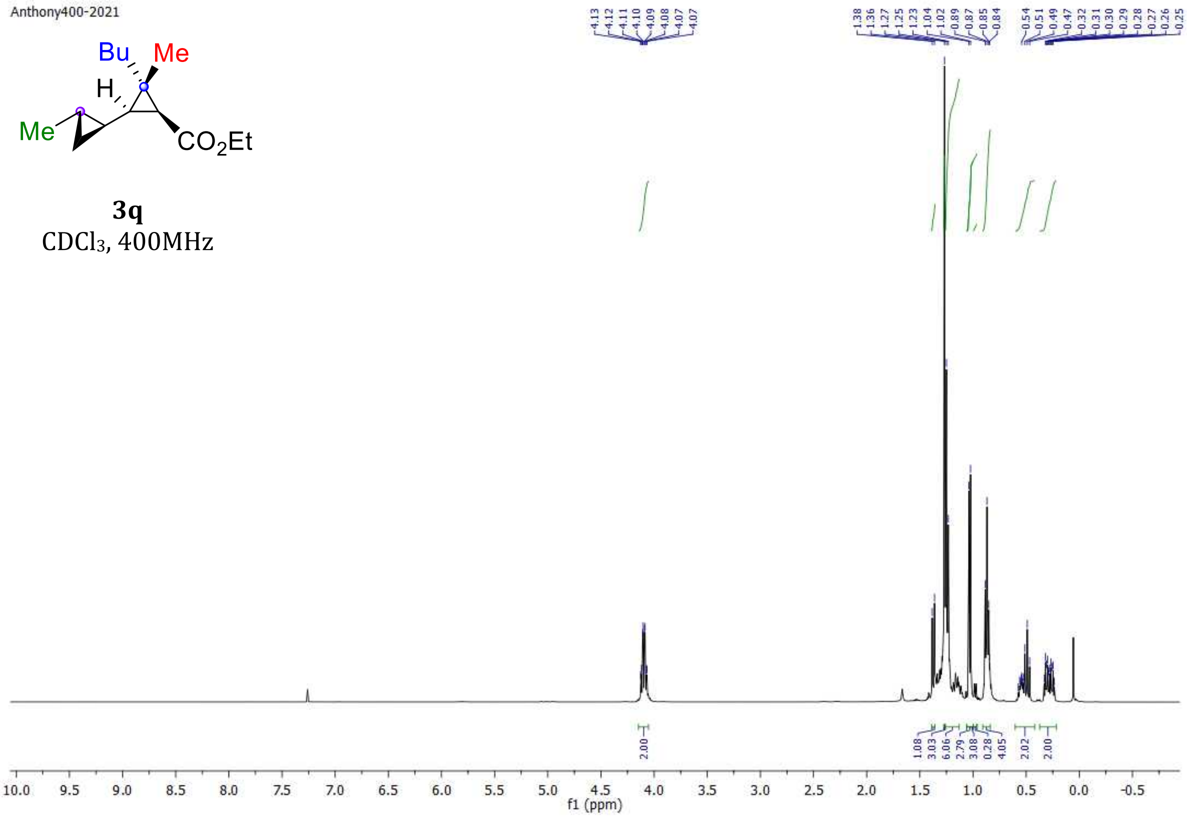
Anthony400-2021
Bu
Me
H
Me
CO2Et
3q
CDCl3, 400MHz
4.13
4.12
4.11
4.10
4.09
4.08
4.07
4.07
1.38
1.36
1.27
1.25
1.23
1.04
1.02
0.89
0.87
0.85
0.84
0.54
0.51
0.49
0.47
0.32
0.31
0.30
0.29
0.28
0.27
0.26
0.25
2.00
1.08
3.03
6.06
2.79
3.08
0.28
4.05
2.02
2.00
10.0
9.5
9.0
8.5
8.0
7.5
7.0
6.5
6.0
5.5
5.0
4.5
4.0
3.5
3.0
2.5
2.0
1.5
1.0
0.5
0.0
-0.5
f1 (ppm)

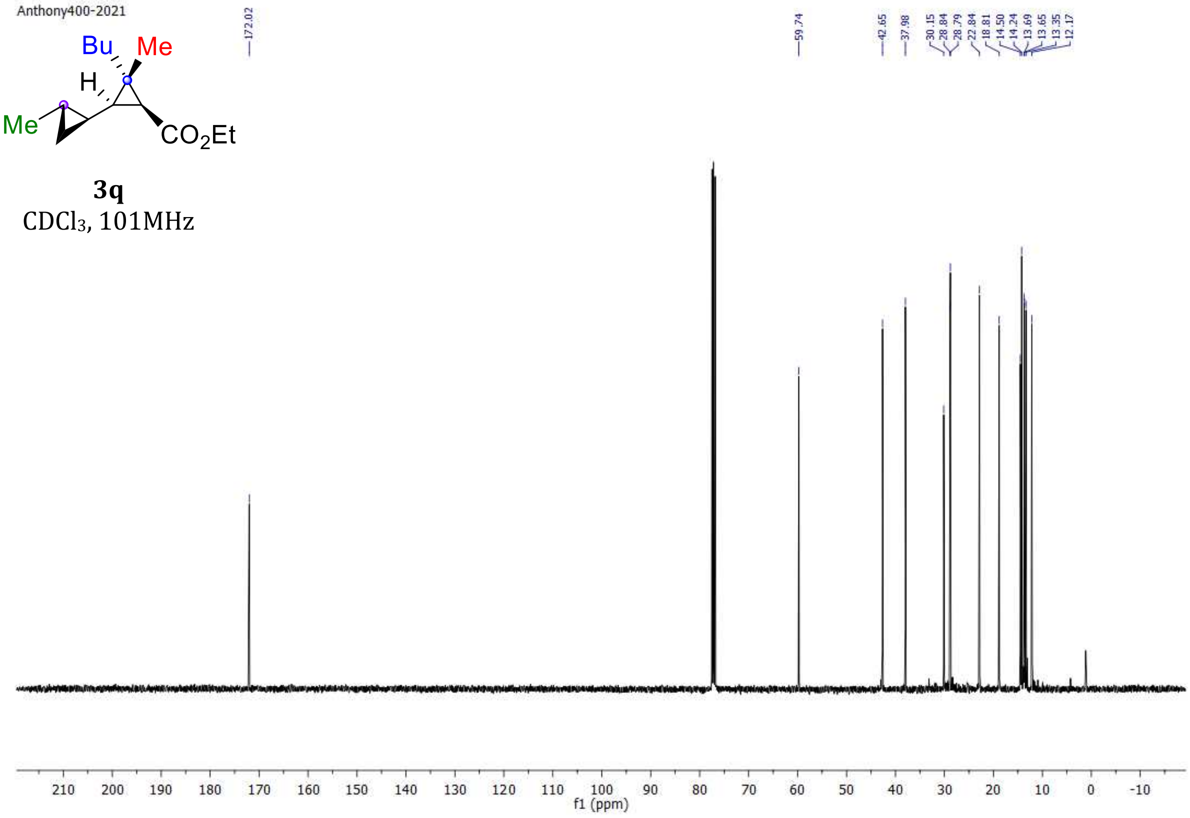
Anthony400-2021
Bu
Me
H
Me
CO2Et
3q
CDCl3, 101MHz
172.02
59.74
42.65
37.98
30.15
28.84
28.79
22.84
18.81
14.50
14.24
13.69
13.65
13.35
12.17
210
200
190
180
170
160
150
140
130
120
110
100
90
80
70
60
50
40
30
20
10
0
-10
f1 (ppm)

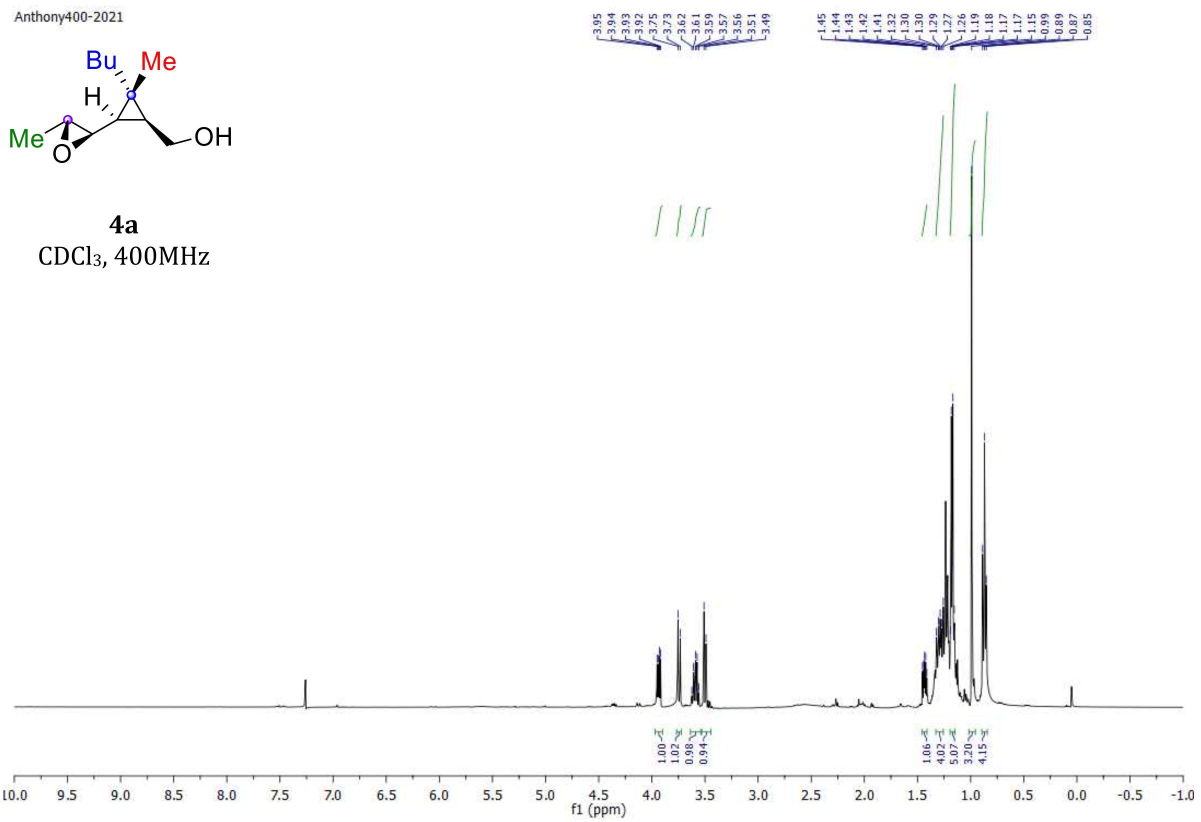
Anthony400-2021
Bu
Me
H
Me
O
OH
4a
CDCl3, 400MHz
f1 (ppm)

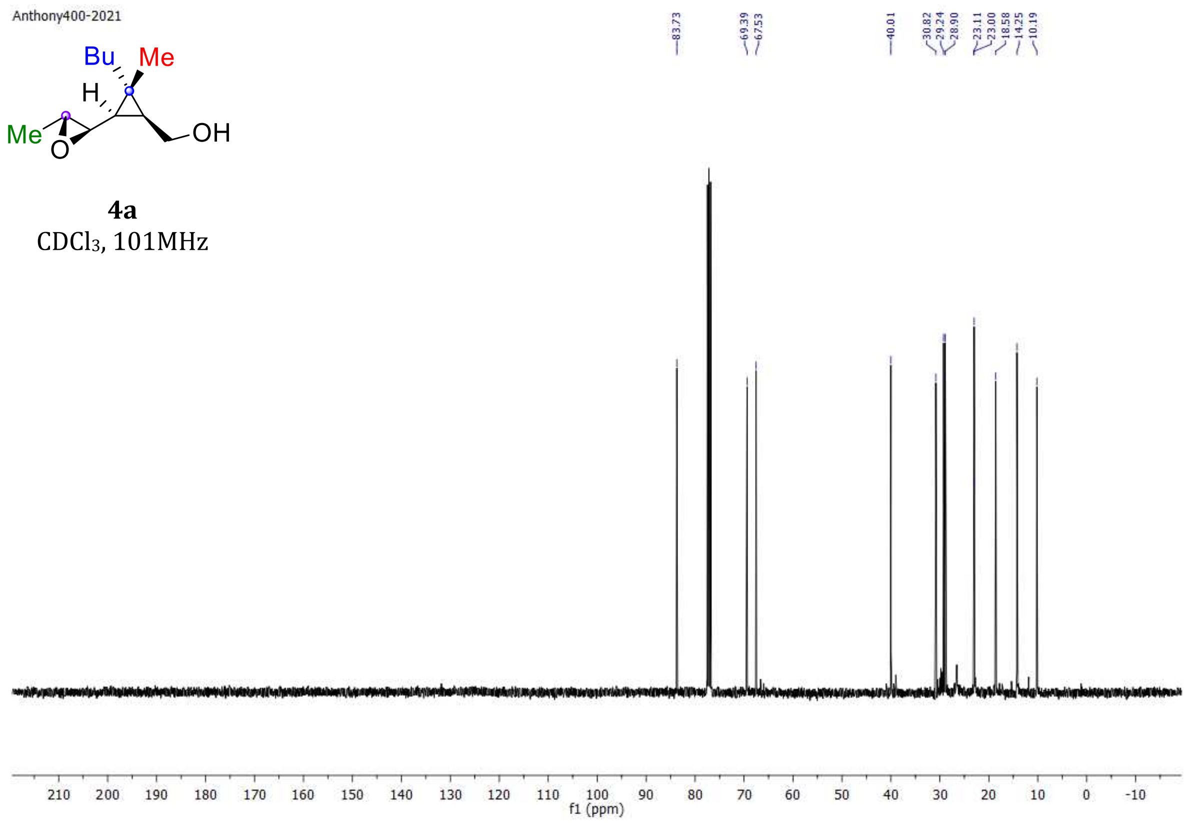
Anthony400-2021
Bu
Me
H
Me
O
OH
4a
CDCl3, 101MHz
f1 (ppm)

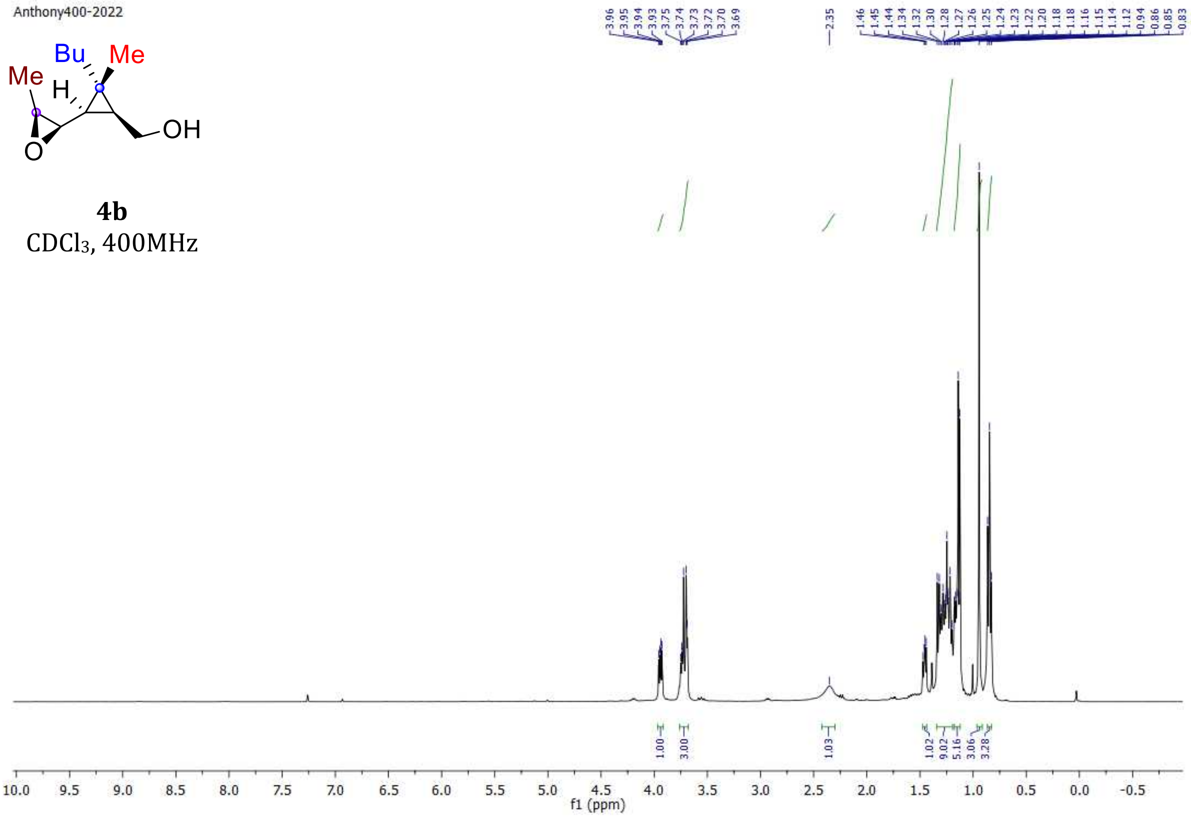

4b
$CDCl_3$, 400MHz

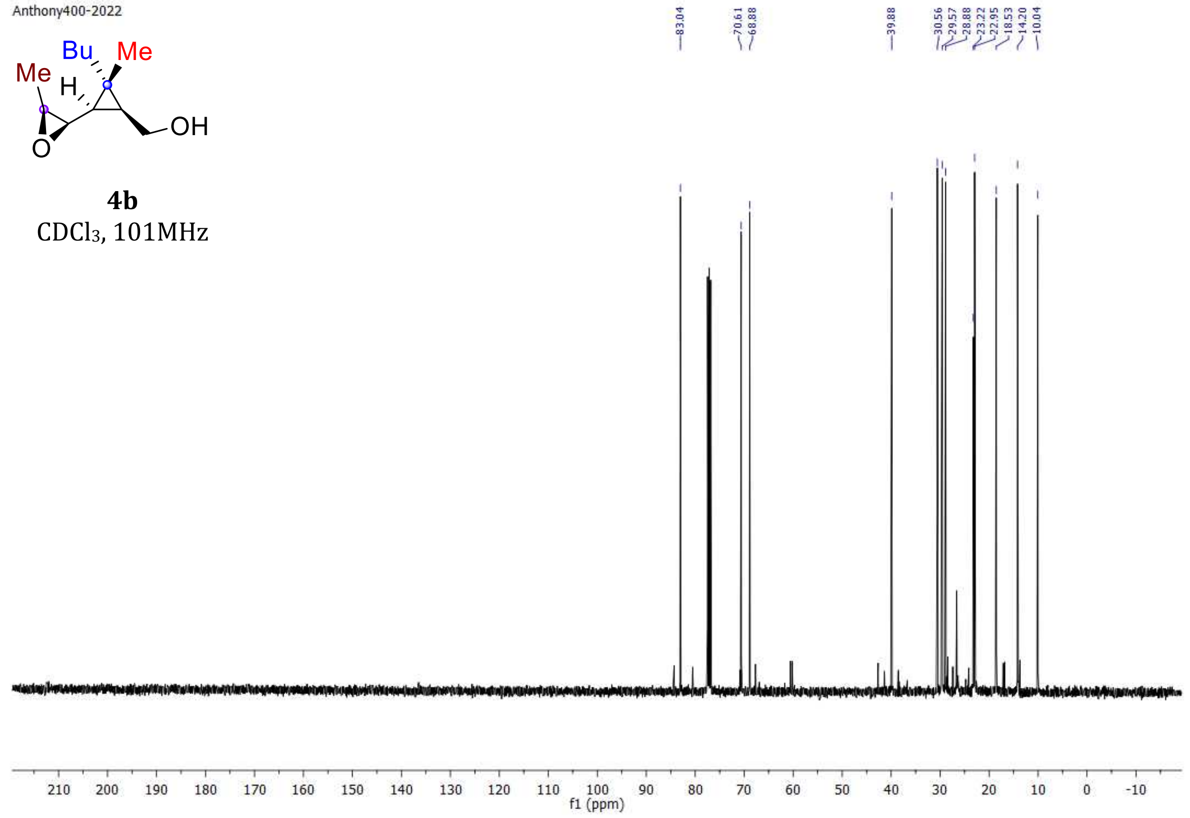

4b
$CDCl_3$, 101MHz

Overlay of 4a and 4b, demonstrating diastereomeric purity

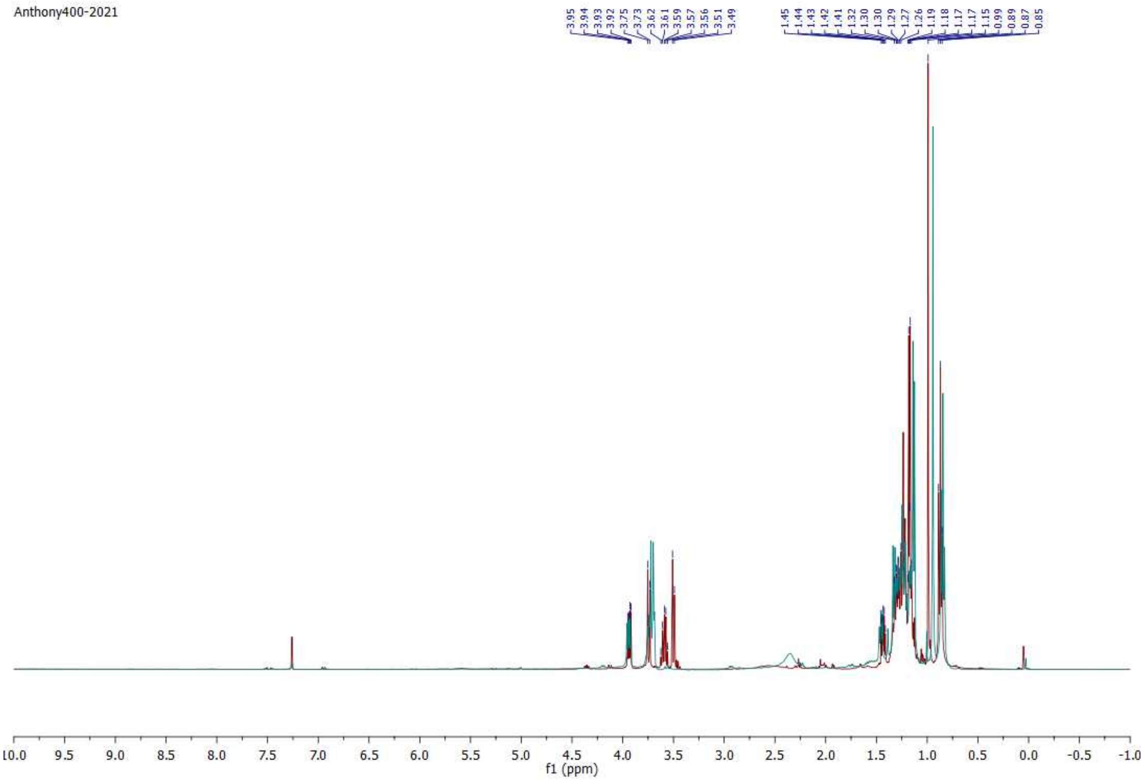

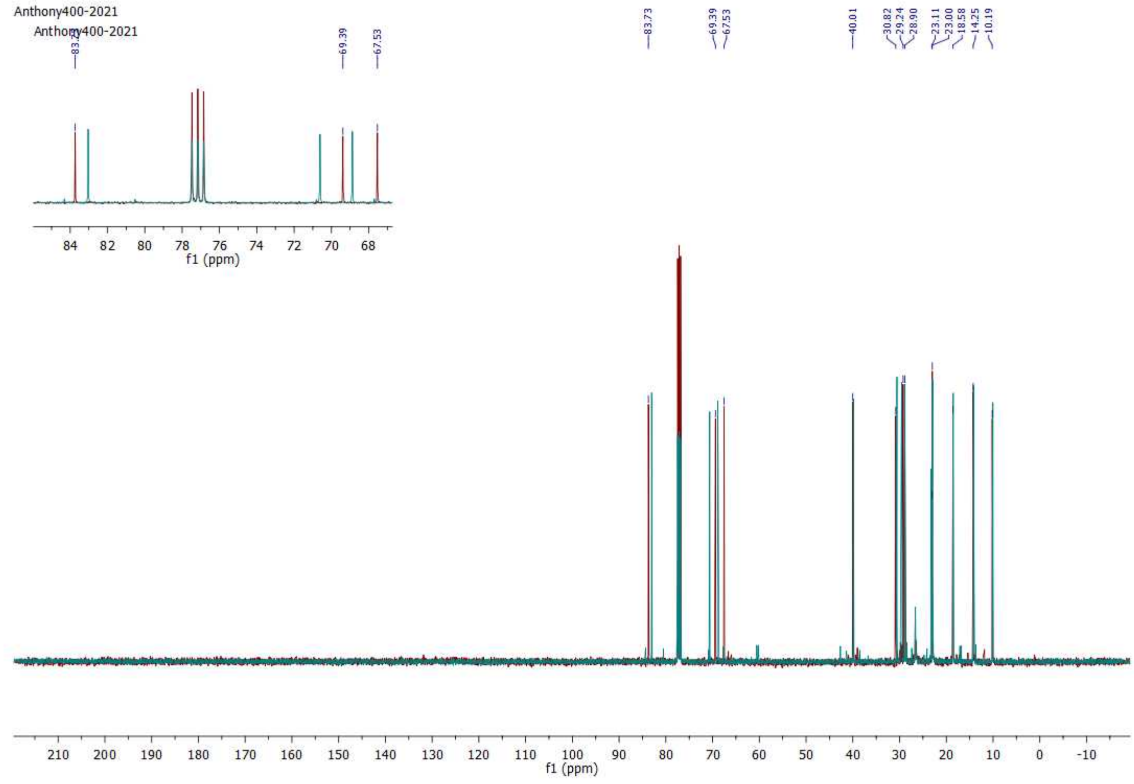

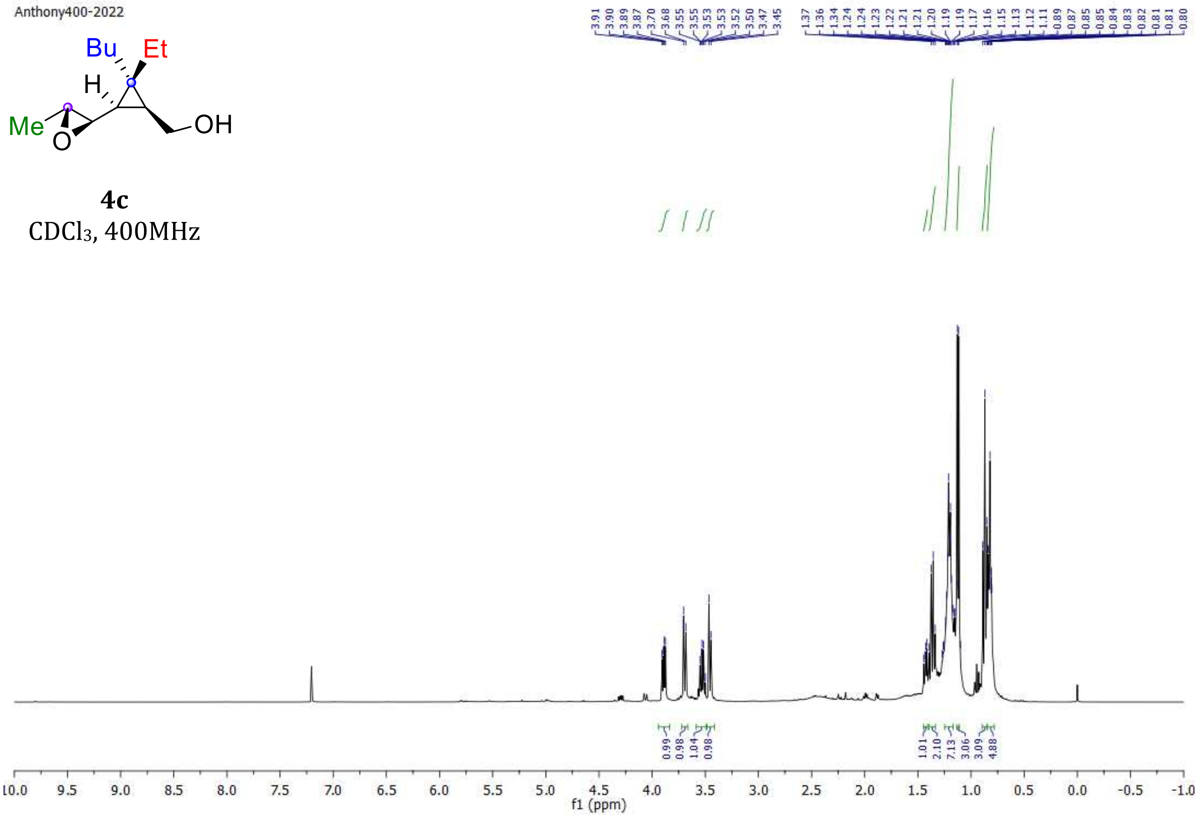
Anthony400-2022
Bu
Et
H
Me
O
OH
4c
CDCl3, 400MHz
3.91 3.90 3.89 3.87 3.70 3.68 3.55 3.55 3.53 3.53 3.52 3.50 3.47 3.45
1.37 1.36 1.34 1.24 1.24 1.23 1.22 1.21 1.21 1.20 1.19 1.19 1.17 1.16 1.16 1.15 1.13 1.12 1.11 0.89 0.87 0.85 0.85 0.84 0.83 0.82 0.81 0.81 0.80
0.99 0.98 1.04 0.98
1.01 2.10 7.13 3.06 3.09 4.88
f1 (ppm)

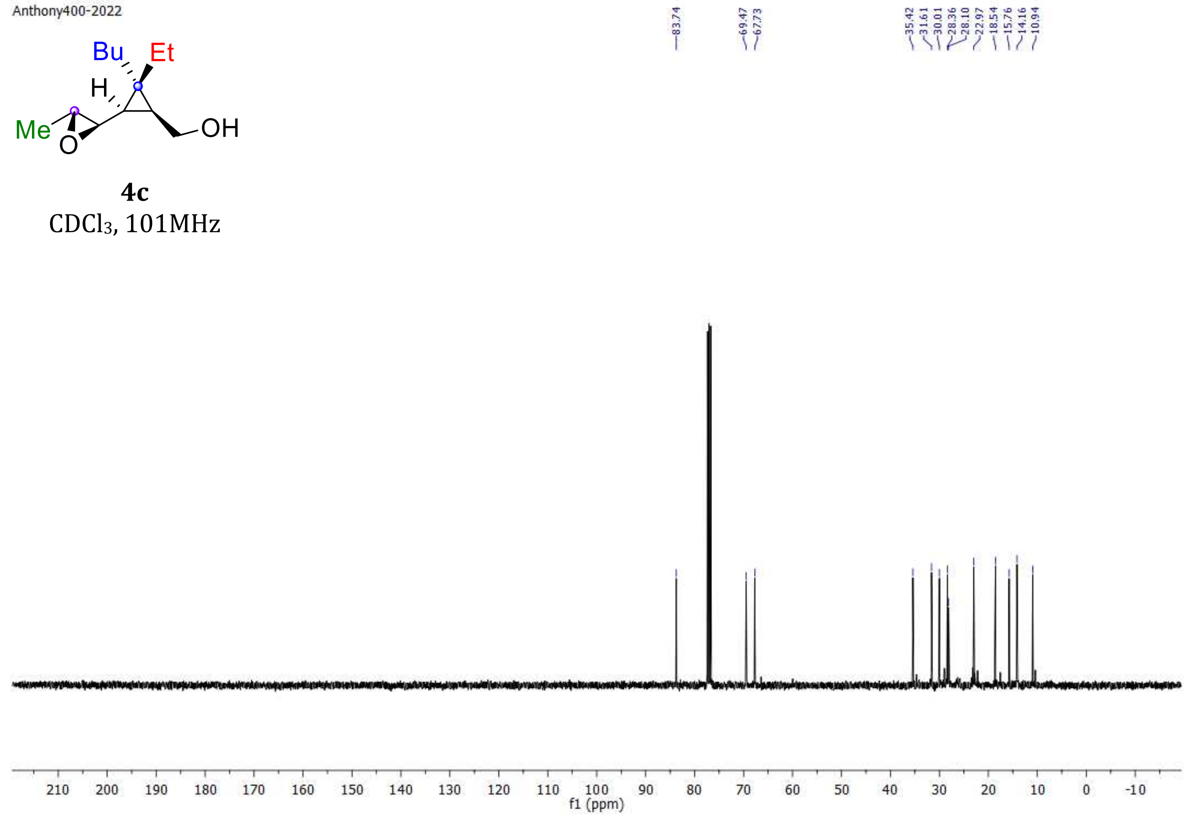
Anthony400-2022
Bu
Et
H
Me
O
OH
4c
CDCl3, 101MHz
83.74
69.47 67.73
35.42 31.61 30.01 28.36 28.10 22.97 18.54 15.76 14.16 10.94
f1 (ppm)

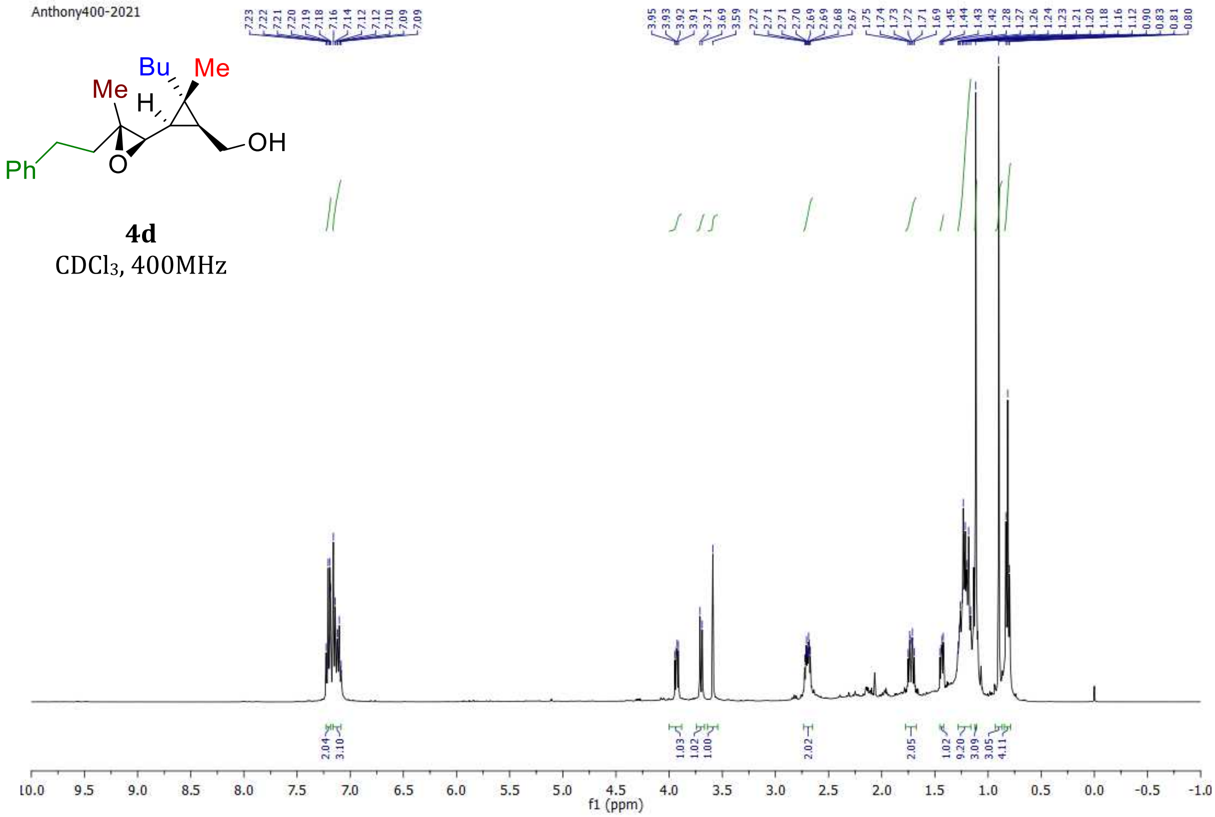

4d
$CDCl_3$, 400MHz

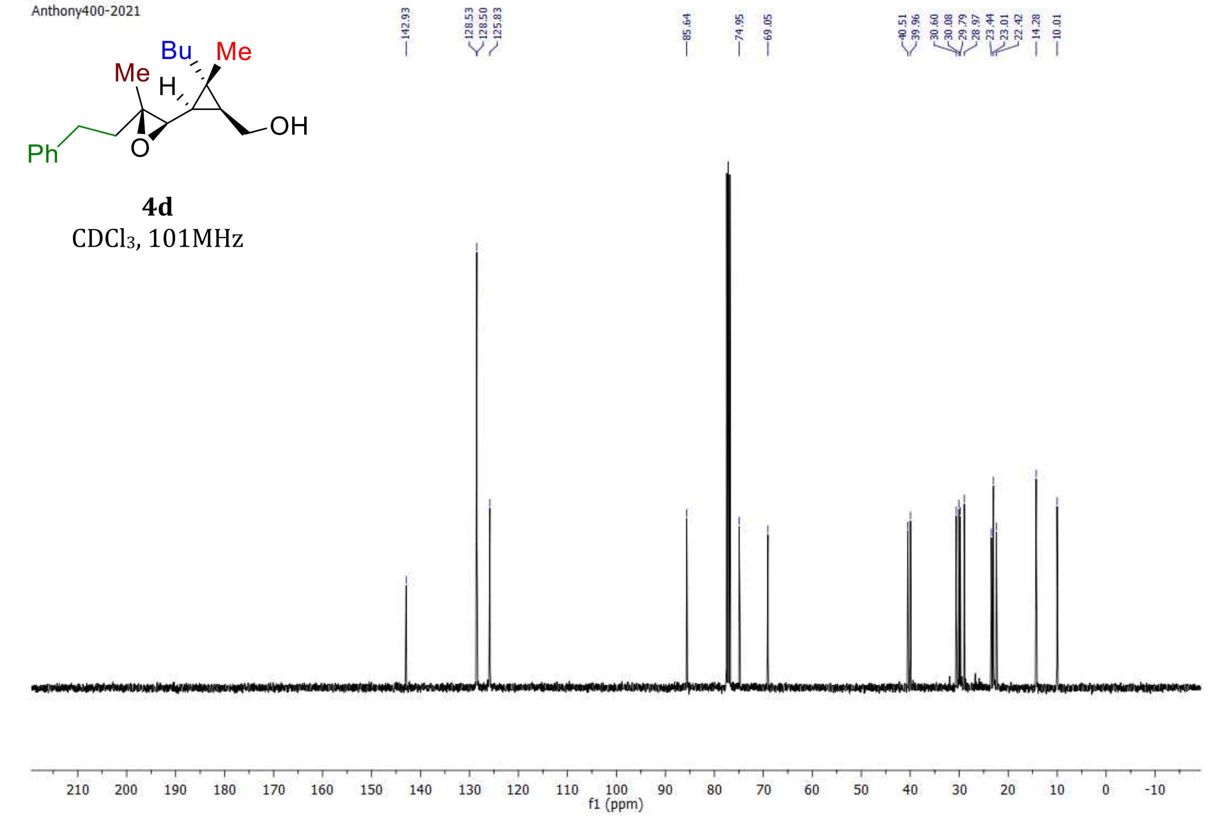

4d
$CDCl_3$, 101MHz
